# Traditional use and management of NTFPs in Kangchenjunga Landscape: implications for conservation and livelihoods

**DOI:** 10.1186/s13002-016-0089-8

**Published:** 2016-05-03

**Authors:** Yadav Uprety, Ram C. Poudel, Janita Gurung, Nakul Chettri, Ram P. Chaudhary

**Affiliations:** Research Centre for Applied Science and Technology, Tribhuvan University, Kirtipur, Kathmandu Nepal; Nepal Academy of Science and Technology (NAST), Khumaltar, Lalitpur Nepal; International Centre for Integrated Mountain Development, Khumaltar, Lalitpur Nepal; Central Department of Botany, Tribhuvan University, Kirtipur, Kathmandu Nepal

**Keywords:** Traditional knowledge, Medicinal plants, Trade, Potential species, NTFP policy, Sustainable use and management

## Abstract

Non-timber Forest Products (NTFPs), an important provisioning ecosystem services, are recognized for their contribution in rural livelihoods and forest conservation. Effective management through sustainable harvesting and market driven commercialization are two contrasting aspects that are bringing challenges in development of NTFPs sector. Identifying potential species having market value, conducting value chain analyses, and sustainable management of NTFPs need analysis of their use patterns by communities and trends at a regional scale. We analyzed use patterns, trends, and challenges in traditional use and management of NTFPs in the southern slope of Kangchenjunga Landscape, Eastern Himalaya and discussed potential implications for conservation and livelihoods. A total of 739 species of NTFPs used by the local people of Kangchenjunga Landscape were reported in the reviewed literature. Of these, the highest number of NTFPs was documented from India (377 species), followed by Nepal (363) and Bhutan (245). Though the reported species were used for 24 different purposes, medicinal and edible plants were the most frequently used NTFP categories in the landscape. Medicinal plants were used in 27 major ailment categories, with the highest number of species being used for gastro-intestinal disorders. Though the Kangchenjunga Landscape harbors many potential NTFPs, trade of NTFPs was found to be nominal indicating lack of commercialization due to limited market information. We found that the unsustainable harvesting and lack of marketing were the major constraints for sustainable management of NTFPs sector in the landscape despite of promising policy provisions. We suggest sustainable harvesting practices, value addition at local level, and marketing for promotion of NTFPs in the Kangchenjunga Landscape for income generation and livelihood improvement that subsequently contributes to conservation.

## Background

Non-timber forest products (NTFPs) are the most important provisioning services people obtain from forest ecosystems [1]. The importance of NTFPs in rural livelihoods and forest conservation has been well recognized as they provide income generation opportunities to millions of people around the world [2–5], and they are also a major source of supplementary food, medicines, fibre, and construction materials [6, 7]. In developing countries, biological resources obtained from forests, mostly NTFPs, may contribute as much as 20–25 % of income to rural people [7]. However, the economic potential of NTFPs is highly contextual and depends on a combined set of socio-cultural, ecological, geo-political, and economic conditions. Nevertheless, access to market/commercialization of NTFPs and sustainable harvesting are two important aspects that need attention for sustainable development of the NTFP sector (also see [8]).

The ecological diversity of the Himalaya makes the area a habitat of a vast range of NTFPs. In the Himalayan region, harvesting NTFPs is a tradition that also contributes significantly to the local economy. Some NTFPs play an important role in traditional health care systems, while others have important cultural values and are sources of food and housing material [9–11]. Among all categories of NTFPs, medicinal plants have received much focus while the contribution of other categories of NTFPs has been overlooked. For example, the contribution of wild edible plants towards food security and income generation has been undervalued in Nepal [12].

Common threats to NTFPs in the Himalayan region include unsustainable harvesting and habitat loss due to land use change, deforestation and over-grazing [13, 14]. Several other challenges have also been identified for sustainable management of NTFPs, such as policies that are ambiguous or poorly implemented due to the lack of resources, lack of comprehensive information on the ecology of the species and its socio-ecological impacts, and poor infrastructure for bioprospecting [15–17]. However, unsustainable harvesting is one of the major issues that affects ecological processes at many levels, from individual and population to community and ecosystem [2, 18]. Commercialization of NTFPs is another important aspect involving different processes such as production, collection, processing, storage, transport, marketing, and sale. Marshall et al. [19] found that product marketing and sale were the most important of all factors that constrained overall success of NTFPs commercialization. However, Ghate et al. [20] found a clear relationship between the degree of proximity to the market and NTFP dependence; remote places with low market access had high NTFPs dependency.

The demand for NTFPs is increasing not only in local markets, but also in international markets. Therefore, some important steps to facilitate integration of NTFPs into the development agenda that benefits local communities include identifying potential species having trade value and conducting research on their ecology and sustainable harvest levels; conducting analyses on value chain and use patterns; and analyzing trends and challenges in marketing and management [21]. Here we focus on these aspects of NTFPs in the Kangchenjunga Landscape within the Eastern Himalaya [22] and explore the implications for conservation and livelihoods.

## Methods

### Study area

**Fig. 1:**
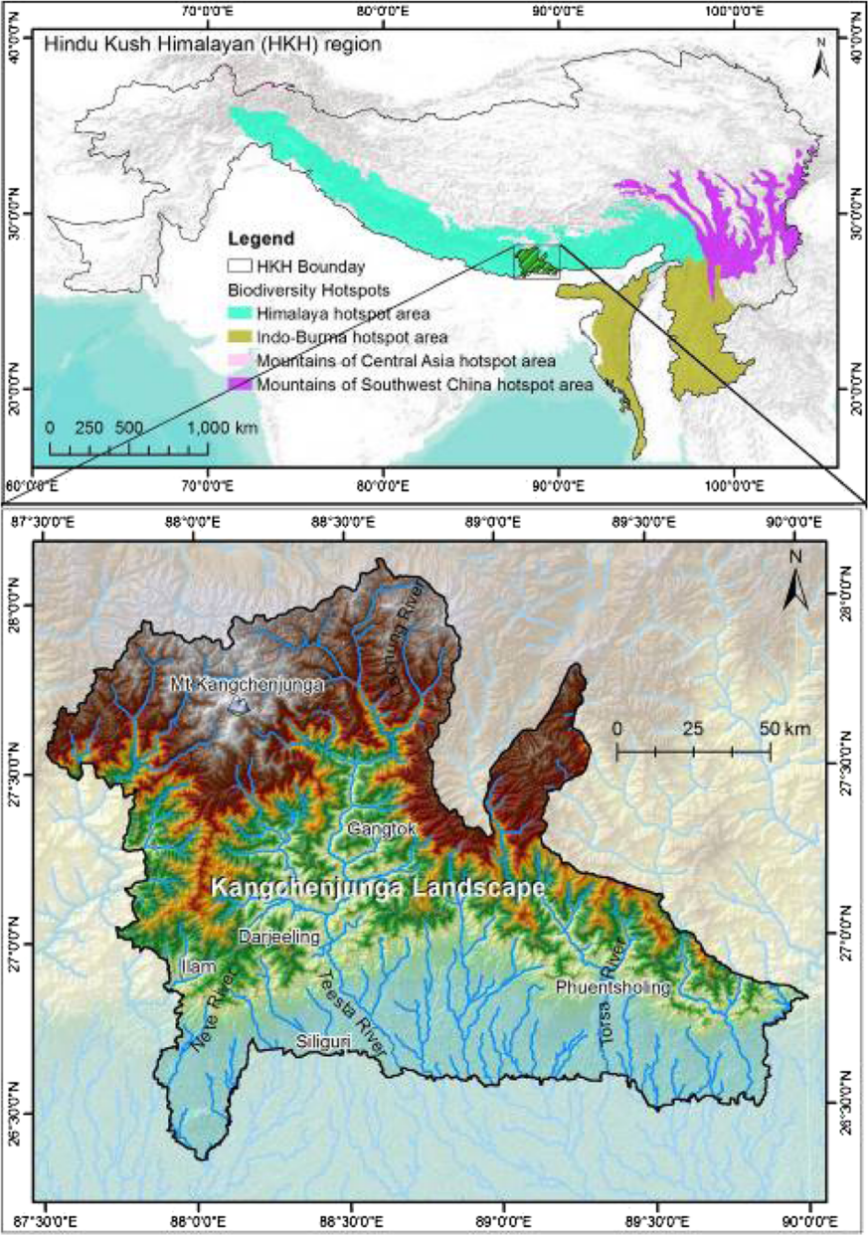
The Kangchenjunga Landscape in the Eastern Himalaya

The Kangchenjunga Landscape is a transboundary landscape shared by Bhutan, India, and Nepal. It is one of the richest landscapes in the Hindu Kush Himalaya (HKH) in terms of cultural and biological diversity and forms part of the Himalaya Biodiversity Hotspot, one of 34 global Biodiversity Hotspots [23]. It extends over 25,000 sq. km within 26^0^ 21′40.49″ to 28^0^7′ 51.25″ North latitudes and 87^0^30′30.67″ to 90^0^ 24′31.18″ East longitudes (Fig. 1). The altitude in the landscape ranges from 50 masl in the south to 8,586 masl, the height of Mount Kangchenjunga–the world’s third highest peak. Vegetation zones in the landscape is comprised of tropical, subtropical, temperate, subalpine, alpine, and nival.

The Kangchenjunga Landscape provides a range of ecosystem services that supports millions of people [24]. However, like many other landscapes worldwide, biodiversity and ecosystems within the landscape face threats mainly from anthropogenic pressures [25] and global climate change [26]. As a result, the people living in the landscape are economically, physically, and socially vulnerable [25, 27].

Recognizing the global and regional significances and challenges that lie within this landscape, the Kangchenjunga Landscape Conservation and Development Initiative (KLCDI) has been initiated by the governments of Bhutan, India and Nepal to achieve biodiversity conservation and sustainable development by applying ecosystem approaches to transboundary landscape management [22]. One of these priority areas is sustainable utilization of NTFPs in the Kangchenjunga Landscape. Several species of high value NTFPs that are also threatened are found in the landscape such as Chiraito (*Swertia chirayita*), Panch aunle (*Dactylorhiza hatagirea*), Kutki (*Neopicrorhiza scrophulariiflora*), Laghupatra (*Podophyllum hexandrum*) and Lauth salla (*Taxus wallichiana*).

### Data collection and analysis

We reviewed scientific studies published in journals and books on traditional uses of NTFPs in the Kangchenjunga Landscape. Various online databases were used (ISI Web of Science, Scopus, and Google Scholar) using specific search terms such as ‘non-timber forest products’, ‘medicinal plants’, ‘wild edible plants’, and ‘Kangchenjunga Landscape’, ‘Nepal’, ‘India’, ‘Sikkim’, ‘Darjeeling’, and ‘Bhutan’. We also explored hard copies of relevant publications. We reviewed a total of 47 publications and one database to enumerate the NTFPs in the Kangchenjunga Landscape. The precision of species identification in this review was dependent on the original source. However, we verified currently accepted name(s) in online nomenclature sources (http://www.theplantlist.org and http://www.tropicos.org). Vernacular names when available have also been provided. A master list was produced providing Linnaean taxonomy, vernacular name(s), mode(s) of use, and reference(s) for each species (Table 1). We also collected trade data and reviewed policy documents on NTFPs of Bhutan, India and Nepal.

**Table 1:** NTFPs used by the local people of the Kangchenjunga Landscape, Eastern Himalaya

SN	Botanical name	Habit	Family	Vernacular name(s)	Distribution (Altitude in meter)	Part(s) used, Use(s), Location and Reference(s)*
	**Angiosperms**					
1	*Justicia adhatoda* L.	Shrub	Acanthaceae	Asuro (Np); Bashakha (Dz); Jantrashi (Me); Khateermu (Sh); Vasakdog (S)	500-1600	**Roots:** Extract taken to cure cholera and epilepsy (KL Nepal: [53]). **Roots, bark and leaves:** Used as insecticide, expectorant, and antispasmodic. Used as remedy for asthma, cough, fever, gonorrhea leprosy, and phthisis (Sikkim: Database)^ǂ^. **Tender shoots:** Used to treat asthma (Jhapa: [66]; Panchthar: [67]). **Leaves:** Extract given orally to cure wheezing in children (Jhapa: [68]). Decoction used in bronchitis, cold, and veneral diseases (Jhapa: [66]). Paste applied on abdomen and vagina just minutes before childbirth for easy delivery (Sikkim: [69]). **Leaves and shoots:** Taken orally against fever, headache and bodyache (Darjeeling: [52]). **Flowers:** Edible (KL Bhutan: [70]) and also used as medicine (KL Bhutan: [71]).
2	*Strobilanthes cusia* (Nees) Kuntze	Herb	Acanthaceae			**Leaves:** Use to extract dye (KL Bhutan: [70]).
3	*Viburnum erubescens* Wall.	Shrub	Adoxaceae	Asaray (Np)	2000-3500	**Seeds:** Edible (Darjeeling: [72]).
4	*Trianthema portulacastrum* L.	Herb	Aizoaceae	Seto Punarnava (Np)	150-300	**Young shoots:** Used as vegetable (Jhapa: [92]).
5	*Alangium salvifolium* (L.f.) Wangerin	Tree	Alanginaceae	Dhela (S); Asare (Np)	150-350	**Bark:** Paste used for abortion and antifertility (Jhapa: [66]). **Fruit:** Edible (Jhapa: [92]).
6	*Achyranthes aspera* L.	Herb	Amaranthaceae	Apamarga, Ulte kuro (Np)	800-2300	**Whole plant:** Juice taken in cough (Panchthar: [67]; Ilam: [73]), dropsy, piles, stomachache (Ilam: [73]) and diarrhoea (Panchthar: [67]).
7	*Achyranthes bidentata* Blume	Herb	Amaranthaceae	Ankhlay Jhar, Datiwan (Np)	200-2100	**Roots and stem:** Juice used as diuretic and also given in rheumatism (Darjeeling: [74]; Sikkim: [75]; Ilam: [73, 76, 77]) and hypertention (Ilam: [73, 76, 77]).
8	*Alternanthera sessilis* (L.) DC.	Herb	Amaranthaceae	Saranchi Saag (Np); Garundi (S)	200-2000	**Roots:** Pounded with seeds of *Foeniculum vulgare* and taken orally to cure piles (Jhapa: [66]). **Leaves and twigs:** Decoction taken to cure fever (Darjeeling: [78]).
9	*Amaranthus spinosus* L.,	Herb	Amaranthaceae	Janum arak (S); Lude (Np)	150-1200	**Roots:** Extract used as diuretic (KL Nepal: [53]). Decoction taken with warm water to check excessive bleeding at post delivery stage (Jhapa: [66]). **Leaves:** Used as vegetable (Jhapa: [92]). Eaten as curry to treat burns, boils and as laxative (Darjeeling: [74]).
10	*Amaranthus* spp.	Herb	Amaranthaceae	Zimtsi (Dz); Naam (Sh)		**Whole plant:** Used as spices (KL Bhutan: [70]).
11	*Amaranthus tricolor* L.	Herb	Amaranthaceae		200	**Leaves:** Used to stop diarrhea (Sikkim: [11]). **Seeds:** Taken to cure gastric problems, fried beaten seeds with butter are given to pregnant women to lessen pregnancy pains (Sikkim: [11]).
12	*Amaranthus viridis* L.	Herb	Amaranthaceae	Gandhari (S); Lude (Np)	150-1200	**Young shoots and leaves:** Used as vegetable (Jhapa: [92]). Juice with sugar taken to cure colic and as laxative (Darjeeling: [74]).
13	*Celosia argentea* L.	Herb	Amaranthaceae	Bhale Phul, Sahastrajadi (Li)	500-1600	**Leaves:** Juice administered orally in diarrhoea and dysentery (Sikkim: [79]).
14	*Allium* sp.	Herb	Amaryllidaceae	Lagok (Dz/Sh)		**Whole plant:** Used as medicine (KL Bhutan: [71]).
15	*Allium wallichii* Kunth	Herb	Amaryllidaceae	Wild Garlic (Eg); Palengu (Gr); Gokpa, Koje, Ri Gokpa (Km, Sh); Ban Lasun, Ban Pyaj, Jimbu, Jimbujhar (Np); Bathatuva (Ri)	2300-4800	**Plant:** Used in digestion (Sikkim: [69]). **Bulbs and aerial parts:** Used as spice (Sikkim: [10, 80], Database; Taplejung: [81, 82]; Sikkim: Database). **Bulbs**: Boiled, fried in clarified butter and taken in cholera and diarrhea; chewed to treat cough, colds, and altitude sickness (Taplejung: [83]). **Bulbs and flowers:** Use to treat gastric disorders (Sikkim: [75]).
16	*Choerospondias axillaris* (Roxb.) B. L. Burtt & A. W. Hill	Tree	Anacardiaceae	Nepali Hug Plum (Eg); Phindruwa (Li); Lapsi (Np); Amali (Nw); Pindumsi, Pintupsi (Ri); Nying Sho Sha (Tb)	1200-1900	**Fruits:** Edible (KL Bhutan: [70]). Edible and also used to make pickle (Darjeeling: [80], Sikkim: [10, 47, 72, 95]; Ilam: [94]; Taplejung: [88]). Used to treat cough, cold, tonsillitis, diabetes, worm infestation, and heart ailment [87].
17	*Lannea coromandelica* (Houtt.) Merr.	Tree	Anacardiaceae	Doka (S)	100-1400	**Bark:** Paste applied around bone fracture to set bone (Jhapa: [66]).
18	*Mangifera indica* L.	Tree	Anacardiaceae	Thaijau (Me); Ul (Np)	300-700	**Plant:** Effective for jaundice patient (Darjeeling: [105]). **Fruits:** Edible (KL Bhutan: [70]). Green unripe fruit skin crushed and drunk to get relief from stomachache (Jhapa: [68]). **Bark:** Given with bark of *Anthocephalus chinensis, Madhuca longifolia* and *Gmelina arborea* in diarrhea and dysentery (Jhapa: [66]). **Leaves:** Young leaves chewed during cough and sore throat (Sikkim: [79]).
19	*Mangifera sylvatica* Roxb.	Tree	Anacardiaceae	Chuchiam, Kathorkung (Lp)		**Fruits:** Used as laxative (Sikkim: [85]).Used to make sour pickle (Ilam: [94]).
20	*Rhus chinensis* Mill.	Tree	Anacardiaceae	Bhakimlo (Np); Chokashing (Dz); Roptangshing (Sh);		**Fruits:** Edible (KL Bhutan: [70]). Used as medicine (KL Bhutan: [71]). **Flowers and fruits:** Use to extract dye (KL Bhutan: [70]).
21	*Rhus javanica* Miller	Tree	Anacardiaceae	Tibru, Tsakshing (Gr); Rushi (Km, Sh); Yaseba, Isewa (Li); Bhaki Amilo, Bhakimlo, Dudhe Bhalayo (Np); Mahada, Oksarempo, Osreksi (Ri); Bokumba, Thaksing (Sh); Tibi Sing (Tm); Da Trig (Tb)	1000-2700	**Fruits:** Edible and also used in the treatment of diarrhea, dysentery (Taplejung: [88]; Panchthar: [67]; Ilam: [73]) and stomach problems (Taplejung: [88]; Ilam: [73]). Dried and extract used in diarrhea, swellings and wounds (Sikkim: Database). Juice used as food preservative; juice boiled with water and mixed with raw egg and the mixture given to treat diarrhea (Sikkim: [11]) and dysentery (Darjeeling: [80]; Sikkim: [11]). Juice administered orally during blood dysentery (Sikkim: [79]). Boiled and isolated vinegar used to make pickles (Ilam: [94]).
22	*Semecarpus anacardium* L.f.	Tree	Anacardiaceae	Soso (S); Rani bhalayo (Np); Bhalau (Me)	150-1200	**Roots:** Used to treat skin infection (Sikkim: [11, 75]). **Bark:** Decoction given to animals to remove worms (Sikkim: [11]). **Latex:** Used as antitetanus (Jhapa: [66]). **Fruits:** Edible (Jhapa: [92]). Also used to treat headache (Jhapa: [68]). **Seeds:** Used as medicine (KL Bhutan: [71]). Seed oil taken in cough and colds (Jhapa: [66]).
23	*Spondias pinnata* (L. f.) Kurz	Tree	Anacardiaceae	Amaro (Np)	300-1400	**Flowers:** Used to make curry and for flavoring (Ilam: [94]).
24	*Toxicodendron hookeri* (K.C. Sahni & Bahadur) C.Y. Wu & T.L. Ming	Tree	Anacardiaceae	Tarsishing (Sh)		**Fruits:** Edible (KL Bhutan: [70]).
25	*Annona squamata* L.	Tree	Annonaceae	Saripha (Np); Mandargom (S)	500-900	**Bark:** Juice drunk to control fever (Jhapa: [68]). **Leaves and seeds:** Used to kill lice (Panchthar: [67]). Thalamus is used as antifertility (Jhapa: [66]). **Seeds**: Useful in abortion and menstrual disorders (Jhapa: [66]). **Roots and seeds:** Paste applied on forehead during headache (Sikkim: [79]).
26	*Angelica cyclocarpa* (C.Norman) M.Hiroe	Herb	Apiaceae			**Roots:** Used as medicinal (KL Bhutan: [71]).
27	*Angelica sinensis* (Oliv.) Diels	Herb	Apiaceae			**Roots:** Used as medicinal (KL Bhutan: [71]).
28	*Carum carvi* L.	Herb	Apiaceae	Caraway (Eg); Chir (Gr-Mn); Ban Jeera (Np); Sushavi (Sn); Chhonyo, Khoda (Sh,Wi)	2500-5100	**Fruits:** Used as medicine (KL Bhutan: [71]). **Leaves:** Used as vegetable (Taplejung: [87]).
29	*Centella asiatica* (L.) Urb.	Herb	Apiaceae	Ghortaapre, Gol patta, Gora taprey, Goltaprey (Np); Dalka chatumana (S)	500-2100	**Plant:** Used to cure heating and tenderness of limb skin (Jhapa: [68]). Juice taken orally to get rid of pneumonia, fever (Sikkim: [75]), asthma (Jhapa: [68]; Ilam: [76, 77]; Sikkim: Database, [69]), mental tension, urinary problems, and stomachache (Ilam: [76, 77]; Jhapa: [68]; Sikkim: [75]). Infusion massaged on the forehead to relieve from headache (Jhapa: [66]). **Leaves:** Used for skin disease and improving memory (Sikkim: Database). Leaves and young shoots taken to cure tonsil (Darjeeling: [52]). Aerial parts mixed with young shoots of *Justicia adhatoda* and taken against diarrhoea (Darjeeling: [86])*.*
30	*Coriandrum sativum* L.	Herb	Apiaceae	Dhaniya (Np)	1000-2500	**Seeds:** Used as spice (Ilam: [76]) and in flatulence (Darjeeling: [91]). **Leaves:** Used in flatulence (Darjeeling: [91]). Green leaves used to enhance aroma on food (Ilam: [76]). **Plant:** Used in cough, bronchitis, reheumatism and urinary problem (Ilam: [76]).
31	*Cortia depressa* (D.Don) C. Norman	Herb	Apiaceae	Nigale Sag (Np); Gholo, Ghundu, Kholo, Nhopchhema (Sh); Tangkyun (Sh, Tb); Ghundu (Wl)	3600-5100	**Leaves:** Tender leaves used as vegetable (Taplejung: [88]). **Seeds:** Used as spice (Taplejung: [81]).
32	*Heracleum nepalense* D. Don	Herb	Apiaceae	Sunaga, Habluwa (Km); Samben (Lp); Chimphing (Np); Bhote-Jeera, Nafo (Np-Dl); Chimping (Np-Tb); Phaki, Thar (Sh); Chimbing, Chimping (Sh, Wl); Chapho Karpo, Zira Goepa (Tb)	1800-3700	**Plant:** Used as good winter fodder (Taplejung: [81, 87]). **Roots:** Given to cattle as tonic; juice and roasted seeds taken in cough and diarrhoea (Panchthar: [67]; Ilam: [73, 77]). **Flowers and fruit:** Suggested to cure influenza and body ache (Sikkim: [75]). **Fruit**: Used as pickle and also in typhoid, nausea, and vomiting (Darjeeling: [78]). **Seeds:** Used in case of bodyache, headache, and faint (Panchthar: [67]; Ilam: [73]).
33	*Heracleum wallichii* DC.	Herb	Apiaceae	Chimphing (Np)	3600-4100	**Roots:** Used as medicine (KL Bhutan: [71]).Used as tonic and aphrodisiac (Darjeeling: [78]). Juice taken to cure diarrhea (Sikkim: [11]). **Seeds:** Taken in diarrhea and gastric disorders (Sikkim: [75]), used as *chatni* (Sikkim: [11]). Decoction of dried seeds taken in influenza (Darjeeling: [78]; Sikkim: [11]).
34	*Heracleum candicans* Wall. ex DC.	Herb	Apiaceae			**Whole plant:** Used as medicinal (KL Bhutan: [71]).
35	*Heracleum dissectifolium* K.T. Fu	Herb	Apiaceae			**Roots:** Used as medicine (KL Bhutan: [71]).
36	*Hydrocotyle himalaica* P. K. Mukh.	Herb	Apiaceae	Golpata (Np)	1500-2500	**Plant:** Extract taken to cure pneumonia and throat infection (Sikkim: [75]).
37	*Hydrocotyle javanica* Thunb.	Herb	Apiaceae	Golpatta (Np)		**Plant:** Taken to cure throat problem (Sikkim: [75]).
38	*Hydrocotyle sibthorpioides* Lam.	Herb	Apiaceae	Tike Ghor Tapre (Np)	600-2500	**Plant:** Useful in treatment of migrant and to improve memory (Panchthar: [67]).
39	*Selinum wallichianum* (DC.) Raizada & Saxena	Herb	Apiaceae	Sunaga (Km); Bhutkesh (Np); Bhatauri, Dhaneli, Kalo Chhetaro (Np-Dl); Tunak (Tb); Chyadukpa (Sh, Wl)	2700-4800	**Roots and fruits:** Used for cuts, wounds, colic, gastritis, and intestinal pain (KL Nepal: [83])**. Roots:** Smoke inhaled in case of faint, fever, and headche. Also used as fodder (Taplejung: [81]). Decoction taken to get relief from pain and fever (Ilam: [76]). **Flowers and leaves:** Smooked to treat headache (KL Nepal: [53]). **Leaves:** Leaves, aromatic, carminative. **Fruits:** Used in skin diseases and scabies (Sikkim: Database).
40	*Alstonia scholaris* (L.) R. Br.	Tree	Apocynaceae	Chhatiwan (Np); Chhatni (S)	100-1400	**Bark:** Used in malarian fever and also given to lactating mothers for increasing milk (Jhapa: [66]). Extract used to treat piles (KL Nepal: [53]). Filtered bark juice used to cure leanness in man (Jhapa: [68]). Powder mixed with common salt and given to cattle to cure fever (Sikkim: [97]). **Bark, latex and flowers:** Used as tonic and in fever and leucoderma (Sikkim: [97]).
41	*Catharanthus roseus* (L.) G. Don	Shrub	Apocynaceae	Barhamase Phool, Sadabahar (Np); Gofatbhiwar (Me)	150-1500	**Plant:** Anti cancer and antitumour (Sikkim: Database). **Leaves:** Used as diuretic (Sikkim: Database). **Flowers:** Eaten to maintain sugar level for diabetic patients (Jhapa: [68]).
42	*Holarrhena pubescens* (Buch.-Ham.) Wall. ex G. Don	Shrub	Apocynaceae	Ban Khirro, Khuria, Anley Khirrn, Madhese Khirro (Np); Hat (S)	100-1500	**Plant:** Powder or decoction taken to treat acute diarrhea and dysentery (Darjeeling: [86]; Sikkim: [11, 85]). **Bark**: Powdered with black pepper and taken to cure cough, cold, fever, diarrhea, and dysentery (Jhapa: [66]). Juice taken in amoebic dysentery (Sikkim: [11, 85]). **Bark and seeds:** Useful to treat gastric troubles and have anthelmintic property (Panchthar: [67]). **Fruit:** Used as medicine (KL Bhutan: [71]). **Stem**: Bark powder or decoction given to livestock to treat constipation, problems during stool passing and dysentery (Sikkim: [84]).
43	*Ichnocarpus frutescens* (L.) R. Br.	Climber	Apocynaceae	Dudilata (S); Dudelaharo (Np)	150-900	**Leaves:** Extraction used in stomach pain (Jhapa: [66]). **Stem**: Extraction used in toothache and gum swelling (Jhapa: [66]).
44	*Rauvolfia serpentina (L.) Benth. Ex Kurz.	Herb	Apocynaceae	Kharokha (Me); God (S)	100-900	**Young shoots:** Extract given to cure pneumonia (Jhapa: [68]). **Roots, stems and leaves:** Used to treat fever (Panchthar: [67]). **Roots:** Extract used as medicine (KL Bhutan: [71]). Useful in insomnia (Sikkim: Database), high blood pressure, and snake bite (Jhapa: [66]; Sikkim: Database). Extract taken in stomach pain and intestinal worms (Jhapa: [66]). Powder administered orally as antidote to snake-bite (Sikkim: [84]).
45	*Vincetoxicum hirundinaria* Medik.	Herb	Apocynaceae			**Fruits:** Used as medicine (KL Bhutan: [71]).
46	*Acorus calamus L.	Herb	Araceae	Sweet Flag (Eg); Sadakppa (Li); Ruklop (Lp); Bojho (Np); Chhowas (Ri); Vacha (Sn); Chota, Jota (Gr-Mn); Suda, Syueda (Sh); Shete (Tm); Tshedak (Sh); Shudag (Tb)	1700-2300	**Rhizomes:** Used in treatment of epilepsy and other mental ailments, chronic diarrhoea (Darjeeling: [86]; Sikkim: [69]), colic pains and as a brain tonic (Sikkim: [69]). Used to cure cold, cough (Jhapa: [68]; Sikkim: [11, 79]; Ilam: [73]), sore throat, bronchitis, tonsil, toothache, headache (Jhapa: [68]; Ilam: [73]), bodypain (Jhapa: [66]), scabies, sinusitis (Panchthar: [67]) and also as vermifuge and antispasmodic (Sikkim: [75]). Used in skin disease (Darjeeling: [80]; Sikkim: [11, 79, 89]), malaria and asthma (Darjeeling: [80, 89]). Juice used as insecticide (Panchthar: [67]; Sikkim: [75]). Decoction taken as antipyretic (Sikkim: [11, 69]; Darjeeling: [78]) and also to treat rheumatism (Darjeeling: [78]). Pounded with the rhizome of *Curcuma zedoaria* and given in ulcers and abdominal pains (Jhapa: [66]). Extract taken to cure measles (Sikkim: [79]). Crushed and boiled with salt and decoction used to massage for fortnight (Darjeeling: [74]). Paste applied as ointment on wounds in cattle, also administered in indigestion in cattle (Sikkim: [84]).
47	*Acorus gramineus* Aiton	Herb	Araceae	Silijam (Dz), Bortsl (Sh)		**Rhizomes:** Used as medicine (KL Bhutan: [71]).
48	*Alocasia macrorrhizos* (L.) G. Don	Herb	Araceae	Man kachui (S); Karkalo (Np).	100-800	**Petioles and tuber:** Edible as vegetable (Jhapa: [92]). **Young petioles**: Cooked with *Colocasia esculenta* and taken orally in cold and cough (Jhapa: [66]).
49	*Alocasia* spp.	Herb	Araceae			**Tubers:** Edible (KL Bhutan: [70]).
50	*Amorphophallus paeoniifolius* C. Y. Wu ex H. Li, Y. Shiao & S. L. Tseng	Herb	Araceae	Pindo (S); Ol (Np)	100-800	**Tubers and petioles:** Used as vegetable (Jhapa: [92]).
51	*Arisaema costatum* (Wall.) Mart. ex Schott	Herb	Araceae	Glatli (Gr); Asek kaki (Li); Dhakayo, Jangali Makai (Np); Thwa (Sh)	1900-2800	**Leaves:** Dried leaves boiled and cooked as vegetable (Taplejung: [87]). Young shoots used to make curry and corn eaten after repeated boiling (Ilam: [94]).
52	*Arisaema griffithii* Schott	Herb	Araceae	Asek Makai, Yakla Khomba (Li); Dhokayo (Np); Doka Banko (Np-Dl); Thwa (Sh); Thwa (WI)	2400-3500	**Leaves:** Sundried, stored and consumed as vegetable in winter; dried leaves are also used to make *Sinki* (dried fermented leaves) (Sikkim: [98]; Taplejung: [81, 88]).
53	*Arisaema intermedium* Blume	Herb	Araceae	Sarpa Makai (Np)	2600-3400	**Roots:** Juice or paste taken in ulcer and fever (Ilam: [73, 77]).
54	*Arisaema jacquemontii* Blume	Herb	Araceae	Banko (Np)	2700-4700	**Roots:** Juice taken orally to treat menstrual disorders, toothache, and pain (Ilam: [73, 77]).
55	*Arisaema* sp.	Herb	Araceae	Dowo (Dz), Rungenengsae (Sh)		**Bulbs:** Used as medicine (KL Bhutan: [71]).
56	*Caladium bicolor* (Ait.) Vent.	Herb	Araceae	Dungasaru (S)		**Rhizomes:** Pounded and applied on body to relieve from bodyache (Jhapa: [66]).
57	*Scindapsus officinalis* (Roxb.) Schott	Climber	Araceae	Kammare lahara (Np); Dari jappa (S)	200-300	**Fruit:** Pounded and applied on rheumatism and bodyache (Jhapa: [66]).
58	*Typhonium trilobatum* (L.) Schott	Herb	Araceae	Nirbish (S)	450	**Rhizome:** Paste applied externally to cure rheumatism (Jhapa: [66]).
59	*Aralia cachemirica* Decne.	Shrub	Araliaceae	Dal kabro (Np); Dumbak (Wl)	2400-4200	**Plant:** Powder contains high nutrient and generates heat in the body of animal (Taplejung: [81]). **Roots:** Used to treat cuts and contraction of muscles (Panchthar: [67]).
60	*Brassaiopsis hainla* (Buch.-Ham. ex D. Don) Seem.	Tree	Araliaceae	Chuletro (Np, Li)	1000-1900	**Roots:** Administered orally in case of dysentery (Sikkim: [79]).
61	*Hedera nepalensis* K. Koch	Shrub	Araliaceae	Ivy (Eg); Dudela (Np)	2000-3200	**Plant:** Juice taken orally as antispasmodic (Sikkim: [85]).
62	*Helwingia himalaica* Hook. f. & Thomson ex C. B. Clarke	Shrub	Araliaceae		2100-2700	**Fruits:** Enhance fertility (Sikkim: [75]).
63	*Macropanax undulatus* (Wallich ex G. Don) Seemann	Tree	Araliaceae	Chenday (Np)	400-2200	**Bark:** Administered against diabetes (Darjeeling: [52]).
64	*Merrilliopanax alpinus* (C. B. Clarke) C. B. Shang	Tree	Araliaceae			**Young shoots:** Useful in gastric disorders (Sikkim: [75]).
65	*Panax pseudo-ginseng* subsp. *himalaicus* H. Hara	Herb	Araliaceae	Mangan, Panchpattery (Np)		**Roots:** Taken to reduce fever, indigestion, and vomiting; also used as tonic (Sikkim: [75], Database).
66	*Panax pseudo-ginseng* Wall.	Herb	Araliaceae	Panchapattey (Np)		**Roots:** Used as medicine (KL Bhutan: [71]). Used as stimulant, combating general debility, headache, vomiting, expectorant, carminative, tonic, in blood pressure (Sikkim: [69]) and weakness (Ilam: [76]; Sikkim: [69]). Juice given to cure liver disorders, colic, fever and menstrual disorders (Darjeeling: [74]).
67	*Pentapanax leschenaultii* (DC.) Seem.	Tree	Araliaceae	Chinde (Np)	1600-3700	**Leaves:**Tender leaves after boiling used as curry (Ilam: [94]).
68	*Areca catechu* L.	Tree	Arecaceae			**Fruit/seed:** Used as medicine (KL Bhutan: [71]).
69	*Borassus flabellifer* L.	Herb	Arecaceae	Tari (S)		**Flowers:** Juice taken to quench the thurst (Jhapa: [66]).
70	*Calamus acanthospathus* Griff.	Climber	Arecaceae	Cane (Eng)		**Shoots:** Edible (KL Bhutan: [70]). Edible and also used as a substitute for rope, as cable for suspension bridges, for wickerwork, baskets, and containers. Thicker cane used for makingfurniture frames, walking sticks and umbrella handles (KL Bhutan: [100]).
71	*Calamus erectus* Roxburgh	Climber	Arecaceae			**Shoots:** Edible (Bhutan: [99]).
72	*Calamus latifolius* Roxb.	Climber	Arecaceae		600	**Shoots:** Edible (KL Bhutan: [70]). **Leaves:** Juice used to cures eye diseases (Sikkim: [11]).
73	*Calamus tenuis* Roxb.	Climber	Arecaceae	Cane (Eng)		**Shoot:** Edible (KL Bhutan: [70]). Used to make household items, such as mats, screens and furnitures (KL Bhutan: [100]).
74	*Caryota urens* L.	Tree	Arecaceae	Rangbhang (Np)		**Stem and buds:** Inner core pith and terminal bud consumed as vegetable (Ilam: [94]).
75	*Phoenix acaulis* Roxb. ex Buch.-Ham.	Tree	Arecaceae	Betgera (Np)	1400	**Fruits:** Raw fruits used to make vegetable curry (Ilam: [94]).
76	*Phoenix sylvestris* Roxb.	Tree	Arecaceae	Thakal (Np)	150-1500	**Stem:** Soft pith eaten raw (Ilam: [94]).
77	*Plectocomia himalayana* Griffith	Herb	Arecaceae	Patsha (Bhut)	1500-2500	**Young shoots:** Taken as vegetable (Bhutan: [99, 102])
78	*Aristolochia griffithii* Hook.f. & Thomson ex Duch.	Climber	Aristolochiaceae			**Flowers:** Used as medicine (KL Bhutan: [71]).
79	*Aristolochia indica* L.	Climber	Aristolochiaceae	Godh (S)		**Roots and leaves:** Paste applied in stomachache and as an antidote in snake bite and scorpion sting (Jhapa: [66]).
80	*Asclepias curassavica* L.	Herb	Asclepiadaceae	Khorsani Kose Phul (Np)	700-1500	**Roots:** Used to treat cancer, warts, and snake bite (Panchthar: [67]).
81	*Calotropis gigantea* (L.) Dryand.	Shrub	Asclepiadaceae	Ankh (Np); Gogando-bimtang (Me); Akona (S)	100-1000	**Latex and Leaves:** Used in sprain and swelling (Jhapa: [66]; Panchthar: [67]; Sikkim: Database). **Roots**: Extraction given in fever and applied on chest and abdomin to relieve pains (Jhapa: [66]). **Latex:** Used as medicine (KL Bhutan: [71]). Applied on burns (Jhapa: [66]) and for ring worm (Jhapa: [68]).
82	*Hoya longifolia* Wall. ex Wight	Shrub	Asclepiadaceae	Wax Plant (Eg)	1400-2300	**Leaves:** Applied on burns (Panchthar: [67]).
83	*Marsdenia roylei* Wight,	Climber	Asclepiadaceae	Baahuni Lahara (Np)	1400-2400	**Plant:** Used for cooling and alternative effect in gonorrhea (Sikkim: Database). **Roots, leaves and fruit:** Decoction used to relieve burning sensation of the genitals (Sikkim: [11]).
84	*Marsdenia tenacissima* Weight & Arn.	Climber	Asclepiadaceae	Bahuni Lahara, Sunamari (Np), Kamtiongrik (Lp)		**Roots:** Juice taken daily as purgative (Sikkim: [85]).
85	*Marsdenia tinctoria* R. Br.	Climber	Asclepiadaceae	Kali Lahara, Ryom (Np)		**Leaves:** Juice taken for stomachache (Sikkim: [85]).
86	*Campylandra aurantiaca* Baker	Herb	Asparagaceae	Nakima (Np)	1900-2900	**Roots:** Stocks given orally in case of food poisoning (Darjeeling: [52]). **Inflorescence:** Powdered and taken with water to relieve body pain (Sikkim: [11]). Given in food poisoning (Darjeeling: [74]). **Flowers:** Used as appetizer and taken in diabetes (Sikkim: [69, 79], Database). Taken as curry (Sikkim: [89]).
87	*Chlorophytum arundinaceum* Baker	Herb	Asparagaceae	Turam (S)	500-1200	**Roots:** Powder taken to relieve from body weakness (Jhapa: [66]).
88	*Eucomis regia* (L.) Aiton	Herb	Asparagaceae	Lily (C)		**Bulb:** Used as medicinal (KL Bhutan: [71]).
89	*Tupistra nutans* Wall. ex Lindl.	Herb	Asparagaceae	Nakima (Np)		**Flower:** Taken as appetizer and in diabetes (Sikkim: Database). **Inflorescence:** Powdered and taken to relieve from body pain (Sikkim: [11]).
90	*Acmella calva* (Candolle) R. K. Jansen	Herb	Asteraceae	Mareti (Np)	1000-1900	**Fruit:** Juice taken orally for headache and stomachache [66, 77], toothache (Darjeeling: [74]; Ilam: [73, 77]) and sore mouth (Darjeeling: [74]).
91	*Ageratina adenophora* (Spreng.) R.M. King & H. Rob.	Herb	Asteraceae	Banmara (Np)	850-2500	**Plant:** Juice applied on fresh cut (Darjeeling: [74]; Ilam: [73]; Sikkim: [11, 75]) and also taken orally in fever (Ilam: [73]). **Leaves:** Taken orally in dysentery (Darjeeling: [52]).
92	*Ageratum conyzoides* L.	Herb	Asteraceae	Ilamejhar (Np)	200-2000	**Whole plant:** Used as antidote (KL Nepal: [53]). **Leaves:** Juice applied on wound (Sikkim: [11]; Panchthar: [67]) and also used as anthelmintic (Panchthar: [67]). Decoction used as antipyretic (Darjeeling: [78]), and also in diarrhea, dysentery, colic, and flatulence (Sikkim: [11]).Tenders chewed to cure diarrhoea and dysentery. **Flowers**: chewed to treat throat pain (Sikkim: [79]).
93	*Ajania tibetica* (Hook.f. & Thomson) Tzvelev	Shrub	Asteraceae			Used as incense (KL Bhutan: [70]).
94	*Anaphalis adnata* Wall. ex DC.	Herb	Asteraceae	Buki phul (Np)	800-3200	**Leaves:** Juice applied on fresh cuts and wounds (Ilam: [73, 77]).
95	*Anaphalis triplinervis* (Sims) C. B. Clarke	Herb	Asteraceae	Bhukiphul (Np)	100-2300	**Flower:** Paste applied regularly to cure skin problems (KL Nepal: [53]).
96	*Artemisia dubia* Wall. ex Besser	Herb	Asteraceae	Pati, Titepati, Titaypati (Np); Sibuma, Sungmara (Ri); Chhaphung (Sh); Khenpa (Tb); Sangsin Khemba (Wl)	1200-3400	**Whole plant:** Used in ritual ceremony (Sikkim: [11]). **Leaves:** Used to treat pains and possess anthelmintic properties (Panchthar: [67]).Crushed fresh leaves uesd to open decongestant sinuses and to stop nasal bleeding (Taplejung: [81]; Sikkim: [11], Database). Extract used on cuts and bruises (Sikkim: [11], Database). Supposed to possess detergent effect and used as cleansing agent (Sikkim: Database). Taking bath of leaves juice cures skin allergies and leaves chewed to treat mouth ulcer (Sikkim: [11]). Used as deobstruent, antispasmodic, obstructed menses and hysteria (Darjeeling: [80]).
97	*Artemisia indica* Willd.	Herb	Asteraceae	Namyohoba (Li); Tuknil (Lp); Titepati (Np); Tompe (Sh)	300-2400	**Tender shoots and leaves**: Used to treat inflammation (Taplejung: [87]). **Leaves and flowers**: Juice used to treat injuries (Sikkim: [75]). Juice taken in asthma, gastritis and skin disease (Ilam: [76, 77]).
98	*Artemisia nilagirica* (C.B. Clarke) Pamp.	Herb	Asteraceae	Titepati (Np)		**Shoots and leaves:** Used to cure mouth ulcer; paste applied externally on forehead during dizziness and headache (Darjeeling: [52]).
99	*Artemisia sieversiana* Ehrh.ex Willd.	Shrub	Asteraceae			**Whole plant:** Used as medicine (KL Bhutan: [71]).
100	*Artemisia vulgaris* L.	Herb	Asteraceae	Titepati (Np); Titeypati (Li)	1500-3800	**Leaves:** Used to treat nose bleeding (Sikkim: [69, 79, 91]) nervous and spasmodic affections, asthma and the disease of the brain (Sikkim: [69]). Tender leaves chewed cures mouth ulcers; crushed leaves mixed with water and taken bath cures skin allergy. Juice used as anti-leech, besides its religious values (Sikkim: [79]). Used as deobstruent, antispasmodic, obstructed menses and hysteria (Sikkim: [89]). Crushed and extract applied externally on skin to treat itching in cattle; fresh leaves grounded, sap extracted and used as nasal drop to stop nose bleeding in cattle (Sikkim: [84]).
101	*Aster neo-elegans* Grierson	Shrub	Asteraceae			**Whole plant:** Used as medicine (KL Bhutan: [71]).
102	*Aucklandia costus* Falconer	Herb	Asteraceae	Kapisful, Kuth (Np)		**Plant:** Believed to cure bronchitis, vomiting, epilepsy, headache, and hysteria (Sikkim: Database).
103	*Bidens pilosa* L.	Herb	Asteraceae	Kuro (Li)	500-2500	**Leaves:** Juice applied to eyes and ears to reduce pain (Sikkim: [79]). **Leaves and roots:** Extract used in cut and jaundice (KL Nepal: [53]).
104	*Bidens* spp.	Herb	Asteraceae	Zumphirobu (Sh)		Edible (KL Bhutan: [70]).
105	*Blumea hieraciifolia* (D. Don) DC.	Herb	Asteraceae	Sahasrabooti (Np)	900-1800	**Leaves:** Dried and taken smell to treat asthma (Darjeeling: [74]).
106	*Blumea lacera* (Burf. f.) DC.	Herb	Asteraceae	Gangansu (Me); Rando, Ghar nagharni (S)	150-350	**Roots:** Paste sticked on and around swelling region to prevent cutaneous infection (Jhapa: [68]). Decoction given in urinary infections, and also with decoction of *Plumeria acuta* given in gonorrhoea and spermatorrhoea (Jhapa: [66]).
107	*Calendula officinalis* L.	Herb	Asteraceae		2600-4400	**Leaves and flowers:** Used as antiseptic, antifungal, diaphoretic, stimulant, antispasmodic and in small pox; also used in healing wounds, ulcers, and burns (Sikkim: Database).
108	*Chrysanthemum indicum* L.		Asteraceae	Godawari (Li)	100-2900	**Flowers:** Dried flowers chewed during stomachache (Sikkim: [79]).
109	*Cirsium* sp.	Herb	Asteraceae			**Whole plant/flowers:** Used as medicine (KL Bhutan: [71]).
110	*Cremanthodium humile* Maxim.	Herb	Asteraceae			**Whole plant/flowers:** Used as medicine (KL Bhutan: [71]).
111	*Eclipta prostrata* (L.) L.	Herb	Asteraceae	Bhringaraj (Np); Khetkeshari (S)	200-1200	**Roots:** Used in treatment of snake and scorpion bite (Jhapa: [66]). **Leaves and tender shoots:** Used to treat cut and wounds and jaundice (Panchthar: [67]). **Leaves:** Infusion used in catarrhal (Jhapa: [66]).
112	*Elephantopus scaber* L.	Herb	Asteraceae	Sahsra Jari (Np); Dadari (Me)	200-1500	**Roots:** Paste applied on the muscular pain (Jhapa**:** [68]). **Fruits:** Used as tonic (Jhapa: [66]).
113	*Erigeron multiradiatus* (Lindl. ex DC.) Benth. ex C.B. Clarke	Herb	Asteraceae			**Flowers:** Used as medicine (KL Bhutan: [71]).
114	*Eupatorium adenophorum* Spreng.	Herb	Asteraceae	Banmara (Np)	850-2500	**Plant:** Juice applied on fresh cut (Sikkim: [75]; Ilam: [73]) and also taken in fever (Ilam: [73]).
115	*Eupatorium cannabinum* L.	Herb	Asteraceae	Banmara (Np)	1000-2000	**Stem and Leaves:** Extract used on cut and bruises to stop bleeding and infection (Darjeeling: [80]; Sikkim: [11], Database).
116	*Eupatorium odoratum* L.	Herb	Asteraceae	Aule banmara (Np); Daubanthu (Me)	400-1500	**Leaves:** Juice applied on cut and injury as haemostatic and to check nasal bleeding, extract dropped in nose to cure severe headache (Jhapa: [68]). Extract also used in cuts and wounds (Sikkim: Database).
117	*Gnaphalium affine* D. Don	Herb	Asteraceae	Pahelo Bukey (Np)	600-3700	**Plant:** Whole plant crushed and given orally to infants suffering from diarrhoea (Darjeeling: [52]).
118	*Grangea maderaspatana* (L.) Poir.	Herb	Asteraceae	Chot Bhidimyan (S)	150	**Aerial parts:** Pounded together with *Sphaeranthus indicus* and taken orally as well as inhaled a few drops to restore consciousness during epileptic fit (Jhapa: [66]).
119	*Inula cappa* (Buch.-Ham. ex D.Don) DC.	Shrub	Asteraceae	Golden Samphire, Sheep's Year (Eg); Bakhrikane, Gaitihare, Kanpate, Tihare- Phul (Np); Basita, Machram (Ri).	1000-2500	**Roots:** Juice used in fever, indigestion, and other stomach disorders (Taplejung: [81]).
120	*Inula helenium* L.	Herb	Asteraceae			**Roots:** Used as medicine (KL Bhutan: [71]).
121	*Ixeridium gracile* (DC.) Pak & Kawano	Herb	Asteraceae			**Whole plant:** Used as medicine (KL Bhutan: [71]).
122	*Leontopodium jacotianum* Beauverd	Herb	Asteraceae	Bhuke Phul, Jhulo (Np-Dl); Tawa Thokar, Tawa Thokar Yungpa (Tb)	2700-4900	**Plant:** Used as incense (Taplejung: [81]).
123	*Leontopodium monocephalum* Edgew.	Herb	Asteraceae	Bhuke Phul, Jhulo (Np-Dl); Tawa Thokar goepa (Tb).	4600-5600	**Plant:** Used as incense (Taplejung: [81]).
124	*Leontopodium* sp.	Herb	Asteraceae			**Rhizomes:** Used as medicinal (KL Bhutan: [71]).
125	Lorentea sp.	Herb	Asteraceae	Khainingroo or Rumplung (Sh)		**Leaves:** Edible (KL Bhutan: [70]).
126	*Pulicaria insignis* Drumm. ex Dunn	Herb	Asteraceae			**Whole plant/flowers:**Used as medicinal (KL Bhutan: [71]).
127	*Pulicaria* sp.	Herb	Asteraceae			**Flowers:** Used as medicinal (KL Bhutan: [71]).
128	*Saussurea costus* (Falc.) Lipsch.	Herb	Asteraceae			**Roots:** Used as medicine (KL Bhutan: [71]).
129	*Saussurea gossypiphora* D.Don	Herb	Asteraceae	Bhutkesh, Kapase Phool (Np-Tp); Yazembawa (Wl)	3500-5700	**Whole plant:** Used as medicinal (KL Bhutan: [71]). **Fibres:** Used for various purposes (Taplejung: [81]).
130	*Senecio cappa* Buch.-Ham. ex D. Don	Herb	Asteraceae	Bakhrakane (Np)		**Roots and leaves:** Infusion used in fever and boils (Ilam: [73, 76]).
131	*Senecio chrysanthemoides* DC.	Herb	Asteraceae			**Whole plant:** Used as medicinal (KL Bhutan: [71]).
132	*Sonchus arvensis* L.	Herb	Asteraceae	Ban-rayo (Np)		**Roots:** Paste applied to reliefe from toothache (Darjeeling: [74]).
133	*Sonchus wightianus* DC.	Herb	Asteraceae			**Roots:** Taken in jaundice (Sikkim: [75]).
134	*Soroseris hookeriana* (C.B.Clarke) Stebbins	Herb	Asteraceae			**Whole plant:** Used as medicine (KL Bhutan: [71]).
135	*Sphaeranthus indicus* L.	Herb	Asteraceae	Bad Bhidimyan (S)	200-800	**Shoots:** Paste applied on breast of women to cure swelling and wounds (Jhapa: [66]).
136	*Tagetes erecta* L.	Herb	Asteraceae	Demal-bhiwar (Me)	1800-2000	**Leaves:** Juice drunk to cure pneumania and chest pain (Jhapa: [68]). **Flowers:** Useful in pneumonia, piles, and jaundice (Panchthar: [67]).
137	*Tagetes patula* L.	Herb	Asteraceae	Sayapatri (Np)	900-2000	**Flowers:** Chewed to cure sore throat, cough and mouth ulcer (Sikkim: [79]).
138	*Tanacetu matkinsonii* (C.B.Clarke) Kitam.	Herb	Asteraceae			**Whole plant:** Used as medicinal (KL Bhutan: [71]).
139	*Tanacetum tatsienense* (Bureau & Franch.) K. Bremer & Humphries	Herb	Asteraceae			**Whole plant/flowers:**Used as medicinal (KL Bhutan: [71]).
140	*Taraxacum officinale* F.H. Wigg.	Herb	Asteraceae			**Whole plant:** Used as medicinal (KL Bhutan: [71]).
141	*Taraxacum sikkimense* Hand.- Mazz.	Herb	Asteraceae	Wakhur (Sh); Tuki Phool (Np); Khurmang, Wakhur (Tb)	3600-4700	**Plant:** Used as vegetable. **Flowers and leaves:** Used as galactagogue for human and cattle (Taplejung: [87]).
142	*Taraxacum tibetanum* Hand.-Mazz.	Herb	Asteraceae	Khenpa-karpo (Dz/Sh/B)		**Whole plant:** Used as medicinal (KL Bhutan: [71]).
143	*Vernonia anthelmintica* (L.) Willd.	Herb	Asteraceae	Sauraj (S)	1200-2000	**Fruits:** Pounded and taken to remove intestinal worms and to cure stomachache (Jhapa: [66]).
144	*Vernonia cinerea* (L.) Less.	Herb	Asteraceae	Jurishuri (S)	100-2300	**Leaves:** Decoction given in fever (Jhapa: [66]).
145	*Waldheimia glabra* (Decne.) Regel	Herb	Asteraceae	Gang Poe (Tb), Ghanga-Setik (Wl).	4100-5400	**Plant:** Used as incense (Taplejung: [87]).
146	*Impatiens balsamina* L.	Herb	Balsaminaceae	Tiuri (Np)	1200-1900	**Plant:** Decoction used to cure burns and urinary problems (Ilam: [73, 76]).
147	*Basella alba* L.	Climber	Basellaceae	Purne arak (S); Poi sag (Np)	200	**Young shoots:** Used as vegetable (Jhapa: [92]).
148	*Begonia picta* Sm.	Herb	Begoniaceae	Begonia (Eg); Magar Kanche (Np); Shovaparnee (Sn)	600-2800	**Plant:** Juice taken in headache and conjunctivitis (Ilam: [73, 76]). **Stalks:** Extracts from stalks used for venereal disease (Sikkim: Database). **Fruit:** Juice applied as an anti-leech agent (Taplejung: [88]). **Shoots and leaves:** Used to make pickle and jam (Ilam: [94]).
149	*Berberis angulosa* Wall. ex Hook. f. & Thomson	Shrub	Berberidaceae	Chutro (Np)	3400-4500	**Stem:** Decoction taken orally in blood dysentery and jaundice (Darjeeling: [52]).
150	*Berberis aristata* DC.	Shrub	Berberidaceae	Berberry (Eg); Karya (Gr-Mn); Kyarbukung (Lp); Chutro, Musa Lede (Np); Chotto (Np-Dl); Kyerwa, Kyerkar (Km, Tb); Chompairaim (Ri); Daruharidra, Rasanjan (Sn); Kerpatsang (Dz)	1800-3500	**Roots and bark:** Used as medicine (KL Bhutan: [71]).Used in jaundice, malaria, fever, and diarrhea; also used externally to cure eye disease (Sikkim: Database). **Leaves, flowers and bark:** Used in eye disease, bile disorders, lympy disorder, jaundice, malarian fever, swelling, and dysentery (Ilam: [73]; Panchthar: [67]). **Stem:** Extract used for hypoglycemic activities (Sikkim: [96]). **Fruit:** Eaten raw (Ilam: [73]; Panchthar: [67]), also used to cure rabies (Sikkim: [75]).
151	*Berberis asiatica* Roxb. ex DC	Shrub	Berberidaceae	Berberry (Eg); Toksong, Pirima (Li); Chutro, Musa Lede (Np); Chotto (Np-Dl); Daruharidra, Thakti-Layem (Ri)	1200-2500	**Bark:** Decoction used to treat conjunctivitis, eye inflammation, and also used as laxative and tonic (KL Nepal: [83]). **Roots**: Source of dye (KL Nepal: [83]).Bark and root decoction administered orally in jaundice and fever (Sikkim: [79]). **Fruit:** Eaten raw (Taplejung: [82, 87]). **Fruit and leaves**: Juice taken in diarrhoea and dysentery (Sikkim: [79]).
152	*Berberis nepalensis* Spreng.	Shrub	Berberidaceae			**Bark:** Use to extract dye (KL Bhutan: [70]).
153	*Mahonia acanthifolia* D.Don	Shrub	Berberidaceae	Chutro (Np)		**Stem:** Decoction taken to treat blood dysentery, diarrhoea and jaundice (Darjeeling: [52]).
154	*Mahonia napaulensis* DC.	Shrub	Berberidaceae	Mahonia (Eg); Samlikhe, Samjikhe (Li); Chutro, Jamane Mandro (Np); Khlusa (Ri); Daruharidra, Kanchan (Sn); Kerbe (Tm); Kerpa (WI)	1400-2900	**Plant:** Used for fencing (Taplejung: [81, 82]). **Fruit**: Used in the treament of urinary disorders (Taplejung: [81, 82]). Ripe berries eaten raw (Taplejung: [81]). **Bark:** Juice applied in eyes (Sikkim: [79]). **Fruit and bark:** Decoction taken to treat dysentery, diarrhoea (Ilam: [73, 76]; Sikkim: [86]), and urinary disorders (Ilam: [73, 76]).
155	*Sinopodophyllum hexandrum (Royle) T.S.Ying	Herb	Berberidaceae	Himalayan May Apple (Eg); Balulu, Balugu (Km); Laghu Patgra (Np); Meme Gudruk (Np-DI); Upala, Bamasisi, Ramasisi (Sh, WI); Wolmose (Tb); Goegabetapi (Sh)	2400-4500	**Plant:** Useful for typhoid fever, mental disorder, and plague (Sikkim: Database). Used as medicine (KL Bhutan: [71]). **Roots:** Used as purgative, hepatic stimulant, bile expellant, bitter tonic and in skin diseses (Sikkim: [69]). Decoction used in ulcer and liver troubles (Taplejung: [81]). Crushed and applied externally on hoof to treat infection; decoction used for cattle to treat indigestion (Sikkim: [84]). **Roots and fruit:** Used as anticancer remedy (Sikkim: [75]), taken in fever and diarrhea (Taplejung: [81]; Sikkim: [75], Database). **Fruit:** Ripe fruits eaten raw; used in gynecological diseases, menstrual disorders, kidney disease, skin disease, and cough (Taplejung: [81]).
156	*Betula alnoides* Buch.-Ham. ex D. Don	Tree	Betulaceae	Saur (Np)	1200-2600	**Bark:** Paste applied on snake bite (Sikkim: [75]). Chewed as a substitute of betel nut (Ilam: [94]).
157	*Betula cylindrostachya* Lindley	Tree	Betulaceae	Saru (Np); Taghyam (Bh)	1400-2800	**Leaves:** Buds used as substitute for tea leaves (Sikkim: [72]).
158	*Betula utilis* D. Don	Tree	Betulaceae	Himalayan Silver Birch (Eg); Bhojpatra, Bhujpata, Bhojpatra (Np); Bhuj, Bhujpat (Np-Dl); Tag-Pa Tak-Pa (Km, Sh, Tb, Wl); Bhurjha, Bhurjapatra (Sn).	2700-4300	**Branch:** Used during marriage ceremony (Taplejung: [81]). **Bark:** Crushed and applied on injuries of cattle (Sikkim: [97]). Boiled and used for cleaning wounds as antiseptic (Sikkim: [79]).
159	*Oroxylum indicum* (L.) Kurz	Tree	Bignoniaceae	Totala, Tatelo (Np); Totalabimfang (Me)	400-1400	Plant useful in jaundice (Darjeeling: [105]). **Roots, bark and fruit:** Used in fever, bronchitis, dysentery, and asthma (Sikkim: [85]). **Root bark**: Improves appetite, taken in vomiting, asthma, and bronchitis (Darjeeling: [80, 89]). **Bark and seeds:** Powder used to treat dropsy, sprains, asthma, urinary disorders (Ilam: [73, 76, 77]), high fever and pneumonia (Sikkim: [11]). **Seeds:** Used as medicinal (KL Bhutan: [71]). Endosperms eaten to cure pneumonia (Jhapa: [68]). **Bark**: Powder applied on chronic wounds (Jhapa: [68]) and also used to treat burns, boils (Panchthar: [67]), and diarrhea (Darjeeling: [86]; Panchthar: [67]). **Flowers:**Usedas medicinal (KL Bhutan: [71]). Edible (Darjeeling: [80]). **Flowers:** Edible (Darjeeling: [80]; Ilam: [94]; Sikkim: [89]).
160	*Stereospermum chelonoides* (L. f.) DC.	Tree	Bignoniaceae	Pader (S)	150-250	**Fruit:** Tied as an amulet to cure migrain (Jhapa: [66]).
161	*Bombax ceiba* L.	Tree	Bombacaceae	Edel (S); Simal (Np); Pemgeyser (Sh)	500-1500	**Roots and bark:** Used as emetic (Panchthar: [67]) and also used to treat diarrhea and dysentery (Darjeeling: [86]; Panchthar: [67]; Sikkim: Database). **Roots**: Decoction given in urinary infection, also with decoction of *Plumaria acuta* given in gonorrhoea and spermatorrhoea (Jhapa: [66]). **Flowers:** Used as medicine (KL Bhutan: [71]). Buds cooked as vegetable (Jhapa: [92]). Pickled and eaten twice daily to get relief from diarrhea and dysentery (Jhapa: [68]). Paste applied externally on small pox in children (Sikkim: [79]). Exude used as gum (KL Bhutan: [70]).
162	*Heliotropium indicum* L.	Herb	Boraginaceae	Hatisude (S)	100	**Young stem:** Used with bulb of onion to cure rabies (Jhapa: [66]).
163	*Onosma hookeri* C. B. Clarke	Herb	Boraginaceae	Laljari (Np); Bemu (Bhu); Muktsi (Sh)	3000-4700	**Roots:** Oil used externally as hair tonic (Sikkim: [49]). Used as medicinal (KL Bhutan: [71]).
164	*Brassica campestris* L. var cumifolia Roxb.	Herb	Brassicaceae	Rayo (Np)	2500	**Whole plant:** Used to make fermented material called 'Gundruk' (Darjeeling: [108]; Sikkim: [101]). Used in fever, indigestion and irritation (Ilam: [76]).
165	*Brassica* sp.	Herb	Brassicaceae	Yoongkar (Dz)		**Seed:** Used as medicine (KL Bhutan: [71]).
166	*Capsella bursa-pastoris* (L.) Medik.	Herb	Brassicaceae	Shepherd's Pursa (Eg); Chamsure Jhar, Tori Ghans, Tori Jhar (Np); Chhyamachhyaru (Sh, Wl)	1400-4500	**Plant:** Used as green vegetable (Taplejung: [87]). Used as medicine (KL Bhutan: [71]). **Leaves:** Juice used in malarian fever (Darjeeling: [78]).
167	*Cardamine hirsuta* L.	Herb	Brassicaceae	Simrayo (Np)	500-3000	**Shoot:** Extract taken to low blood pressure and in cardiac problems (Darjeeling: [74]; Sikkim: [75]).
168	*Cardamine macrophylla* Willd.	Herb	Brassicaceae	Chhurukpa (Sh, Wl)	2500-4500	**Plant:** Used as vegetable (Sikkim: [98]; Taplejung: [87])and also made fermented vegetables (North-East India: [101]).
169	*Erysimum hieraciifolium* L. f.	Herb	Brassicaceae	Chhasey (Dz); Kharshing (Sh); Phaledo (Np)		**Seed:** Used as medicinal (KL Bhutan: [71]).
170	*Lepidium sativum* L.	Herb	Brassicaceae	Chamsur (Np)	200-3000	**Plant:** Consumed as vegetable; useful in piles, asthma, cough, syphilis and bodyache (Ilam: [76]).
171	*Malcolmia* sp.	Herb	Brassicaceae			**Whole plant:**Used as medicine (KL Bhutan: [71]).
172	*Nasturtium officinale* R. Brown	Herb	Brassicaceae			**Aerial parts:** Decoction given to relieve body pain; young shoots taken as salad (Ilam: [76, 94]; Sikkim: [11, 79]). **Plant:** Juice given in indigestion and urinary disorder (Ilam: [76]).
173	*Raphanus sativus* L.	Herb	Brassicaceae	Mula (Np)		**Whole plant:** Used to make fermented material called 'Sinki' and 'Gundruk' (Darjeeling: [101, 108]). Used in indigestion, liver and gall bladder troubles, urinary complaints and ear pain (Ilam: [73, 76]).
174	*Thlaspi arvense* L.	Herb	Brassicaceae	Jay-kha (Dz)		**Whole plant:** Used as medicinal (KL Bhutan: [71]).
175	*Canarium sikkimense* King	Tree	Burseraceae	Poskar shing (Sh)		Exude or resin used as medicine (KL Bhutan: [71]). Used as incense (KL Bhutan: [70]).
176	*Garuga pinnata* Roxb.	Tree	Burseraceae	Aule Dabdabe, Dubdabey (Np)	300-1200	**Bark:** Root bark used for curing skin disease; juice applied to treat dislocated bones and to heal wounds (Sikkim: [85]). **Fruit:** Taken to improve digestion (Sikkim: [85])
177	*Codonopsis foetens* Hook.f. & Thomson	Climber	Campanulaceae	Gaytangru (Sh)		**Whole plant:** Used as medicine (KL Bhutan: [71]).
178	*Lobelia angulata* G. Forst.	Herb	Campanulaceae			**Plant:** Decoction given to treat throat pain and fever. **Tender shoots:** Juice applied externally to treat boils and inflammation (Sikkim: [11]).
179	*Lobelia pyramidalis* Wall.	Herb	Campanulaceae	Eklebir (Np)		**Leaves and flowers:** Used as antispasmodic (Sikkim: [75]).
180	*Pratia nummularia* (Lam.) A. Br. & Asch.	Herb	Campanulaceae	Lanka Sanay (Np)	1000-2400	**Leaves:** Juice taken orally in dysentery and tonsillitis (Darjeeling: [74]).
181	*Cannabis sativa* L.	Herb	Cannabaceae	Gaja (Np); Gaja (S)		**Leaves:** Infusion taken to cure stomach pain and flatulence (Jhapa: [66]). **Stem:** Cut into small pieces and fed to livestock to treat inflammation; small pieces mixed with fodder to feed cattle as a tonic (Sikkim: [84]). Decoction given orally to treat severe diarrhoea (Sikkim: [75, 79]). **Flowers:** Dried flower paste taken in empty stomach to treat diarrhoea (Darjeeling: [86]). **Seeds:** Pounded and taken to relieve body pain (Sikkim: [75, 79]).
182	*Canna indica* L.	Herb	Cannaceae		900	**Rhizomes:** Edible and taken to treat fever (Sikkim: [11]). Extraction given to cure urinary troubles (Jhapa: [66]).
183	*Capparis zeylanica* L.	Shrub	Capparaceae	Asaria (S)	150	**Fruits:** Edible (Jhapa: [92]).
184	*Dipsacus atratus* Hook.f. & Thomson ex C.B.Clarke	Herb	Caprifoliaceae	Yika (Dz), Pinsa (Sh),		**Whole plant:** Used as medicine (KL Bhutan: [71]).
185	*Pterocephalus hookeri* (C.B.Clarke) E.Pritz.	Herb	Caprifoliaceae			**Flowers/whole plant:** Used as medicine (KL Bhutan: [71]).
186	*Viburnum cylindricum* Buch.-Ham. ex D. Don	Shrub	Caprifoliaceae	Arrow wood (Eg); Hanggase (Li); Gharaghuri, Ghar ghure, Ghode khari (Np)	1000-2500	**Seeds:** Oil used to treat burns (Panchthar: [67]) and also used for cooking purpose (Taplejung: [87]).
187	*Carica papaya* L.	Herb	Caricaceae	Mewa (Np)	100-1000	**Latex:** Mixed with salt and applied to cure ringworm (Jhapa: [66]). Fruits: Eaten raw and also eaten to cure jaundice (Sikkim: [79]).
188	*Arenaria densissima* Wall. ex Edgew. & Hook.f.	Herb	Caryophyllaceae			**Whole plant:** Used as medicine (KL Bhutan: [71]).
189	*Drymaria cordata* (L.) Willd. ex. Roem. & Schult.	Herb	Caryophyllaceae	Abhijalo (Np); Hachiya-gara-gamso (Me)	2200-4300	**Plant:** Burned and inhaled for antipyretic effect (Darjeeling: [78]). Paste useful to treat fever, cold and cough (Darjeeling: [78]) also used for dog bites (Sikkim: Database), headache and sore throat (Sikkim: [11]). Paste applied externally on fractured bone and bandaged with the help of cotton cloth; decoction administered to animal to treat mouth ulcer (Sikkim: [84]). **Above ground parts**: Steamed and smelled during sinus trouble (Darjeeling: [52, 78, 80]; Sikkim: [11]; Panchthar: [67]). **Leaves:** Pasted with *Urena lobata* applied for cutaneous infections (Jhapa: [68]). Useful in diarrhea and dysentery (Panchthar: [67]).
190	*Drymaria diandra* Blume	Herb	Caryophyllaceae	Avijalo (Np)	700-2000	**Plant:** Juice useful in cough, cold and sinusitis (Ilam: [73, 76]) and peptic ulcer (Ilam: [76]).
191	*Drymaria villosa* Cham. & Schlecht.	Herb	Caryophyllaceae	Abijalo (Np)		**Shoots:** Given to treat pneumonia and sinusitis (Sikkim: [75]).
192	*Celastrus paniculatus* Willd.	Shrub	Celastraceae	Kujur (S)	150-300	**Plant:** Juice taken in fever (Darjeeling: [78]). Fresh juice cures sores of throat (Darjeeling: [80]; Sikkim: [11]) and lungs (Jhapa: [66]). B**ark and oil**: Applied externally to treat acute stomach pain (Jhapa: [66]). **Shoots:** Juice taken to treat gastritis and constipation (Jhapa: [66]). **Leaves:** Juice used as eye drops to cure eye infection. Paste reduces swelling and applied on wounds to heal (Jhapa: [66]). Given to cattle to treat loss of appetite (Sikkim: [84]). **Seeds:** Paste applied on the skin allergies and good for gout (Sikkim: [11]).
193	*Chenopodium album* L	Herb	Chenopodiaceae	Bethu Saag (Np)		**Plant:** Used as appetizer, laxative, and diuretic; also useful in treatment of eye diseases, throat troubles, piles, blood heart, and spleen diseases (Sikkim: Database). Cooked and eaten as vegetable to reduce bodypain especially back pain (Sikkim: [79]). **Young shoots**: Consumed as vegetable (Ilam: [94]).
194	*Cleome gynandra* L.	Herb	Cleomaceae	Junge Phool (Np); Seta kata arak (S)	300	**Leaves:** Eaten as vegetable (Jhapa: [92]).
195	*Garcinia cowa* Roxb.	Tree	Clusiaceae	Egg tree (Eg); Kaphal (Np)	100-1300	**Fruit:** Sun dried and taken to treat dysentery (Sikkim: [85])
196	*Hypericum uralum* Buch.-Ham. ex D. Don	Herb	Clusiaceae	Urillo (Np)	1200-3600	**Bark:** Juice applied on wound and bruises (Sikkim: [75]). **Seeds:** Aromatic and stimulant (Sikkim: Database).
197	*Mesua ferrea* L., Tree		Clusiaceae	Nageeswari (Np)	400-950	**Bark:** Orally administered in various skin diseases (mostly poxes) and in menstrual disorder (Sikkim: Database). Bark or stem paste applied or taken orally in hydrocele and on wound (Sikkim: [53]).
198	*Terminalia bellirica* (Gaertn.) Roxb.	Tree	Combretaceae	Bhaayure (Me); Barro (Np); Lopong (S); Baru (Dz/Sh/T/B)	300-1100	**Fruit:** Used as medicine (KL Bhutan: [71]) and incense (KL Bhutan: [70]). Powder drunk to treat constipation (Jhapa: [68]). Useful in bronchitis (Jhapa: [66]; Sikkim: Database), asthma and respiratory trouble.Decoctionadministered to livestock for treatment of diarrhea and dysentery (Sikkim: [84]). **Bark:** Used for anemia and leucoderma (Sikkim: Database).
199	*Terminalia chebula* Retz.	Tree	Combretaceae	Silikhaa (Me); Aaru (Dz/Sh)	150-1100	**Fruits:** Edible (Sikkim: [47]). Used as medicine (KL Bhutan: [71]) and incense (KL Bhutan: [70]). Used as tonic and also to cure eye, heart and bladder diseases (Sikkim: Database). Powder taken to recover from gastric (Jhapa: [68]). Consumed during cough and sore throat and mouth ulcers (Sikkim: [79]). Powder of dried fruits and bark given in diarrhoea (Darjeeling: [86]). Fruit and/or bark decoction administered with small amounts of rock salt to cattle to treat diarrhea and dysentery (Sikkim: [84]).
200	*Terminalia myriocarpa* Van Heurck & Mull. Arg.	Tree	Combretaceae	Pani saaj (Np)	100-1000	**Bark:** Juice applied externally on cuts and wounds (Sikkim: [79]).
201	*Terminalia tomentosa* Wight & Arn.	Tree	Combretaceae			**Bark:** Use to extract dye (KL Bhutan: [70]).
202	*Commelina benghalensis* L.	Herb	Commelinaceae	Kane Jhar (Np)	900-1800	**Leaves:** Juice applied to treat conjunctivitis (Darjeeling: [74]).
203	*Commelina paludosa* Blume.	Herb	Commelinaceae	Bhaisen Jhar (Np)	300-3500	**Root:** Paste applied on boils (Darjeeling: [52]).
204	*Streptolirion volubile* Edgew.	Herb	Commelinaceae		1500-2400	**Leaves:** Crushed and applied on wounds of ear, nose and navel (Darjeeling: [52]).
205	*Cuscuta reflexa* Roxb.	Climber	Convolvulaceae	Dodder (Eg); Akash Beli, Akashveli, Amarbel, Indrabeli (Np); Chamjakhikwa, Jalisang (Ri); Alakjadi (S)	200-3100	**Plant:** Decoction used to treat jaundice (Taplejung: [88]; Panchthar: [67]; Sikkim: [75]; Ilam: [73, 76]). Infusion taken in diarrhea, bronchitis (Panchthar: [67]; Ilam: [73, 76]), and also applied externally to treat body ache and skin infections (Jhapa: [66]). **Shoots and seeds:** Used to cure cough (Sikkim: [75]).
206	*Cuscuta sinensis* Lam.	Climber	Convolvulaceae	Bayding/dukpoo-ru (Sh)		**Whole plant:** Used as medicine (KL Bhutan: [71]).
207	*Evolvulus alsinoides* L.	Herb	Convolvulaceae	Sankha Pushpi (Np)	550-1100	**Plant:** Used as febrifuge and aphrodisiac (Panchthar: [67]).
208	*Evolvulus nummularius* (L.) L.	Herb	Convolvulaceae	Chhatbatiza (S)	150-910	**Plant:** Applied on scorpion sting, cut, wounds and burns (Jhapa: [66]).
209	*Ipomoea aquatica* Forssk.	Climber	Convolvulaceae	Karmi (S); Lahore pani sag (Np)	300-1500	**Leaves and twigs:** Eaten as vegetable (Jhapa: [92]). **Stem:** Necklace prepared and put round the neck of jaundice patient (Darjeeling: [105]).
210	*Maianthemum purpureum* (Wall.) LaFrankie	Herb	Convolvulaceae	Khhiringlo, Khirro, Sikari-Sag (Np); Lekh Daro (Np-Dl)	2600-4300	**Young leaves and tender shoots:** Cooked as vegetables (Taplejung: [88]).
211	*Merremia umbellata* subsp. *orientalis* (Hallier f.) Ooststr.	Herb	Convolvulaceae		300-480	**Stem:** Extraction taken to enhance lactation (Jhapa: [66]).
212	*Cornus capitata* Wall.	Tree	Cornaceae	PhastiINamimpluse (Sh), Poitsi (Dz)		**Fruits:** Edible (KL Bhutan: [70]).
213	*Cornus macrophylla* Wall.	Tree	Cornaceae	ChapoiIBaminpa (Sh), Boray poitsi (Sh), Poitsi (Dz)		**Fruits:** Edible (KL Bhutan: [70]).
214	*Griselinia lucida* (J.R.Forst. & G.Forst.) G.Forst.	Shrub	Cornaceae			**Fruits:** Used as medicine (KL Bhutan: [71]).
215	*Bryophyllum pinnatum* (Lam.) Oken	Herb	Crassulaceae	Patharkuchi (Np)		**Leaves:** Paste applied on wound, bruises, swelling and insect bites (Sikkim: [69]).
216	*Kalanchoe integra* (Medikus) Kuntze	Herb	Crassulaceae	Hatnokane (Np)		**Leaves:** Juice taken orally as purgative (Sikkim: [85]).
217	*Coccinia grandis* (L.) Voigt	Climber	Cucurbitaceae	Gol Kankri (Np); Tilkocha (S)	200-900	**Fruit:** Unripe fruits used as vegetable (Jhapa: [92]).
218	*Cucurbita pepo* L.	Climber	Cucurbitaceae	Pharsi (Li)		**Seeds:** Powdered and taken orally for its vermifuge potency in children (Sikkim: [79]). **Fruit**: Used as medicine (KL Bhutan: [71]).
219	*Diplocyclos palmatus* (L.) C. Jeffrey	Climber	Cucurbitaceae	Kabubotke (S)	200-1500	**Leaves:** Decoction used in eye infections (Jhapa: [66]).
220	*Herpetospermum pedunculosum* (Ser.) Baill.	Climber	Cucurbitaceae	Ban Karela (Np); Serkyi Metog (Tb); Mendok Sepu (Wi)	1500-3600	**Fruit:** Inner par used in stomachache and to treat bile diseases (Taplejung: [81]).
221	*Momordica charantia* L.	Climber	Cucurbitaceae	Tite Karela (Np, Li)	300-2100	**Fruit:** Juice taken as blood purifier and also helps to control diabetes (Ilam: [76]; Sikkim: [79, 96]) and treat opthalmia and bleeding (Ilam: [76]). Used as vegetable (Ilam: [76]). **Leaves and fruit**: Useful in gastric troubles (Darjeeling: [91]).
222	*Trichosanthes tricuspidata* Lour.	Climber	Cucurbitaceae	Indreni (Np)	1200-2300	**Roots and fruit:** Extract used to treat gonorrhea, asthma, earache, and hemicrania (Ilam: [73, 76, 77]). **Roots:** Used in lung diseases of cattle. **Fruit:** Taken to cure asthma (Sikkim: Database). **Leaves:** Tender shoots used for vegetable (Ilam: [94]).
223	*Daphniphyllum himalense* (Benth.) Mull. Arg.	Tree	Daphniphyllaceae	Chandan (Np)	1200-2500	**Plant:** Plant has religious and aesthetic value (Sikkim: [72]).
224	*Dillenia indica* L.	Tree	Dilleniaceae	Ramphal (Np)	150-250	**Fruit:** Juice with sugar used as cooling beverage in fever and cough (Sikkim: [11], Database). **Bark and leaves:** Taken to cure diarrhea and dysentery (Sikkim: [11], Database).
225	*Dillenia pentagyna* Roxb.	Climber	Dilleniaceae	Sahad (S); Tatar (Np)	150-1500	**Fruit:** Edible (Jhapa: [92]).
226	*Dioscorea alata* L.	Climber	Dioscoreaceae	Ghartarul (Np)	600-1200	**Roots:** Used in fever, rash and itch, constipation, intestinal worms, leprosy, piles, and gonorrhoea. (Sikkim: Database). Eaten raw to treat throat pain (Sikkim: [11]).
227	*Dioscorea bulbifera* L.	Climber	Dioscoreaceae	Kaching (Lp); Ban tarul, Gittha, Kukurtarul (Np); Bengo nari (S); Tshemakewa(Dz), Borang-Joktang/Fantang (Sh)	150-2100	**Whole plant:** Used as medicine (KL Bhutan: [71]). **Tubers:** Edible (KL Bhutan: [70]). Used as aphrodisiac, stomachic, appetizer (Sikkim: Database), tonic and to cure ulcer (Sikkim: [75]). Boiled and eaten after submerging them whole night in cold water (Jhapa: [92]; Darjeeling: [80]; Ilam: [94]; Sikkim: [89, 107], Database) and also used for washing clothes, to kill lice and fish and as contraceptive pills (Sikkim: Database).
228	*Dioscorea deltoidea Wall. ex Griseb.	Climber	Dioscoreaceae	Ban Tarul, Kukur Tarul (Np); Kamanduki Saplokha (Ri)	450-3100	**Tubers:** Cooked as vegetable; juice taken as oral contraceptives and also used in lice problems (Ilam: [76]; Taplejung: [82]).
229	*Dioscorea pentaphylla* L.	Climber	Dioscoreaceae	Kusok (Lp); Aser, Bantarul, Bhyakur (Np)	600-1500	**Tubers:** Boiled and eaten after submerging them whole night in cold water (Jhapa: [92]). Boiled and taken orally as anthelmintic and wormifuge especially against tapeworm (Darjeeling: [52]).**Tubers and shoots:** Used as tonic and can also cure swelling (Sikkim: [75]).
230	*Shorea robusta* Gaertn.	Tree	Dipterocarpaceae	Sal (Np); Sasing (Li)	150-1500	Used to extract oil (KL Bhutan: [91]). Exude used as incense (KL Bhutan: [70]). **Bark:** Paste applied on wound and bone fracture (KL Nepal: [53]).
231	*Drosera peltata* Thunb.	Herb	Droseraceae			**Whole plant:** Used as medicine (KL Bhutan: [71]).
232	*Diospyros lotus* L.	Tree	Ebenaceae	Gundum (Dz), Amdebu (Sh)		**Fruit:**Used as medicine (KL Bhutan: [71]).
233	*Diospyros montana* Roxb.	Tree	Ebenaceae	Gada tarul (S)	500	**Fruit:** Pulp applied on cracks of feet (Jhapa: [66]).
234	*Elaeagnus infundibularis* Momiy.	Shrub	Elaeagnaceae	Bastard-Oleaster (Eg); Pirima (Li); Guenlo, Maldhendo, Madilo (Np); Tikun (Tm)	1500-2500	**Fruit**: Used to make alcohol (Taplejung: [81]).
235	*Elaeagnus latifolia* L.	Tree	Elaeagnaceae		700-2300	**Fruit:** Edible (KL Bhutan: [70]; Sikkim: [47]).
236	*Elaeagnus rhamnoides* (L.) A. Nelson	Tree	Elaeagnaceae			**Fruit:** Used as medicine (KL Bhutan: [71]).
237	*Hippophae salicifolia* D.Don	Shrub	Elaeagnaceae	Seabuckthorn (Eg), Achuk, Dale Chuk (Np)	2200-3500	**Bark and fruit:** Useful in lung dieases, skin eruptions, and irritations (Sikkim: Database). **Fruit:** Edible (Sikkim: [95]; KL Nepal: [83]) and also used to make vinegar (KL Nepal: [83]). Used in toothache, joint pain, liver, lungs, and phlegm diseases, menstrual disorders, dysentery, gum infection, blood disorders, diabetes and intestinal parasities (KL Nepal: [83]). **Roots:** Fresh root nodules chewed to stop vomitting and also to remove bad smell of mouth (Darjeeling: [49]).
238	*Hippophae tibetana* Schlecht.	Shrub	Elaeagnaceae	Seabuckthorn (Eg); Bhui Chuk (Np)	3800-4500	**Fruit:** Edible and also used to obtain yellow dye (KL Nepal: [83]).
239	*Elaeocarpus sikkimensis* Masters	Tree	Elaeocarpaceae		1500-2100	**Fruit:** Edible (Ilam: [92]; Sikkim: [95]).
240	*Elaeocarpus sphaericus* (Gaertn.) K. Schum.	Tree	Elaeocarpaceae	Rudraksh (Np)	700-1700	**Fruit:** Used in *vata* and *kapha* disease of head and epileptic fits (Sikkim: Database). Edible (Sikkim: [89]). **Seed:** Paste administered to cure cough (Sikkim: [79]).
241	*Elaeocarpus varunua* Buch.-Ham. ex Mast.	Tree	Elaeocarpaceae	Gasha-thungsey (Sh)		**Fruit:** Edible (KL Bhutan: [70]).
242	*Agapetes serpens* (Wight) Sleumer	Shrub	Ericaceae	Bandare Khorsani (Np)	1500-2600	**Flowers:** Edible (Sikkim: [80, 89]).
243	*Gaultheria fragrantissima Wall.	Shrub	Ericaceae	Wintergreen (Eg); Singjang, Singjhangma (Li); Dhasingare, Patpate (Np); Limbuni Phool (Np-Tp); Lamchassi (Ri); Chhyaro (Sh,Wi)	1200-2600	**Leaves**: Used as antiseptic (Sikkim: [75]), fodder and in ritual ceremony; warm juice used to treat inflammation and swellings (Taplejung: [87]). **Leaves and fruit:** Decoction used to treat reheumatism (KL Nepal: [83]; Sikkim: [75]) and worms (KL Nepal: [83]; Ilam: [73]). **Fruit:** Ripe fruits are eaten raw (KL Nepal: [87]).
244	*Gaultheria procumbens* L.	Shrub	Ericaceae			**Leaves:** Used to extract oil (KL Bhutan: [93]).
245	*Gaultheria* sp.	Shrub	Ericaceae	Chanze kam (Dz); Shogshingma shing (Sh)		**Whole plant:** Used as incense (KL Bhutan: [70]).
246	*Gaultheria trichophylla* Royle	Shrub	Ericaceae	Ani Gnonzing (Sh, Wi), Sanchanchewa (Tm)	2700-4500	**Fruit:** Ripe fruits eaten raw (Taplejung: [81]; Darjeeling: [80]; Sikkim: [89]).
247	*Lyonia ovalifolia* (Wall.) Drude	Tree	Ericaceae	Lyonia (Eg); Tapeba (Li); Angeri (Np); Rangkhilayem (Ri) Dhobang (Sh); Sangemi Dongbu, Syanggomba (Wi).	1300-3300	**Leaves:** Dried and used as cigarette wrapper (Ilam: [81]). Infusion taken to treat scabies and dog bite (Ilam: [73, 76]).
248	*Pieris formosa* (Wall.) D.Don.	Tree	Ericaceae	Balu (Np); Kekphel (Li)	2000-3300	**Roots:** Dust applied to treat rheumatism (KL Nepal: [53]).
249	*Rhododendron anthopogon* D. Don	Shrub	Ericaceae	Fragrant Rhododendron (Eg); Sunpati (Np); Balu (Dz/B/Sh)	3500-5100	**Aerial parts:** Used as incense and snuffed to induce sneezing (Sikkim: Database). **Leaves and flowers:** Used for stomach, liver, and lung disorders, indigestion, sore throat, and as appetizer and in vomiting (Darjeeling: [98]; KL Nepal: [83]). Used as incense (Sikkim: Database). **Flowers:** Used as medicine (KL Bhutan: [71]). Used to cure blood dysentery (Sikkim: [75]). **Whole plant:** Used as incense (KL Bhutan: [70]).
250	*Rhododendron arboreum* Smith	Tree	Ericaceae	Porota (Gr); Tokphekalaphun, Thukphewa (Li); Gurans, Laliguras (Np); Dakbun, Tokse (Ri); Pullasa (Sn); Ladukpa, Sendok Dongbu (Wi).	1400-3600	**Flowers and young leaves:** Useful in dysentery (Darjeeling: [80]; Ilam: [76]; Sikkim: [11, 85]; KL Nepal: [83]), diarrhea and headache (Ilam: [76]; Sikkim: [85]). **Flowers:** Used to treat headache (Panchthar: [67]). Powder taken to stop bleeding in female. Flower petals clear throat choking due to fish or chicken bone (Darjeeling: [52]; Sikkim: [11]). Used to make alcohol; paste applied around eyes for good sight (Taplejung: [81]). Used to make local wine (Darjeeling: [108]; Ilam: [94]). Crushed with water and administered to livestock to treat diarrhea and dysentery (Sikkim: [97]).
251	*Rhododendron campanulatum* D. Don	Tree	Ericaceae	Syapu (Gr); Chimal, Nilo Chimal, Seti Chimal (Np); Kalma (Sh); Takma Singya (Tb); Khama, Saje Medok (Wl).	2800-4400	**Wood:** Dried and infusion taken in fever (Darjeeling: [78]). **Leaves**: Used to wrap tobacco (Taplejung: [81]). **Flowers:** Nectar edible (Taplejung: [81]).
252	*Rhododendron lepidotum* Wall. ex G. Don	Shrub	Ericaceae	Bhale sunpati (Np)	2100-4700	**Leaves and flowers:** Paste used for bile and lung disease, cold, and blood disorders (KL Nepal: [83]). **Leaves**: Used for incense (KL Nepal: [83]).
253	*Rhododendron setosum* D. Don	Shrub	Ericaceae	Sunpatay (Np); Sulo (Dz/Sh/B/T)	3700-5600	**Flowers:** Used as medicine (KL Bhutan: [71]). **Leaves:** Local Buddhist uses leaf as incense (Sikkim: [89]). **Whole plant:** Used as incense (KL Bhutan: [70]).
254	*Vaccinium gaultheriifolium* (Griff.) Hook. f. ex C. B. Clarke	Shrub	Ericaceae	Chyansi (Np-Tp); Khapusekma (Li)	1500-2300	**Fruit:** Eaten raw or pickled; juice used to treat diarrhea and dysentery (Taplejung: [81]).
255	*Antidesma acidum* Retz.	Shrub	Euphorbiaceae	Archal (Np)	150-1200	**Bark and leaves:** Used to treat cholera (Panchthar: [67]). **Fruit:** Consumed raw (Ilam: [94]).
256	*Baccaurea ramiflora* Lour.	Tree	Euphorbiaceae		250-1300	**Fruits:** Edible (Sikkim: [95]).
257	*Baliospermum montanum* (Willd.) Mull. Arg.	Shrub	Euphorbiaceae	Danti (S)	300-910	**Seeds:** Used in gastric disorders, gouts, and rheumatism (Jhapa: [66]).
258	*Bischofia javanica* Blume	Tree	Euphorbiaceae	Kainjal (Np)	150-1200	**Leaves:** Chewed to cure sore throat (Sikkim: [11]). **Bark:** Juice taken to cure diarrhea (Sikkim: [11]).
259	*Bridelia retusa* (L.) A. Juss.	Tree	Euphorbiaceae	Gayo (Li)	100-1400	**Bark:** Paste prepared from the bark of *Bridelia retusa* and *Schima wallichii*applied externally on cuts and wounds (Sikkim: [79]).
260	*Croton roxburghii* N. P. Balakr.	Tree	Euphorbiaceae	Guti (S)	300-750	**Bark:** Pounded and mixed with little amount of oil of *Varanus* sp. and massage on the body to relieve from measles, chicken pox, and boils (Jhapa: [66]). **Roots and bark**: Used as purgative (Jhapa: [66]).
261	*Euphorbia griffithii* Hook.f.	Herb	Euphorbiaceae			**Roots:** Used as medicine (KL Bhutan: [71]).
262	*Euphorbia hirta* L.	Herb	Euphorbiaceae	Aankle Jhar (Np); Gofatkhalakhachri (Me); Sangadare, pusitoa (S)	150-1500	**Plant:** Extraction given to lactating mother to increase the milk prodcution. **Root:** Given to stop vomitting (Jhapa: [66]). **Young shoots:** Used to treat excessive bleeding during menstruation and also in gonorrhea (Jhapa: [68]). **Latex**: Applied on pimples and old wounds (Jhapa: [68]) and also to treat warts and cuts (Darjeeling: [74]).
263	*Euphorbia pulcherrima* Willd. ex Klotzsch	Shrub	Euphorbiaceae		800-1200	**Latex:** Applied on toothache (Sikkim: [11]).
264	*Euphorbia royleana* Boissier	Shrub	Euphorbiaceae	Siundee (Np); Sijau (Me)	1100-1200	**Latex:** Applied to cure swelling of skin due to cutaneous and sub-cutaneous infection (Jhapa: [68]). Used to cure cuts and stop bleeding, to relieve from earache, cough, and asthma (Sikkim: Database).
265	*Euphorbia sieboldiana* C.Morren & Decne.	Herb	Euphorbiaceae			**Tuber/roots:** Used as medicine (KL Bhutan: [71]).
266	*Euphorbia sikkimensis* Boiss.	Herb	Euphorbiaceae			**Tubers:** Used as medicine (KL Bhutan: [71]).
267	*Glochidion lanceolarium* Voigt.	Shrub	Euphorbiaceae	Bangikath (Np)		**Bark:** Juice to taken in stomach complaints (Sikkim: [85]).
268	*Homonoia riparia* Lour.	Shrub	Euphorbiaceae	Khola ruis (Np); Mongthel-Kung (Lp)		**Roots:** Decoction taken as laxative (Sikkim: [85]).
269	*Jatropha curcas* L.	Shrub	Euphorbiaceae	Aanda (Me); Kaden, Hathi-kane, Sjaiwan (Np); Bhernada (S); Ngera-kharshing (Sh)	500-1200	**Latex:** Applied to treat toothache and swelling testicules (Jhapa: [68]). Used to stop bleeding from wounds; also applied to treat burns, eczema, ringworm (Sikkim: Database) and scabies (Jhapa: [66]). **Bark**: Chewed to cure mouth sores (Jhapa: [66]). **Seeds:** Used as medicine (KL Bhutan: [71]).
270	*Mallotus philippinensis* Muel. Arg.	Tree	Euphorbiaceae	Sindure (Np); Rora (S)		**Seeds:** Dried and powder applied on the wound of cattle (Sikkim: [97]). Given to pigs along-with food to kill intestinal worms, rushed and applied externally to cure wound, injuries and skin infection (Sikkim: [84]). **Fruit:** Powder of glandular hairs of fruits applied in sores and wounds (Jhapa: [66]). **Bark:** Used as medicine (KL Bhutan: [71]). **Flowers and fruit:** Use to extract dye (KL Bhutan: [70]).
271	*Ricinus communis* L.	Shrub	Euphorbiaceae	Aadi (Me); Iradam (S); Chamlingshing (Sh)	150-2400	**Roots:** Used for skin diseases. Tied as an amulet on neck of children to stop vomiting (Jhapa: [66]). **Leaves:** Juice used to cure headache, boils, and dysentery; paste used to cure jaundice (Sikkim: Database). Warmed with mustard oil and massaged on the body of post delivery women to cure body pain (Jhapa: [66]). **Seeds:** Used as medicine (KL Bhutan: [71]). Endosperm applied as cream on dryness of skin to cure cracking heels (Jhapa: [68]).
272	*Tragia involucrata* L.	Climber	Euphorbiaceae	Ban Sisnu (Np); Sangelsim (S)	400	**Leaves:** Paste applied on local swelling of hands and feet ([66]).
273	*Abrus precatorius* L.	Climber	Fabaceae	Lalgedi (Np); Karmet (S)	300-1100	**Roots and fruits**: Extract taken orally for abortion (KL Nepal: [53]) and also taken for tonsil and pneumonia (Sikkim: Database). **Roots:** Paste usedto treat urinary troubles and skin disease (Ilam: [76]) and also in cough, cold and menstrual troubles and also applied to cure wounds (Jhapa: [66]). Powder taken to treat urinary troubles and skin disease (Ilam: [76]). **Leaves:** Juice taken orally to relieve urinary complaints (Jhapa: [66]). **Fruit:** Chewed or fresh root juice administered orally during throat pain (Sikkim: [79]).
274	*Acacia catechu* (L.f.) Willd.	Tree	Fabaceae	Toeja (Dz); Jasenshing (Sh)		**Stem:** Heartwood extract used as medicinal (KL Bhutan: [70, 71]) and gum (KL Bhutan: [70]). **Stem and roots:** Use to extract dye (KL Bhutan: [70]).
275	*Acacia intsia* (L.) Willd.	Shrub	Fabaceae	Kondru (S)	900-1100	**Root:** Paste used in fever, cough and cold, and also applied against snake and scorpion sting (Jhapa: [66]). **Stem:** Paste with pepper taken orally to cure blood dysentery (Jhapa: [66]). **Leaves:** Paste applied on sores and itches (Jhapa: [66]).
276	*Acacia pennata* (L.) Willd.	Climber	Fabaceae	Arare (Li)	200-1100	**Leaves:** Chewed with sugar and cumin during bleeding gums; juice administered orally in indigestion in infants (Sikkim: [79]).
277	*Aeschynomene indica* L.	Herb	Fabaceae	Tal Khukuri (Np); Sola (S)	200-1300	**Plant:** Juice and cumin seeds used in reducing fever (Jhapa: [66]).
278	*Albizia julibrissin* Durazz.	Tree	Fabaceae	Padke Siris (Np)	1000-3000	**Bark:** Extract or paste used to treat dandruff (KL Nepal: [53]).
279	*Albizia lebbeck* (L.) Benth.	Tree	Fabaceae	Harasiris (Np)		**Leaves and flowers:** Used to cure boils, piles, and diarrhea (Sikkim: [75]).
280	*Albizia procera* (Roxb.) Benth.	Tree	Fabaceae	Seti Siris (Li)	300-1100	**Bark:** Crushed into paste and applied on forehead during fever (Sikkim: [79]).
281	*Astragalus yunnanensis* Franch.	Herb	Fabaceae			**Whole plant:** Used as medicine (KL Bhutan: [71]).
282	*Atylosia scarabaeoides* (L.) Benth.	Climber	Fabaceae	Jangali bhatmase jhar (Np); Birhorec (S)	400-1200	**Plant:** Paste orally administered to treat body swelling (Jhapa: [66]).
283	*Bauhinia purpurea* L.	Tree	Fabaceae	Tanki (Np)	300-1600	Plant used against animal bite;useful as maturant for boils and ebcesses (Sikkim: Database). **Roots:** Used as carminative (Sikkim: Database, [79]). Paste applied on boils (Sikkim: [79]). **Bark:** Used to control diarrhea (Sikkim: Database). **Flowers:** Used as laxative (Sikkim: Database). **Leaves:** Used as fodder (Ilam: [73]). **Shoots:** Used as vegetable (Ilam: [94])
284	*Bauhinia semla* Wunderlin	Tree	Fabaceae			**Exude:** Used as gum (KL Bhutan: [70]).
285	*Bauhinia vahlii* Wight & Arn.	Climber	Fabaceae	Makrik (Lp); Verla, Baro Lara, Bhorla (Np)	200-1300	**Bark:** Useful in skin disease (Sikkim: Database) and diarrhea (Sikkim: Database, [74]). **Leaves:** Used as demulcent (Sikkim: Database). Paste applied in factured bone (Sikkim: [97]). **Seeds:** Used as tonic and aphrodisiac, and also given to treat snake bite (Panchthar: [67]; Sikkim: Database). Roasted and consumed (Ilam: [92]). **Seeds and leaves**: Given in dysentery and used as laxative (Sikkim: [75]).
286	*Bauhinia variegata* L.	Tree	Fabaceae	Koiraalo, Koirala, Takki (Np)	150-1900	**Root:** Dried root and bark administered orally in diarrhea (Darjeeling: [86]). Decoction given to expel placenta of cattle (Sikkim: [97]). **Bark:** Juice taken as tonic in toothache (Sikkim: [11]). Paste taken to cure swelling, leprosy, cough, and menstrual disorder (Ilam: [73, 76]; Sikkim: [79]). **Flower:** Juice taken to cure dysentery, diarrhea, and stomach pain (Sikkim: Database). Buds taken for skin disease and ulcer, dried buds chewed to treat bleeding piles (Sikkim: [11]). Cooked as curry (Darjeeling: [80]; Ilam: [73, 76]; Sikkim: [89]). **Fruit:** Used for blood purification (Sikkim: Database).
287	*Butea minor* Buch.-Ham. ex Baker	Shrub	Fabaceae	Bhujetro (Np)	300-2000	**Seeds:** Used as anthelmintic (Panchthar: [67]).
288	*Butea monosperma* (Lam.) Kuntze	Shrub	Fabaceae	Palans (Np); Marup (S); Flamingo tree (Eng)	150-1200	**Fruits:** Used as medicine (KL Bhutan: [71]). **Roots:** Used in tuberculosis (Jhapa: [66]).
289	*Caesalpinia bonduc* (L.) Roxb.	Shrub	Fabaceae	Sugrong-bithai (Me)	400-500	**Seeds:** Fried to black with coconut oil, crushed and paste applied on scalp with the help of cock's feather for baldness (Jhapa: [68]).
290	*Cassia fistula* L.	Tree	Fabaceae	Raj Brichhya (Np); Mukhralaudhi (Me); Noormui Mirfu Baha (S); Dongkoshing (Sh)	150-1400	**Leaves:** Used for treating skin diseases, extraction taken orally taken to purify blood, and also used as laxative (Jhapa: [66]). **Fruits:** Used as medicine (KL Bhutan: [71]). Used as diuretic, purgative, and laxative (Panchthar: [67]). Used for asthma, diabetes, and eczema (Sikkim: Database). Paste used to treat the whopping cough (Jhapa: [68]).
291	*Cassia occidentalis* L.	Herb	Fabaceae	Thulo Tapre (Np)	200-1400	**Flowers and seeds:** Paste applied in minor skin infection and inflammation (Jhapa: [68]).
292	*Cassia sophera* L.	Shrub	Fabaceae	Tapre (Np); Chakora (S)	700-1000	**Bark and seeds:** Infusion given in diabetes (Jhapa: [66]).
293	*Cassia tora* L.	Herb	Fabaceae	Tapre (Np); Chakora, Bhede deren (S)	450-1300	Used as medicine (KL Bhutan: [71]). **Leaves:** Extraction applied on ringworm and itch (Jhapa: [66]).
294	*Crotalaria pallida* Aiton	Herb	Fabaceae	Chhinchhinne (Np)	200-1750	**Roots:** Juice drunk to cure body-swelling problems (Jhapa: [68]).
295	*Desmodium gangeticum* (L.) DC.	Shrub	Fabaceae		300-1000	**Roots:** Used in snake and scropion bite (Jhapa: [66]).
296	*Desmodium triflorum* (L.) DC	Herb	Fabaceae	Sano Chameli (Np)	600-2300	**Leaves:** Juice consumed to treat diarrhoea and dysentery (Darjeeling: [74]).
297	*Entada phaseoloides* (L.) Merr.	Climber	Fabaceae	Pangra (Np), Kolokpu-sae (Sh)	350-1600	**Seeds:** Used as astringent and emetic (Panchthar: [67]; Sikkim: Database) and also useful to treat dandruff (Panchthar: [67]). Used as medicine (KL Bhutan: [71]).
298	*Entada pursaetha* subsp. *sinohimalensis* Grierson & D. G. Long	Climber	Fabaceae		400-1500	**Bark:** Juice applied externally to cure skin disease (Sikkim: [11]). **Seeds:** Paste applied to treat mumps; powder acts as antidandruff agent (Sikkim: [11]).
299	*Erythrina arborescens* Roxb.	Tree	Fabaceae	Phaledo (Np); Gyesey Kung (L)	1500-3000	**Leaves and bark:** Curative efficacies for skin diseases (Sikkim: [75]). **Seed:** Used as medicine (KL Bhutan: [71]).
300	*Erythrina stricta* Roxb.	Tree	Fabaceae	Phaledo (Np); Chhasey (Dz); Kharshing (Sh)	1000-1600	**Bark and leaves:** Paste given in rheumatism, fever, asthma, and epilepsy (Ilam: [73]). **Seed:** Used as medicine (KL Bhutan: [71]).
304	*Erythrina suberosa* Roxb.	Tree	Fabaceae	Phaledo (Np); Buru marar (S)	900-1200	**Bark**: Grinded with bark of *Oroxylum indicum* and given as antidote (Jhapa: [66]).
302	*Flemingia macrophylla* (Willd.) Merr.	Shrub	Fabaceae	Barvasi (Np)	700-1700	**Plant:** Decoction given to cattle to cure blood dysentery (Sikkim: [97]).
303	*Glycyrrhiza glabra* L.	Herb	Fabaceae	Jethimadhu (Np)		**Roots:** Given in cough, fever, dysentery, and chronic hepatitis (Sikkim: Database).
304	*Indigofera* sp.	Tree	Fabaceae			**Leaves:** Use to extract dye (KL Bhutan: [70]).
305	*Macrotyloma uniflorum* (Lam.) Verdc.	Tree	Fabaceae	Gahat (Li)	450-2800	**Seeds:** Decoction used to remove stone developed in kidney (Sikkim: [79]).
306	*Mimosa pudica* L.	Herb	Fabaceae	Lazzawathi (Np); Jhapani (S)	200-1200	**Plant:** Juice given to relieve from diarrhoea (Ilam: [73]; Darjeeling: [86]), dysentery, and in treatment of hydrocele (Ilam: [73]). **Roots and leaves:** Paste used in case of piles (Darjeeling: [91]; Sikkim: [11], Database) and kidney problems (Sikkim: Database). **Roots**: Used in treating asthma, fever, cough, dysentery, vaginal and uterine complaint (Sikkim: Database). Paste applied externally to treat boils (Sikkim: [11]). Juice taken to cure epilepsy and sexual weakness also used to cure eye troubles (Jhapa: [66]). Powder used to clean tooth (Darjeeling: [74]).
307	*Moghania strobilifera* (L.) J. St.-Hil. ex Kuntze	Shrub	Fabaceae	Barakaulijhar (Np)	300-2300	**Roots:** Juice taken in indigestion, insomnia and epilepsy (Darjeeling: [74]).
308	*Mucuna macrocarpa* Wall.	Shrub	Fabaceae			**Seeds:** Powder taken as anthelmentic (Sikkim: [11]).
309	*Mucuna monosperma* Wall.	Shrub	Fabaceae	Baldengra (Np, Li)		**Seeds:** Act as expectorant in cough (Sikkim: [79]).
310	*Mucuna pruriens* (L.) DC.	Herb	Fabaceae	Kauso (Np); Etka (S)	100-1700	**Leaves:** Paste used in boils, blisters, and ulcers (Jhapa: [66]). **Roots and seeds:** Used as antipyretic and aphrodisiac (Panchthar: [67]). **Roots:** Used in delirium (Jhapa: [66]). **Seeds:** Used as medicine (KL Bhutan: [71]).
311	*Oxytropis japonica* Maxim.	Herb	Fabaceae			**Whole plant:** Used as medicinal (KL Bhutan: [71]).
312	*Tamarindus indica* L.	Tree	Fabaceae	Jojo (S) Imli, Titri (Np), Titri (Me)	200-900	**Bark:** Decoction given in paralysis, ulcers, and inflammations. Infusion along with bark of *Ziziphus mauritiana, Anthocephalus chinensis, Shorea robusta, Streblus asper* and black salt given to cure flatulence in cattle (Jhapa: [66]). **Fruit:** Edible (Jhapa: [92]). Used for cough and blood disorders (Sikkim: Database). **Seeds:** Paste eaten to cure stomachache (Jhapa: [68]).
313	*Vigna vexillata* (L.) A. Rich.	Herb	Fabaceae	Bir ghangra (S)		**Tubers and seeds:** Eaten as vegetable (Jhapa: [68]).
314	*Castanopsis hystrix* Miq.	Tree	Fagaceae	Patle Katus (Np)	1800-2400	**Fruit:** Eaten raw (Darjeeling: [80]; Ilam: [94]; Sikkim: [72, 89]).
315	*Castanopsis indica* (Roxb.) Miq.	Tree	Fagaceae	Katoos (Np)	1200-2900	**Fruit:** Roasted and consumed (Ilam: [94]).
316	*Castanopsis tribuloides* (Sm.) A. DC.	Tree	Fagaceae	Musure Katus (Np)	450-2300	**Fruit:** Edible (Sikkim: [89]). **Leaves:** Good ingredients for compost (Sikkim: [89]).
317	*Quercus glauca* Thunb.	Tree	Fagaceae	Phalat (Np); Yahi (Li)		**Bark:** Paste applied on bone fracture (KL Nepal: [53]).
318	*Quercus griffithii* Hook.f. & Thomson ex Miq.	Tree	Fagaceae	Sisi (Dz); Benangshing (Sh)		**Seeds:** Used as medicine (KL Bhutan: [71]).
319	*Gynocardia odorata* Roxburgh	Tree	Flacourtiaceae	Gantay (Np)	800-1000	**Fruit:** Used to extract oil (KL Bhutan: [93]). Juice taken or eaten raw in fever (Darjeeling: [78]). **Seeds:** Seed oil applied on skin diseases and leprosy (Sikkim: [75]), also used for massage purpose for infants (Sikkim: [79]). Ripen seeds roasted and the oil extracted to use in various purposes (Ilam: [94]).
320	*Gentiana grandiflora* Laxm.	Herb	Gentianaceae			**Whole plant:** Used as medicine (KL Bhutan: [71]).
321	*Gentiana stylophora* C.B.Clarke	Herb	Gentianaceae			**Floral parts:** Used as medicine (KL Bhutan: [71]).
322	*Gentiana urnula* Harry Sm.	Herb	Gentianaceae			**Whole plant:** Used as medicine (KL Bhutan: [71]).
323	*Gentiana veitchiorum* Hemsl.	Herb	Gentianaceae	Gangi- Pangenmotok (Dz)		**Whole plant/flowers:** Used as medicine (KL Bhutan: [71]).
324	*Halenia elliptica* D.Don	Herb	Gentianaceae			**Whole plant:** Used as medicine (KL Bhutan: [71]).
325	*Swertia angustifolia* Buch. Ham. ex D. Don	Herb	Gentianaceae	Vaale Chirayito (Np)	600-3300	**Plant:** Extract used to treat fever, cough, and cold (Ilam: [73]).
326	*Swertia bimaculata* (Sieb. & Zucc) C. B. Clarke	Herb	Gentianaceae	Chiraito, Tite (Np)	900-3700	**Plant:** Used to treat diarrhea, dysentery, and fever (Taplejung: [81]).
327	*Swertia chirayita (Roxb. ex Fleming) Karsten	Herb	Gentianaceae	Tikta (Km, Sh, Wi); Sungkhinwa (Li); Chiraito, Chiraita, Tite (Np); Khalu (Nw); Kuple (Ri); Kirattikta (Sn); Timda (Tm); Gya-Tig, Tigta (Tb)	1200-3000	**Plant and seeds:** Plant infusion and crushed seeds considered most effective in treating fever (Taplejung: [81]; KL Nepal: [83, 103]; Ilam: [73, 76]), asthma, cold, and cough (Darjeeling: [80]; Taplejung: [81, 87]; KL Nepal: [83, 103]; Sikkim: [11]; Ilam: [73, 76]). **Whole plant:** Used as medicine (KL Bhutan: [71]). Used to treat ulcer, asthma, inflammation, and piles (Darjeeling: [80]). Taken as tonic and also in leucoderma and skin diseases (Darjeeling: [69, 78, 80]). Decoction taken to cure cold, cough, diarrhea, and stomachache (Darjeeling: [69]; Sikkim: [11, 79]). **Shoots**: Taken in dyspepsia (Sikkim: [75]). **Leaves:** Mixed with young stem of *Achyranthes aspera* and stem bark of *Phyllanthus emblica* and decoction given to cure fever and cholera (Jhapa: [66]).
328	*Swertia multicaulis* D. Don	Herb	Gentianaceae	Sharma Guru (Np, Tm); Sepu Gundum (Sh, WI)	4000-4900	**Roots:** Decoction used to treat fever, cough, bodyache, and internal injuries; paste applied to prevent bleeding and infection from cuts and wounds (Taplejung: [81, 82, 87, 88]).
329	*Swertia nervosa* (G. Don) C. B. Clarke	Herb	Gentianaceae	Chiraito, Bhale Chiraito, God Tito (Np)	700-3000	**Roots:** Used to treat fever, cough, and cold (Taplejung: [82, 88]).
330	*Geranium donianum* Sweet	Herb	Geraniaceae	Ragatgeri (Np)	3200-4800	**Plant:**Juice consumed in renal complications and dysentery (Darjeeling: [74]).
331	*Geranium lambertii* Sweet	Herb	Geraniaceae			**Roots:**Used as medicine (KL Bhutan: [71]).
332	*Geranium nepalense* Sweet	Herb	Geraniaceae	Bhanda (Np)		**Whole plant:** Used as astringent (Sikkim: [75]).
333	*Aeschynanthus parviflorus* (D.Don) Spreng.	Shrub	Gesneriaceae			**Roots:** Decoction used to treat fever (Darjeeling: [78]).
334	*Didymocarpus villosus* D.Don	Herb	Gesneriaceae	Kumkum (Np)	900-2400	**Leaves:** Smoked for its laxative action (KL Nepal: [53]).
335	*Dichroa febrifuga* Lour.	Shrub	Hydrangeaceae	Aseru, Basauli, Bhahak, Pahare Basak, Vasak (Np); Khasrte, Patre, Polokamji (Gr); Kiplisang (Ri), Dharmen (Tm); Borang-yangshabu (Sh)	900-2500	**Roots:** Used as medicine (KL Bhutan: [71]). Used as tonic (Sikkim: [75]). Decoction taken to treat malarial fever (Ilam: [76]; Taplejung: [87]; Sikkim: [91], Database). **Roots and leaves:** Decoction taken for cough and fever (Darjeeling: [78, 80]; Ilam: [76]; Sikkim: [11, 89]). **Fruit:** Used as febrifuge (Panchthar: [67]). Ink prepared from berries (Sikkim: [11]).
336	*Curculigo orchioides* Gaertn.	Herb	Hypoxiadaceae	Kalo musali, Musali Kanda (Np); Gahot (S)	500-1100	**Roots/rhizomes:** Taken in stomachache, physical weakness (Jhapa: [66]), jaundice, cholera, and diarrhea (Darjeeling: [49]; Panchthar: [67]). Rhizome paste used against skin complaints, stomach ulcer, white discharge in women and dyspepsia (Darjeeling: [49]). Infusion used in gastritis and piles (Darjeeling: [49]).
337	*Belamcanda chinensis* (L.) Redoute	Herb	Iridaceae	Tarware phool (Np)	100-2200	**Rhizomes:** Freshly collected and eaten in stomachache and also taken orally as antidote to food poisoining (Darjeeling: [52]).
338	*Iris decora* Wall.	Herb	Iridaceae			**Fruits:** Used as medicine (KL Bhutan: [71]).
339	*Engelhardia spicata* Lesch. ex Blume	Tree	Juglandaceae	Yakpohama (Li), Mauwa (Np), Bokto, Oksipou (Ri)	400-2500	**Bark:** Used in diarrhea and dysentery; paste used in bone fracture (Taplejung: [88]).
340	*Juglans regia L.	Tree	Juglandaceae	Okhar (Np)	1000-2000	**Bark:** Used for dye and acts as detergent (Darjeeling: [80]; Sikkim: [89]). Juice taken to get rid from intestinal worms (Darjeeling: [86]; Sikkim: [11, 79]). **Stem bark:** Decoction taken to cure arthritis, rheumatism, skin diseases and toothache (Sikkim: [79]). **Bark and leaves:** Juice used as fish poison (Sikkim: [11]). **Leaves:** Paste applied on the hoof diseases of cattle (Sikkim: [97]) **Fruit:** Oil used for headache (Darjeeling: [80]). Used in rheumatism (Darjeeling: [91]). **Nuts:** Edible (Darjeeling: [80]; Ilam: [94]; Sikkim: [89]). Shell crushed and obtained black color (Sikkim: [11]).
341	*Juglans regia* var *kamaonia* L.	Tree	Juglandaceae	Himalayan Walnut (Eg); Akhor (Gr); Katutung, Takshing, Koto (Gr-Mn); Khayusin, Khesik (Li); Okhar, Hande Okhar (Np); Khaisi (Ri); Akshotak (Sn)	1200-3000	**Bark:** Paste used for poisoning fish. **Fruit:** Edible (KL Bhutan: [70]). Boiled in water to extract dye. Used to treat pneumonia and wounds (Panchthar: [67]). **Seeds:** Eaten as tonic or to treat throat pain; hard cover of the fruit applied on gout or to treat throat and chest pain (Taplejung: [87, 88]).
342	*Juncus grisebachii* Buchenau	Herb	Juncaceae	Juncus (C)		**Whole plant:** Used as medicine (KL Bhutan: [71]).
343	*Anisomeles indica* (L.) Kuntze	Herb	Lamiaceae	Nirepati, Jhusule, Rato Charpate, Rato Pat (Np); Jinting (Sh)	200-2400	**Roots:** Juice taken to cure fever, soar throat, diarrhea, and dysentery (Taplejung: [88]).
344	*Clerodendrum infortunatum* L.	Shrub	Lamiaceae	Chitu (Np)		**Leaves:** Used as anthelminthic, fresh juice used as tonic and febrifuge (Sikkim: Database).
345	*Clerodendrum viscosum* Vent.	Herb	Lamiaceae	Rajbeli (Np); Lakhanaat (Me); Varni (S)	100-1500	**Plant:** Infusion boiled in water along with the leaves of *Azadirachta indica* and bath to treat scabies and skin diseases (Jhapa: [66]). **Roots:** Paste used as anti-poison (Jhapa: [68]). **Leaves:** Paste used as hair tonic (Jhapa: [66]). Leaf juice consumed to treat leucoderma and hydrophobia (Darjeeling: [74]).
346	*Clinopodium umbrosum* (M.Bieb.) C. Koch	Climber	Lamiaceae	Suparnasa (Np)	180-3400	**Leaves:** Juice used to treat cuts and burns (Panchthar: [67]).Eaten as vegetable to maintain good health (Darjeeling: [74]).
347	*Colebrookea oppositifolia* Sm.	Herb	Lamiaceae	Dhursuli (Np); Dhusor (Me)	250-1700	**Leaves:** Paste applied in wounds and inflammation of skin (Jhapa: [68]). Juice used to treat cuts and bruises (Panchthar: [67]).Juice taken in dysentery (Sikkim: [79]). Bud extract applied in opthalmic problems (KL Nepal: [53]).
348	*Elsholtzia blanda* (Benth.) Benth.	Herb	Lamiaceae	Jungali Tulsi, Ban Silam (Np)		**Roots:** Powder paste with mustard oil applied on the scabies affected area of cattle (Sikkim: [84]). **Leaves**: Juice given in diarrhoea (Darjeeling: [74]). **Shoots:** Taken in gastritis (Sikkim: [75]).
349	*Elsholtzia fruticosa* (D.Don) Rehder	Shrub	Lamiaceae	Chhinik (Np); Aamgora, Kansata (Sh); Jirug Serpo (Km, Tb); Furmi (Wl)	1800-4200	**Plant:** Religious value (Taplejung: [81]). **Leaves and spikes:** Used as incense (Taplejung: [81]). **Roots**: Given against tonsilitis (Darjeeling: [52]).
350	*Eriophyton wallichii* Benth.	Herb	Lamiaceae			**Whole plant:** Used as medicine (KL Bhutan: [71]).
351	*Hyptis suaveolens* (L.) Poit.	Shrub	Lamiaceae	Arridari (S)	150-1000	**Plant:** Paste applied on skin infections (Jhapa: [66]). Leaves: Juice warmed and applied over lice and parasities infected area (Darjeeling: [74]).
352	*Isodon coetsa* (Buch.-Ham. ex D.Don) Kudô	Herb	Lamiaceae	Mire (Np)	600-3400	**Leaves and shoots:** Used to treat cuts and wounds (Panchthar: [67]).
353	*Leucas cephalotes* (Roth) Spreng.	Herb	Lamiaceae	Drona puspi (Np); Gante Jhar (Np)	150-2400	**Plant:** Used to treat pneumonia and wounds (Panchthar: [67]) **Leaves:** Decoction given in menstrual disorders (KL Nepal: [53]).
354	*Leucas indica* (L.) R. Br. ex Vatke	Herb	Lamiaceae	Khaangkareh (Me); Gummi (Np); Durup (S)	70-1000	**Leaves:** Crushed with *Euphorbia hirta* and let to inhale for sinusites and nasal infection (Jhapa: [68]). Decoction massaged on forehead to relieve headache; drops poured in nostril to cure sinusitis and earache; juice taken in asthma, and also applied in geneital organs to cure venereal diseases (Jhapa: [66]).
355	*Leucosceptrum canum* Sm.	Herb	Lamiaceae	Cheeongkung (L)	1000-2600	**Roots and leaves:** Used for epilepsy and wound (Sikkim: [75]).
356	*Melissa axillaris* (Benth.) Bakh. f., Herb		Lamiaceae	Sugandhi (Np)		**Leaves:** Juice taken with honey to cure fever (Darjeeling: [78]).
357	*Mentha arvensis* L.	Herb	Lamiaceae	Padina (Np, Li)	1200-2000	**Leaves:** Juice given to treat rheumatism, fever, weakness, ulcer, wounds, jaundice, cough, asthma and cuts (Ilam: [76]). Fresh leaves chewed during gastritis and acidity (Sikkim: [79]).
358	*Mentha piperita* L.	Herb	Lamiaceae	Pudhina (Np)		**Plant:** Paste taken in bodyache (Darjeeling: [52]). **Leaves:** Juice, paste or oil taken in painful urination, stomach problems and indigestion (Ilam: [76]).
359	*Mentha* sp.	Herb	Lamiaceae			**Leaves:** Used as spices (KL Bhutan: [70]).
360	*Ocimum tenuiflorum* L.	Herb	Lamiaceae	Tulasipatta (S)	400-900	**Leaves:** Leaves are pounded and given with unboiled rice in cough and bronchitis (Jhapa: [66]). Chewed to cure mouth ulcers (Sikkim: [79]). Juice given in cardiopathy, asthma, bronchitis, snake bite, urinary disorders (Ilam: [76]).
361	*Orthosiphon incurvus* Benth.	Herb	Lamiaceae	Tite (Np)		**Plant:** Juice given to cure tooth decay, diarrhoea, wounds, and cuts (Ilam: [73, 76]).
362	*Perilla frutescens* (L.) Britton	Herb	Lamiaceae	Silam (Np, Li)	600-2400	**Seeds:** Dried seeds chewed to cure cough and nausea (Sikkim: [79]).
363	*Phlomoides rotata* (Benth. ex Hook.f.) Mathiesen	Herb	Lamiaceae			**Whole plant:** Used as medicine (KL Bhutan: [71]).
364	*Pogostemon amarantoides* Benth.	Herb	Lamiaceae	Solomon, Namnam (Bhu)	900-2100	**Young leaves**: Used as vegetables (Bhutan: [102]).
365	*Pogostemon benghalensis*(Burm. f.) Kuntze	Shrub	Lamiaceae	Rudilo (Np)	150-1300	**Young shoots:** Grounded and given to treat sores of mouth and tounge (Jhapa: [66]).
366	*Salvia campanulata* Wall. ex Benth.	Herb	Lamiaceae			**Flowers:** Used as medicine (KL Bhutan: [71]).
367	*Salvia moorcroftiana* Wall. ex Benth.	Herb	Lamiaceae		2000-3000	**Whole plant:** Given against diabetes (Darjeeling: [52]).
368	*Salvia* sp.	Herb	Lamiaceae			**Flower:** Used as medicine (KL Bhutan: [71]).
369	*Siphocranion macranthum* (J. D. Hooker) C. Y. Wu	Herb	Lamiaceae		1300-3200	**Shoots:** Taken in gastric (Sikkim: [75]).
370	*Holboellia latifolia* Wall.	Tree	Lardizabalaceae	Golfa (Np)	2400-3200	**Roots:** Effective for rheumatism (Sikkim: [75]). **Fruit:** Edible (Darjeeling: [72, 80]; Sikkim: [89]). **Stem:** Used to make bangles, which are believed to give from orthopedic problems (Darjeeling [80]).
371	*Cinnamomum bejolghota* (Buch.-Ham.) Sweet	Tree	Lauraceae	Bhalay Linkauli (Np)	600-1800	**Bark:** Used as condiment (Sikkim: [72]).
372	*Cinnamomum glaucescens (Nees) Hand.-Mazz.	Tree	Lauraceae	Phagpanengshing (Sh)		**Wood:** Used as incense (KL Bhutan: [70]).
373	*Cinnamomum impressinervium* Meisn.	Tree	Lauraceae	Sisi (Np)	1220-1830	**Seeds:** Edible (Darjeeling: [80]). **Leaves:** Used as a substitute of bay leaf (Sikkim: [72]).
374	*Cinnamomum tamala (Buch.-Ham.) Nees & Eberm.	Tree	Lauraceae	Sinkauli, Tejpat (Np); Pinge (Gur); Pataarangkhi (Rai); Shishi (She)	450-2000	**Bark:** Used as medicine (KL Bhutan: [71]). Given in gonorrhea (Sikkim: Database).Extract used in stomach disorders (Sikkim: [79]). **Leaves:** Used as stimulant in rheumatism, and also in colic and diarrhea (Sikkim: Database). Rubbed on the body to cure scabies (Sikkim: [11]), throat allergy and to increase appetite (Jhapa: [68]). Used as condiments (KL Bhutan: [70]). Ilam: [94]).
375	*Cinnamomum verum* J.Presl	Tree	Lauraceae			**Leaves:** Used as incense (KL Bhutan: [70]).
376	*Lindera neesiana* (Wall ex Nees) Kurz	Tree	Lauraceae	Gutum Phopri (Gr-Mn); Waregpa (Li); Sil Timmur, Phenlo Khapate (Np); Kongkochi (Ri); Kutumba (Tm)	1800-2700	**Bark and fruit:** Used as aromatic and carminative (Sikkim: [85]). **Fruit:** Used in cough, cold, fever, and cholera; fried in butter and used during gastritis; also used in pickle (Taplejung: [87, 88]). Given to treat headache (Panchthar: [67]). **Seeds:** Powder taken to stop vomiting (Sikkim: [11]).
377	*Litsea cubeba* (Lour.) Pers.	Tree	Lauraceae	Siltimur (Np); Tanghaercherkerng (L)		**Flowers:** Used in stomach disorders (Sikkim: [11, 75], Database). **Fruit:** Taken orally as carminative (Sikkim: [85]). Used to make *chutney* (Darjeeling: [80]; Sikkim: [11]). Dried fruits used in nausea and giddiness (Darjeeling: [80]).
378	*Litsea glutinosa* Lour.	Tree	Lauraceae	Kawala, Suppatnyok (Np)		**Leaves and bark:** Decoction taken to treat dysentery (Sikkim: [79]).
379	*Litsea monopetala* (Roxb.) Pers.	Tree	Lauraceae	Ratmanti, Sunyokkung (Np)		**Bark:** Decoction taken to treat diarrhea (Sikkim: [85]).
380	*Machilus edulis* King.	Tree	Lauraceae	Pumpsi, Lapche Kaulo, Lapchephal (Np)		**Fruit:** Edible (KL Bhutan: [70]; Ilam: [94]; Sikkim: [47, 89]). **Leaves**: Used as fodder (Sikkim: [89]).
381	*Machilus* sp.	Tree	Lauraceae			**Bark:** Used as incense (KL Bhutan: [70]).
382	*Persea odoratissima* (Nees) Kostermans	Tree	Lauraceae	Lalikaulo (Np)	1000-2000	**Fruit:** Edible (Sikkim: [89]). **Leaves:** Good fodder (Sikkim: [89]).
383	*Careya arborea* Roxb.	Tree	Lecythidaceae	Khumbhi (S)	200-600	**Bark:** Grounded with cumin seeds and given to treat indigestion and flatulence (Jhapa: [66]).
384	*Leea macrophylla* Roxb. ex Hornem.	Shrub	Leeaceae	Galeni, Galoni (Np)	1500-1700	**Root:** Used to cure snake bite (Panchthar: [67]). **Seeds:** Chewed to treat viral fever. Wrapped by cloths and tied around the neck of the children to cure stomach pain (Sikkim: [11]).
385	*Aloe vera* (L.) Burm. f.	Herb	Liliaceae	Ghiu kumari (Np)	1200-1400	**Plant:** Used for antihypergycemic effect (Sikkim: [96]). Purgative (Sikkim: Database) and used on burns and skin complaints (Jhapa: [66]; Panchthar: [67]; KL Nepal: [53]; Sikkim: Database, [79]). **Leaves:** Chewed to cure skin and uterine disorder and jaundice (Jhapa: [68]; Ilam: [73, 76]). Used as stomachic, tonic, purgative and anthelmintic. Juice put on head in high fever to reduce body temperature (Sikkim: [69]).
386	*Asparagus racemosus Willd.	Herb	Liliaceae	Kurilo (Np); Kedar nari (S); Ngalalkom (Sh); Ngakhacho (Dz)	300-2200	**Roots:** Used as medicine (KL Bhutan: [71]). Useful for diabetes, jaundice and urinary disorder (Sikkim: Database). Extract used in diabetes and tuberculosis (Darjeeling: [52]) and applied externally on bone fracture and joint dislocation (Darjeeling: [52]). Also used as diuretic, demulcent, aphrodisiac, refrigerant, tonic, expectorant, astringent, and appetizer (KL Nepal: [83]; Darjeeling: [52]; Panchthar: [67]). Powder taken to enhance lactation for mothers and also useful in anemia and diarrhoea (Ilam: [73, 76, 77]). Grounded with root of *Musa paradisiaca* and drunk to cure gonorrhea; decoction given in fever (Jhapa: [66]). Mixed with hay or grain to feed the cattle for fortnight to increase milk-yield (Sikkim: [84]). Paste administered orally in fever, cough and cold. **Fruit:** Eaten to treat pimples (Sikkim: [79]). **Shoots:** Cooked as vegetable (Taplejung: [82]; KL Nepal: [83]; Panchthar: [67]).
387	*Disporum cantoniense* (Lour.) Merr.	Herb	Liliaceae	Mhajari (Np)	700-3000	**Roots:** Used to treat sprains (Panchthar: [67]).
388	*Fritillaria cirrhosa* D. Don	Herb	Liliaceae	Snake's head fritillary (Eg); Ban lasun, Kakolee (Np)	3000-4600	**Bulb:** Boiled and eaten (KL Nepal: [83]). Used in tuberculosis (Sikkim: [75]) and to treat asthma (KL Nepal: [83]; Sikkim: [75]), bronchitis, and bleeding during cough (Sikkim: [75]).
389	*Fritillaria delavayi* Franch.	Herb	Liliaceae	Tsika (Dz)		**Bulb:** Used as medicine (KL Bhutan: [71]).
390	*Gloriosa superba* L.	Herb	Liliaceae	Langarey Tarul (Np)	400-2200	**Roots and flowers:** Useful in chronic ulcers, leprosy, piles, and abdominal pains (Sikkim: Database).
391	*Lilium nepalense* D. Don	Herb	Liliaceae	Ban Lasun (Np)	2300-3400	**Plant:** Powder used to flavouring dishes; juice taken as tonic (Ilam: [73]). **Bulb**: Used to treat scabies and boils (Panchthar: [67]).
392	*Paris polyphylla* Smith	Herb	Liliaceae	Love Apple (Eg); Satuwa (Gr); Tangma (Km); Satuwa, Tintale Banko (Np); Haimavati (Sn); Natar Dhap (Tm)	1800-3500	**Roots:** Used as antidotes (Sikkim: [75]) and in stomachache (Taplejung: [81, 82]) and fever (Taplejung: [81, 82]; Sikkim: [75]). Powder applied to heal wound (Darjeeling: [49]; Taplejung: [81]; KL Nepal: [83]). Infusion taken as tonic and also used in diarrhea, dysentery (KL Nepal: [83]; Ilam: [73]) and fever (Darjeeling: [78]). Infusion taken to treat respiratory disorders and worms (Ilam: [76]).
393	*Polygonatum hookeri* Baker	Herb	Liliaceae	Ranye (Km), Pangi Ranye (Tb)	2900-5000	**Roots:** Eaten raw (Taplejung: [87]).
394	*Smilax bracteata* C. Presl subsp. verruculosa (Merrill) T. Koyama	Climber	Liliaceae	Kukurdine (Np)	1900	**Stem:** Used for brushing teeth to cure pyorrhoea and gingivitis (Darjeeling: [52]).
395	*Smilax ovalifolia* Roxb. ex. D. Don	Climber	Liliaceae	Kukurdaino (Np)	200-1000	**Roots and fruit:** Juice used to cure veneral diseases, rheumatism, and wounds (Ilam: [73, 76]). **Leaves and shoots:** Used as curry (Ilam: [94]).
396	*Buddleja asiatica* Lour.	Shrub	Loganiaceae	Bhinsenpatee (Np); Pndam (L)	350-2000	**Stem, leaves and flowers:** Used in skin complaints and as abortificant (Sikkim: [75], Database).
397	*Dendrophthoe falcata* (L.f.) Etting.	Herb	Loranthaceae	Mandargon banda (S); Aijeru (Np)	150-900	**Leaves:** Paste applied externally to treat skin diseases (Jhapa: [66]).
398	*Scurrula elata* (Edgew.) Danser	Shrub	Loranthaceae		1600-2700	**Leaves:** Used for making tea leaf for local suja (Bhutan: [99]). **Bark**: Paste applied on bone fracture (KL Nepal: [53]).
399	*Viscum articulatum* Burm.f.	Shrub	Loranthaceae	Hadchur (Np); Hadjor, Kathkomjunga (S)	200-1700	**Plant:** Used to treat bone fracture (Jhapa: [66]; Panchthar: [67]); also given in ulcers, epilepsy, muscular pains (Sikkim: Database), injuries, and fracture (Darjeeling: [80]; Sikkim: [11, 79], Database). **Stem:** Used in body pain, fracture, and fever (Ilam: [76]; Sikkim: [75]). Paste used in sprain and rheumatism (Jhapa: [66]).
400	*Woodfordia fruticosa* (L.) Kurz	Shrub	Lythraceae	Dhairo (Np); Icha (S)	200-1800	**Bark**: Used for gastric trouble. **Bark and flowers:** Used in burns, dysentery, and fracture (KL Nepal: [53]; Panchthar: [67]). **Flower:** Dried and taken in piles, liver complaints. Honey like secretion consumed (Ilam: [94]). **Leaves:** Given to cattle to cure ulcer (Sikkim: [97]). Decoction taken in malarian fever (Jhapa: [66]).
401	*Michelia champaca* L.	Tree	Magnoliaceae	Chanpak (Eg); Oulichamp (Np)		**Flowers:** Taken for stomachache (Ilam: [76]; Sikkim: [85]) and as carminative in the treatment of dyspepsia (Sikkim: [85]). **Flowers and fruit:** Use to extract dye (KL Bhutan: [70]).
402	*Hibiscus rosa-sinensis* L.	Shrub	Malvaceae	Jawa Kusum (Np)	900-1400	**Flowers:** Young flowers given against tonsillitis. **Leaves and flowers:** Juice used to cure dandruff and hair problems (Darjeeling: [52]).
403	*Hibiscus sabdariffa* L.	Herb	Malvaceae	Bel Chandan (Np); Maharetha (Me)	1000-1500	**Leaves:** Paste with leaves of *Lawsonia inermis* applied on the foot to cure wound caused by muddy water during rainy season (Jhapa: [68]).
404	*Malva verticillata* L.	Shrub	Malvaceae	Halemetok (Dz)		**Flowers:** Used as medicine (KL Bhutan: [71]).
405	*Sida acuta* Burm. f.	Shrub	Malvaceae	Khareto (Np)	250-3700	**Stem:** Paste applied on bone fracture (Darjeeling: [74]).
406	*Sida cordifolia* L.	Shrub	Malvaceae	Bamonmara (Np)	500-1100	**Roots:** Half cup root juice and half-tablespoon sugar candy mixed together is given once daily till cured (Darjeeling: [105]).
407	*Sterculia villosa* Roxb.	Tree	Malvaceae	Odal (Np)		**Bark:** Used to make ropes (KL Bhutan: [100]).
408	*Urena lobata* L.	Herb	Malvaceae	Samthai (Me)	200-1300	**Leaves:** Paste with aerial part of *Drymaria cordata* applied to cure skin infection and eczema (Jhapa: [68]).
409	*Melastoma malabathricum* L.	Shrub	Melastomataceae	Augeri, Chulesi (S & Np)	200	**Leaves:** Used in fever; decoction given to cattle (Sikkim: [97]). **Fruit:** Edible (Jhapa: [92]). **Stem and root**s: Bark paste applied on wounds and skin disease (Darjeeling: [74]).
410	*Osbeckia nepalensis* Hook.	Herb	Melastomataceae	Angeri, Lattey (Np)	450-2300	**Roots:** Decoction given in urinary complaints and diabetes. **Leaves:** Extract of young leaves or tender shoots applied on forehead and to treat pneumonia, fever and common cold (Darjeeling: [74]). **Leaves and flowers:** Paste used in foot sores of cattle (Darjeeling: [49]).
411	*Azadirachta indica A. Juss.	Tree	Meliaceae	Meemee (S); Neem (Np); Nimbilai (Me)	60-1200	**Plant:** Used as an antiseptic and febrifuge. Useful in treatment of small pox, and as tooth brush, prophylactic for mouth and teeth (Sikkim: Database). **Leaves:** Cooked with rice and eaten during the name giving ceremony of newly born child. Boiled in water and bath taken with warm water to cure scabies and eczema. Dried leaf power taken as blood purifier (Jhapa: [92]). Juice taken for blood purification and intestinal worms, also applied externally on wounds, sores, blisters, ans skin diseases (Jhapa: [66]). Fresh/dried leaves chewed to control diabetes (Sikkim: [79, 96]). Bark powder used as insecticide (Sikkim: [79]).
412	*Dysoxylum hamiltonii* Blume.	Tree	Meliaceae	Sipochikang (Np)		**Bark:** Decoction taken orally to treat stomachache (Sikkim: [85]).
413	*Melia azedarach* L.	Tree	Meliaceae	Bakaina (Np); Bokom Bana (S)	700-1100	**Roots:** Used as astringent, and in biliousness, heart pain, vomiting, and leucoderma. **Leaves:** Juice taken orally as anthelmintic (Sikkim: [85]). **Bark**: Used as anthelmintic (Jhapa: [66]). **Fruit**: Used in leprosy (Jhapa: [66]).
414	*Toona ciliata* M. Roem.	Tree	Meliaceae	Tooni (Np); Toon (S)	200-1700	**Bark:** Pounded and given to cure toothache (Jhapa: [66]; Sikkim: [79]). **Flowers and fruit:** Use to extract dye (KL Bhutan: [70]). **Fruit:** Used for chestpain, fever and measles (Sikkim: [79]).
415	*Trichilia connaroides* (Wight & Arn.) Bentv.	Tree	Meliaceae	Aankha Taruwa (Np)	700-2400	**Leaves:** Decoction taken to treat cholera (Sikkim: [85]). **Leaves and fruit:** Useful to treat cholera (Panchthar: [67]).
416	*Cissampelos pareira* L.	Climber	Menispermaceae	Batulpate (Np); Tejomala (S)	500-1000	**Plant:** Extract given to treat diarrhea (Jhapa: [66]; Sikkim: [11]), dysentery, urinary disorders, and indigestion (Jhapa: [66]). **Roots:** Used as antidote; paste taken orally in stomach pain, malarian fever, and cold (Jhapa: [66]). Decoction consumed in empty stomach to cure diabetes (Darjeeling: [74]). Extract given to treat blood in urine (Sikkim: [84]). **Leaves:** Paste applied on wound and juice taken to cure stomach pain (Sikkim: [11]). **Roots and leaves**: Useful in cough, gastric troubles, and sore throat (Panchthar: [67]).
417	*Stephania glabra* (Roxb.) Miers	Climber	Menispermaceae	Tamarke (Np)	1000-2500	**Tubers:** Powder used in diabetes, tuberculosis, asthma, and fever (Darjeeling: [49, 78]). Oil used externally in the treatment of leucoderma, leprosy and other skin complaints (Darjeeling: [49]). **Stem:** Bark used to extract fibres (Darjeeling: [49]). **Plant:** Useful in jaundice (Darjeeling: [105]).
418	*Stephania glandulifera* Miers	Climber	Menispermaceae	Tamarkay (Np)		**Tubers:** Eaten in jaundice (Sikkim: [75]).
419	*Stephania japonica*(Thunb.) Miers	Climber	Menispermaceae			**Root:** Dipped in water and sprinkled in poultry farm to prevent from birld flu. **Leaves:** Paste applied on boils for opening (Sikkim: [11]).
420	*Tinospora sinensis (Lour.) Merr.	Climber	Menispermaceae	Garjo, Gurjo (Np); Sarasatilata (S)		**Roots:** Useful in diabetic. Extract used in menstruation disorders and piles (KL Nepal: [53]). **Stem and roots:** Infusion given in fever (Darjeeling: [69, 78]). Used to make broom (KL Bhutan: [100]). **Leaves:** Warmed and wrapped around the fractured and painful joints (Jhapa: [66]). **Fruit:** Boiled in milk and drunk to get rid from tuberculosis (Darjeeling: [74]). Used as medicine (KL Bhutan: [71]).
421	*Artocarpus heterophyllus* Lam.	Tree	Moraceae	Rukh Kathar (Li)	1000	**Latex:** Applied on the boils and on the fractured bone (Sikkim: [79]).
422	*Artocarpus lakoocha* Wall. ex Roxb	Tree	Moraceae	Badahar, Barar (Li)	100-1000	**Latex:** Applied on boils and on fractured bone (Sikkim: [79]). **Fruit:** Edible (KL Bhutan: [70]). Raw fruits used as vegetable and ripen fruits eaten raw (Ilam: [94]).
423	*Ficus auriculata* Lour.	Tree	Moraceae	Nimaro, Nibaro (Np)	250-1700	**Roots and fruit:** Chewed in constipation and warts (Ilam: [73]). **Fruit:** Consumed (Ilam: [94]).
424	*Ficus benghalensis* L.	Herb	Moraceae	Banidare (S); Bar (Np)	500-1200	**Fruit:** Edible (Jhapa: [92]). Also given in fever (Jhapa: [66]).
425	*Ficus elastica* Roxb. ex Hornem.	Tree	Moraceae	Brongshig (Sh)		Exude used as gum (KL Bhutan: [70]).
426	*Ficus hirta* Vahl	Tree	Moraceae		900	**Root:** Decoction used to treat food poisoning (Sikkim: [11]).
427	*Ficus hispida* L.f.	Tree	Moraceae	Setapodo (S); Khasreto (Np)	450-1100	**Fruit:** Edible (Jhapa: [92]).
428	*Ficus racemosa* L.	Tree	Moraceae	Loa (S); Gular Dumri (Np)	300m	**Fruit:** Edible (Jhapa: [92]). Also taken to enhance lactation (Jhapa: [66]). **Latex**: Applied on boils and blisters (Jhapa: [66]).
429	*Ficus religiosa* L.	Tree	Moraceae	Peepal (Np)	150-1500	**Latex:** Applied on the boils on the tongue (Ilam: [73]).
430	*Ficus semicordata* Buch.-Ham. ex Sm.	Tree	Moraceae	Khasrey, Khaneu, Khaniu (Np)	200-2700	**Bark and latex:** Applied on boils to check infection (Sikkim: [11], Database). **Latex:** Applied on fresh cut (Panchthar: [67]). **Fruit:** Edible (Ilam: [94]; Jhapa: [92]).
431	*Ficus subincisa* Buch.-Ham. ex Sm.	Herb	Moraceae	Lute Khaneu (Np)	400-2400	**Latex:** Freshly collected and applied externally on boils (Darjeeling: [52]).
432	*Ficus virens* Dryand.	Tree	Moraceae	Kabra (Np)	500	**Leaf buds:** Young unopened leaf buds boiled and used as pickle (Ilam: [94]).
433	*Morus alba* L.	Tree	Moraceae			**Leaves, stem and roots:** Contains active phytochemical to lower blood glucose level (Sikkim: [96]). **Leaves:** Tender leaves chewed in curing inflammation of vocal cord and hoarse voice (Sikkim: [11]). **Fruit:** Edible (KL Bhutan: [70]; Sikkim: [95]). Used to make jam, jellies and drinks (Ilam: [94]).
434	*Morus australis* Poir.	Shrub	Moraceae	Kodaz (S); Kimu (Np)	900-2400	**Bark and leaves:** Decoction used to cure sore throat (Sikkim: [11]). **Fruits:** Edible (KL Bhutan: [70]; Jhapa: [92]; Sikkim: [11]). **Seeds:** Extract applied to treat foot cracks (Sikkim: [11]).
435	*Morus macroura* Miq.	Tree	Moraceae	Kimbu (Np); Tshendey (Dz); Sengdengshing (Sh)		**Stem:** Extract from heartwood used as medicine (KL Bhutan: [71]). **Fruit:** Edible (KL Bhutan: [70])
436	*Streblus asper* Lour.	Tree	Moraceae	Khaksi (Np); Sada (S)	100-500	**Fruit:** Edible (Jhapa: [68]). **Latex:** Used to stop bleeding from freshly cut wound (Jhapa: [66]).
437	*Moringa oleifera* Lam.	Tree	Moringaceae	Munga (S); Sajiwang, Sajana (Np); Sajanamakharia (Me)	150-1100	**Leaves, flowers and fruit:** Used as vegetable Ilam: [94]; Jhapa: [92]). Cooked vegetable taken to control blood pressure (Jhapa: [68]). **Bark:** Paste mixed with black goat's milk and taken to cure tuberculosis (Jhapa: [66]). **Stem:** Paste used by women for long hairs (Jhapa: [66]).
438	*Musa balbisiana* Colla	Herb	Musaceae	Bankera (Np)	200-600	**Fruit:** Green fruits used as vegetable after boiling and spath for pickle (Ilam: [94]).
439	*Musa paradisiaca* L.	Herb	Musaceae	Kera (Np)		**Root bark:** Pounded together with stem bark of Mango, Jack fruit, *Ziziphus mauritiana, Shorea robusta* and *Azadirachta indica* and taken to cure diarrhea and dysentery (Jhapa: [66]). **Sap:** Taken to cure fever (Sikkim: [11]).
440	*Musa* sp.	Herb	Musaceae	Bankera (Np)		**Leaves:** Used in house construction, roofing, and for making temporary sheds, also used as fodder. **Flowers and fruit:** Edible (KL Bhutan: [70, 100]).
441	*Myrica esculenta* Buch.-Ham. ex D. Don	Tree	Myricaceae	Box Myrtle (Eg); Kaphal (Np, Gr); Katphala (Sn); Lalisa (Lm); Chakchansi, Jheremsi (Ri)	1200-2300	**Bark:** Powder mixed with tobacco and snuffed to treat sinusitis (Panchthar: [67]); mixture also given to livestock to cure worm infestation (Taplejung: [87, 88]). Paste applied to fix fractured bone and cure internal injuries (Taplejung: [87, 88]). Paste applied on the chest to get relief from cough and bronchitis (Ilam: [76]; Sikkim: Database). **Fruit:** Ripen fruits consumed (Ilam: [73, 94]).
442	*Myrica nagi* Thunb.	Tree	Myricaceae	Tsutsusey (Sh)		**Fruit:**Used as spices (KL Bhutan: [70]).
443	*Horsfieldia kingii* (J. D. Hooker) Warburg	Tree	Myristicaceae	Ramgua (Np)	300-1200	**Fruit:** Used to make pickle (Ilam: [94]).
444	*Knema angustifolia* Roxb.	Tree	Myristicaceae	Ramguwa (Np)		**Fruit:** Powder taken orally to treat diarrhea (Sikkim: [85]).
445	*Ardisia macrocarpa* Wall.	Tree	Myrsinaceae		1500-2400	**Fruit:** Edible (Sikkim: [47]).
446	*Ardisia solanacea* Roxb.	Tree	Myrsinaceae	Gulaich (S)	200-1100	**Bark:** Macerated with flowers of *Hibiscus rosa-sinensis* and taken to purify the blood and also to arrest bleeding (Jhapa: [66]).
447	*Embelia ribes* Burm.f.	Shrub	Myrsinaceae	Buibidans (Np)		**Seeds:** Used as medicine (KL Bhutan: [71]). Powdered with milk and given to dog for anthelmintic efficacies (Sikkim: [84]). **Fruit**: Crushed and given to pigs to kill tapeworm; also used as appetizer for cattle (Sikkim: [84]).
448	*Maesa chisia* Buch.-Ham. ex D. Don	Shrub	Myrsinaceae	Bilaune (Np); Purmu Kung (L)		**Roots and leaves:** Used as insecticide and anthelmintic (Sikkim: [75]).
449	*Eugenia kurzii* Duthie	Tree	Myrtaceae	Ambakay (Np)	500	**Fruit:** Pulps consumed (Ilam: [94]).
450	*Eugenia* sp.	Tree	Myrtaceae	Mantsisey (Sh); Nasi or Nyasey (Dz)		**Fruit:** Edible (KL Bhutan: [70]).
451	*Psidium guajava* L.	Tree	Myrtaceae	Amba (Np); Aprisam (S)	450-1200	**Bark:** Pounded with bark of mango and given to cure piles (Jhapa: [66]). **Leaves:** Tender shoots chewed during cough and sore throat (Sikkim: [79]). **Fruit and bark:** Semi ripe fruits and bark eaten to cure diarrhoea (Darjeeling: [86]).
452	*Syzygium cumini* (L.) Skeels	Tree	Myrtaceae	Sokod (S); Jamun (Np); Jamun (Me), Bjee (Sz), Dangbelingsae (Sh), Ngasi (Dz)	300-1200	**Bark:** Juice drunk in dysentery (Jhapa: [66, 68]) and diarrhea (Jhapa: [66]). **Leaves:** Extraction massaged on forehead of children for cooling and given orally to increase appetite (Jhapa: [66]). **Fruits:** Edible (Jhapa: [92]).Used as medicine (KL Bhutan: [71]).
453	*Euryale ferox* Saliab.	Herb	Nymphaeaceae	Poraini (S); Maknana (Np)	3000-4000	**Seeds:** Edible (Jhapa: [92]).
454	*Fraxinus floribunda* Wall.	Tree	Oleaceae	Lankuri (Np); Payjew (L)		**Bark:** Boiled and applied on gout (Sikkim: Database). Applied on bone fracture (Sikkim: [75]).
455	*Nyctanthes arbor-tristis* L.	Shrub	Oleaceae	Parijat (Np); Badibaha, Jhanti (S)	200-1200	**Bark:** Paste used for dislocated bones. **Leaves:** Boiled and decanted water taken to control malaria fever. **Flowers:** Offered to god and goddesses (Sikkim: Database). **Leaves and flowers**: Pounded with warm water and given to woman to ease expulsion of placenta (Jhapa: [66]).
456	*Epilobium angustifolium* L.	Herb	Onagraceae	Rosebay (Eg); Bahankot (Km); Seja (Sh, Wl); Char Pan, Chu Tsi (Tb)	3300-4000	**Leaves:** Dried and used as tea; given as fodder (Taplejung: [81, 88]).
457	*Bulbophyllum affine* Lindl.	Herb	Orchidaceae			**Plant:** Ornamental value (Bhutan: [99]). **Flowers**: Edible (Bhutan: [99]).
458	*Calanthe plantaginea* Lindley	Herb	Orchidaceae	Wangpeimo (Bhu)	1800-2500	**Plant:** Cooked and eaten as vegetable (Bhutan: [102]).
459	*Coelogyne cristata* Lindl.	Herb	Orchidaceae	Chaandi gaava (Np)	1000-2000	**Bulbs:** Paste used in sores and boils (Ilam: [76]).
460	*Coelogyne occultata* Hook.f.	Herb	Orchidaceae	Churchurbu (Sh)		**Bulb/stem:** Used as medicine (KL Bhutan: [71]).
461	*Cymbidium longifolium* D. Don	Herb	Orchidaceae		2100-2500	**Plant:** Used as ornamental (Bhutan: [99]).
462	*Dactylorhiza hatagirea (D.Don) Soo	Herb	Orchidaceae	Wangpo Lagpa (Km, Sh, Wl); Nahasihukchok (Li); Panch Aunle (Np); Hathejara (Np-Dl); Munjataka (Sn); Lovha (Sh); Wang Lag, Wangpo Lagpa (Tb)	2800-4200	**Tubers:** Used as tonic and given in dysentery (Sikkim: Database) and chronic fever (Darjeeling: [78]; Sikkim: Database). Decoction used to treat fever, diabetes (Ilam: [73, 76]) and diarrhoea (Ilam: [73, 76]; Sikkim: Database). Paste applied on cuts and bruises; extract taken orally to cure body ache (Darjeeling: [52, 80]). Paste taken in gastric complaints, jaundice, body ache, bone fracture, and in the formation of bone marrow (Darjeeling: [49]). **Young leaves and shoots**: Eaten as vegetable (KL Nepal: [83]). **Roots:** Used as nervine tonic and aphrodisiac (Sikkim: [69]).
463	*Dendrobium aphyllum* (Roxb.) C.E.C.Fisch.	Herb	Orchidaceae			**Stem:** Used as medicine (KL Bhutan: [71]).
464	*Eulophia dabia* (D. Don) Hochr.	Herb	Orchidaceae	Hattipaila (Np)		**Tubers:** Juice taken orally as appetizer (Sikkim: [85]).
465	*Gymnadenia crassinervis* Finet	Herb	Orchidaceae	Wanglak (B)		**Roots:** Used as medicine (KL Bhutan: [71]).
466	*Ponerorchis chusua* (D. Don) Soo	Herb	Orchidaceae	Ongri (Sh); Wangla, Marpo, Wanglag Mempa (Tb)	2400-4900	**Tubers:** Rosted and eaten (Taplejung: [87]).
467	*Pedicularis anas* Maxim.	Herb	Orobanchaceae			**Whole plant:** Used as medicine (KL Bhutan: [71]).
468	*Pedicularis decorissima* Diels	Herb	Orobanchaceae			**Whole plant:** Used as medicine (KL Bhutan: [71]).
469	*Pedicularis longiflora* Rudolph	Herb	Orobanchaceae			**Whole plant:** Used as medicine (KL Bhutan: [71]).
470	*Pedicularis oederi* Vahl	Herb	Orobanchaceae			Plant used as medicine (KL Bhutan: [71]).
471	*Oxalis corniculata* L.	Herb	Oxalidaceae	Chari Amilo (Np); Tandi, chatumarak, Kedumarajan (S)	300-2900	**Plant**: Juice used to treat indigestion, diarrhoea, piles, anemia, and eye problems (Ilam: [73, 76]). Chewed raw as appetizer and also checks boils (Sikkim: [11]). Pounded with cumin seeds and taken to cure dysentery and diarrhea. Paste applied on forehead to treat headache. Paste mixed with rhizome of *Drymaria quercifolia* and applied on bone fracture (Jhapa: [66]). **Roots:** Used in infamation (Sikkim: [75]). **Leaves:** Juice taken to cure dysentery (Sikkim: [11], Database) and fever, anemia, and for digestion (Sikkim: Database). **Fruit:** Consumed to cure throat pain (Sikkim: [11]). **Leaves:** Used as vegetable (Ilam: [94]; Jhapa: [92]).
472	*Pandanus nepalensis* St. John	Shrub	Pandanaceae			**Leaves:** Young leaves chewed as breath sweetener. Fresh leaves acts as cockroach repellant. Used to make mats, carry bags, fishing bags and thatching purpose (Ilam: [94]). **Fruit**: Used to make pickle (Ilam: [94]).
473	*Argemone mexicana* L.	Herb	Papaveraceae	Thakal (Np); Dhamoi (S)	150-1400	**Leaves:** Juice applied on cut, wounds, blisters, and burns (Jhapa: [66]). **Latex:** Used in opthalmic infections (Jhapa: [66]). **Roots and leaves:** Paste or juice used in skin disease, constipation, cough and fever (Ilam: [76]).
474	*Corydalis chaerophylla* DC.	Herb	Papaveraceae			**Fruits:** Taken in stomachache (Sikkim: [75]).
475	*Corydalis crispa* Prain	Herb	Papaveraceae			**Whole plant:** Used as medicine (KL Bhutan: [71]).
476	*Corydalis dubia* Prain	Herb	Papaveraceae			**Whole plant:** Used as medicine (KL Bhutan: [71]).
477	*Dicentra scandens* (D. Don) Walp.	Climber	Papaveraceae		2200-3000	**Roots:** Crushed and used to stop excessive bleeding in females (Sikkim: [11]).
478	*Hypecoum erectum* L.	Herb	Papaveraceae			**Whole plant:** Used as medicine (KL Bhutan: [71]).
479	*Hypecoum leptocarpum* Hook. f. & Thomson	Herb	Papaveraceae			**Whole plant:** Used as medicine (KL Bhutan: [71]).
480	*Meconopsis grandis* Prain	Herb	Papaveraceae	Upal Ngongpo (Sh, Tb); Upa Gnono (Wl)	3000-5200	**Plant:** Used as fodder for goat (Taplejung: [81, 87]). **Seeds:** Edible (Taplejung: [81, 87]).
481	*Meconopsis horridula* Hook. f. & Thomson	Herb	Papaveraceae			**Whole plant:** Used as medicine (KL Bhutan: [71]).
482	*Meconopsis paniculata* Prain	Herb	Papaveraceae	Kheldar, Langur (Np-Dl); Upa Sepu (Wl)	3000-4400	**Young twigs:** Used as fodder for goat; young stems eaten raw (Taplejung: [81, 87]).
483	*Meconopsis simplicifolia* (D. Don) Walp.	Herb	Papaveraceae	Upal Mentook (Bhu)	3300-4500	**Rhizomes:** Powder or decoction used as tonic in renal complaints (Darjeeling: [49]). **Whole plant:** Used as medicine (KL Bhutan: [71]).
484	*Passiflora foetida* L.	Climber	Passifloraceae	Sano Jhar (Np)	100-1200	**Leaves:** Infusion consumed to cure insomnia, hysteria, epilepsy and as painkiller (Darjeeling: [74]).
485	*Passiflora nepalensis* Wall.	Climber	Passifloraceae	Garendal (Np)		**Roots:** Decoction taken in fever (Darjeeling: [78]).
486	*Sesamum orientale* L.	Shrub	Pedaliaceae	Siwing (Me)	600-2400	**Seeds:** Chewed and applied on skin for sunburns and ringworm (Jhapa: [68]).
487	*Phyllanthus acidus* (L.) Skeels	Tree	Phyllanthaceae	Atummeral (S); Amala (Np)		**Bark:** Infusion taken to cure stomatitis and aphthous (Jhapa: [66]).
488	*Phyllanthus emblica L.	Tree	Phyllanthaceae	Amala (Np); Meral (S); Chhorgengsoi (Sh)	150-1400	**Fruit and leaves:** Juice taken to cure jaundice, dyspepsia, cough, and asthma (Panchthar: [67]; Ilam: [73, 76]). **Fruit:** Edible and also used as incense (KL Bhutan: [70]). Used as medicine (KL Bhutan: [71]). Good for burning sensation of heat and urinary discharge, liver complaint, and eye trouble (Sikkim: Database). Edible (Jhapa: [92]; Sikkim: [11]) and eaten raw to treat cough, dysentery and diarrhea (Sikkim: [11]). Used as tonic (Jhapa: [66]). Used to make pickle (Ilam: [94]). **Flowers and fruit:** Use to extract dye (KL Bhutan: [70]).
489	*Phyllanthus reticulatus* Poir.	Shrub	Phyllanthaceae	Smeldam (S)	400-770	**Leaves:** Paste applied on burns and boils (Jhapa: [66]).
490	*Phyllanthus urinaria* L.	Herb	Phyllanthaceae	Kanthad (S)	770-1700	**Leaves:** Paste applied in wounds and sores (Jhapa: [66]).
491	*Phytolacca acinosa* Roxb.	Herb	Phytolaccaceae	Jaringe Sag, Jarko Sag (Np); Zalmathangru (Sh)	2200-3200	**Roots:** Used as medicine (KL Bhutan: [71]). Decoction used in jaundice (Taplejung: [88]). Juice dropped in the nose to cure sinusitis (Sikkim: [79]). **Leaves:** Effective in high blood pressure (Darjeeling: [74]; Sikkim: [75]). Fresh juice applied on cuts and wounds to stop bleeding and infection (Sikkim: Database). Decoction taken to cure body ache and diarrhea (Sikkim: [11]). **Seeds and leaves:** Used in indigestion and eye problems (Ilam: [73, 76]).
492	*Piper longum L.	Climber	Piperaceae	Chhimpri-gupai (Me)	200-800	**Roots:** Used as anthelminthic, improves appetite, and abdominal pain (Darjeeling: [80]; Sikkim: [89]). Paste given to treat pneumonia in adults (Jhapa: [68]). Powder given to treat cold (Sikkim: [11]). **Fruit:** Used as medicine (KL Bhutan: [71]). Taken to cure diarrhea, dysentery, piles, and leprosy (Darjeeling: [80]). Dried unripe fruits used as alternative and tonic. Ripe fruits, aromatic, stomachic and carminative. Infusion of dried fruit taken in cough (Darjeeling: [78]; Sikkim: [11]) and fever (Darjeeling: [78]). **Seeds:** Powder applied to reduce strains (Darjeeling: [80]).
493	*Piper nigrum* L.	Herb	Piperaceae			**Fruit:** Used as spices and medicine (KL Bhutan: [70]).
494	*Hemiphragma heterophyllum* Wall.	Herb	Plantaginaceae	Kanakmala (Li)	2600-4100	**Fruit:** Juice administered orally to treat sore throat (Sikkim: [79]).
495	*Picrorhiza kurrooa* Royle ex Benth.	Herb	Plantaginaceae	Puti-shing (Dz), Kutki (N)		**Roots:** Used as medicine (KL Bhutan: [71]). Fresh root paste applied to cuts, wounds, bruises, and injuries (Sikkim: [91]).
496	*Plantago asiatica* subsp. *erosa* (Wall.) Z. Yu Li	Herb	Plantaginaceae	Isagbul (Np)	400-3800	**Leaves:** Taken to cure toothache (Sikkim: [75]). Paste applied to treat wounds. **Seeds:** Powder taken to treat diarrhea and dysentery (Sikkim: [11]).
497	*Plantago depressa* Willd.	Herb	Plantaginaceae	Tsa-shokum (Sh)		**Whole plant:** Used as medicine (KL Bhutan: [71]).
498	*Plantago major* L.	Herb	Plantaginaceae	Jibray Jhar (Np)		**Plant:** Decoction taken to cure fever (Darjeeling: [78]). **Leaves:** Juice consumedin the case of throat pain. **Flowers and fruit**: Juice applied on cuts and wounds (Darjeeling: [74]). **Roots:** Paste applied externally on cuts and wounds for quick healing (Sikkim: [84]).
499	*Plumbago zeylanica* L.	Shrub	Plumbaginaceae	Chitu (Np); Chitigni (S)	100-1300	**Roots:** Used in antifertility (Jhapa: [66]). Used to make traditional yeast or starter called *'Marcha'* to make local wine (Darjeeling and Sikkim: [108]). Roasted with potatoand taken orally in jaundice (Darjeeling: [105]).
500	*Arundinaria intermedia* Munro	Herb	Poaceae	Malingo (Np)	2000-3000	**Plant**: Used to make mats, baskets and to construct temporary huts (Sikkim: [89]). **Leaves:** Used as fodder (Ilam: [73]). **Young shoots**: Used as vegetables (Ilam: [73]).
501	*Arundinaria maling* Gamble	Herb	Poaceae	Himalayan bamboo (Eg); Malingo (Np)	1500-3500	**Young shoots:** Cooked as vegetable or pickled and eaten (Ilam: [94]; KL Nepal: [83]). **Leaves**: Used as fodder (KL Nepal: [83]). Mixed with black pepper and feed with little salt to cattle to treat diarrhea and dysentery (Sikkim: [84]). **Roots:** Ash mixed with mustard oil and paste applied externally on ringworm in cattles (Sikkim: [84]).
502	*Arundinaria* sp.	Herb	Poaceae	Bans (Np)		**Shoots:** Used for fencing and to make food and drink containers, hats arrows and quivers. Young shoots edible (KL Bhutan: [100]). **Leaves:** Used as fodder (KL Bhutan: [100]).
503	*Bambusa nutans* Wall. ex Munro	Herb	Poaceae	Mala Bans (Np)	700-1700	**Plant:** Used to support prayer flags by Buddhist (Sikkim: [89]).
504	*Bambusa* sp.	Herb	Poaceae	Bans (Np)		**Shoots:** Edible (KL Bhutan: [70]). Split and woven mats (KL Bhutan: [100]).
505	*Bambusa tulda* Roxb.	Herb	Poaceae	Kanda Bans (Np); Mal Bans (S)		**Plant:** Burnt ash of seedlings applied in leprosy (Jhapa: [66]). **Young shoots:** Chopped and fermented in tight bamboo or glass bottles to make sour pickle locally called 'Mesu' (Darjeeling: [108]; North-east India: [101]).
506	*Coix lachryma-jobi* L.	Herb	Poaceae	Jargedi (S); Bhirkraulo (Np)	900-2100	**Fruit:** Juice drunk regularly for a long time to cure tuberculosis (Jhapa: [66]).
507	*Cymbopogon flexuosus* (Nees ex Steud.) W.Watson	Herb	Poaceae	Solubang (Sh)		**Leaves:** Used to extract essential oil (KL Bhutan: [93]). Extract used as medicine (KL Bhutan: [71]).
508	*Cynodon dactylon* (L.) Pers.	Herb	Poaceae	Dubo (Np); Dubu (S)	100-2000	**Plant:** Juice taken orally to cure scabies, cut, wound, epilepsy, piles (Ilam: [73, 76]), and juice along with garlic and warm mustard oil used to rub on body to relieve bodypain (Jhapa: [66]). **Roots:** Juice used in piles; paste used to heal cuts and wounds (Sikkim: [11]). Freshly prepared juice taken to cure liver cirrhosis (Darjeeling: [52]). **Roots and leaves**: Decoction used to cure diarrhea and dysentery (Sikkim: [11]).
509	*Dendrocalamus hamiltonii* var. *hamiltonii* Nees & Arn. ex Munro	Herb	Poaceae	Choya Bans, Tama (Np)		**Young shoots:** Chopped and fermented in tight bamboo or glass bottles to make sour pickle locally called 'Mesu' (Darjeeling: [101, 108]; Ilam: [94]). Used to make water pipes, water vessels, in house construction and also consumed as vegetable (Sikkim: [89]).
510	*Dendrocalamus hookeri* Munro	Herb	Poaceae	Chilley Bans (Np)		**Shoots:** Used in making baskets and in fencing (Sikkim: [89]).
511	*Dendrocalamus sericeus* Munro	Herb	Poaceae	Bans (Np)		**Shoots:** Used to makedomestic and agricultural implements, such as water containers, baskets, trays, mats, etc. Young shoots edible (KL Bhutan: [100]). **Leaves:** Used as fodder (KL Bhutan: [100]).
512	*Desmostachya bipinnata* (L.) Stapf	Herb	Poaceae	Kush (Np)	170-500	**Roots:** Astringent and galactagogue (Panchthar: [67]).
513	*Drepanostachyum* sp.	Herb	Poaceae	Bans (Np)		**Shoots:** Used to makebaskets, trays, mats, sieves, livestock shelters and temporary dwellings. Yound shoots edible (KL Bhutan: [100]).
514	*Eleusine coracana* (L.) Gaertn.	Herb	Poaceae	Kodo (Np)	1100-2200	**Seeds:** Used to make local wine called 'Jnard' (Darjeeling: [108]).
515	*Eulaliopsis binata* (Retz.) C.E. Hubb.	Herb	Poaceae	Babiyo (Np)	2000-1000	**Plant:** Whole plant dust applied to treat skin allergy (KL Nepal: [53]).
516	*Fargesia grossa* T. P. Yi	Herb	Poaceae			**Culms:** Used to make mats, bags and baskets (Bhutan: [99]).
517	*Himalayacalamus hookerianus* (Munro) Stapleton	Herb	Poaceae	Pareng (Np)		**Plant:** Used to make basket and hut construction (Darjeeling: [89]). **Young shoots:** Consumed as vegetable (Darjeeling: [89]).
518	*Imperata cylindrica* (L.) P. Beauvois	Herb	Poaceae	Siru (Np)	200-1200	**Roots:** Juice taken in diarrhoea and dysentery (Ilam: [73]), taken during cough, cold and fever (Darjeeling: [91]; Sikkim: [79]). A piece of root tied in hair to ease delivery (Jhapa: [66]). Paste applied on boils (Darjeeling: [91]; Sikkim: [79]).
519	*Phyllostachys edulis* (Carrière) J. Houzeau	Herb	Poaceae	Kattabans (Np)	1000-1600	**Shoots:** Young shoots used to make curry and pickles (Ilam: [94]).
520	*Saccharum spontaneum* L.	Herb	Poaceae	Kash (Np); Kashim (S)		**Roots:** Extract taken to kill intestinal worms and also relieving for fever and bodyache (Jhapa: [66]).
521	*Thamnocalamus* sp.	Herb	Poaceae	Bans (Np)		**Shoots:** Used forroofing, mats and fencing (KL Bhutan: [100]).
522	*Thysanolaena maxima* (Roxb.) Kuntze	Herb	Poaceae	Amriso (Np)	100-2000	**Roots:** Extract used in boils and worms (Ilam: [73, 76, 77]; Sikkim: [79]). Paste applied on boils (Darjeeling: [52]). Decoction of young roots taken in case of bronchial problem (Darjeeling: [49]). **Leaves:** Used as abortifacient (Panchthar: [67]). **Flowers:** Poultic of young flowers used in rheumatic pain and skin swelling (Darjeeling: [49]). **Inflorescence:** Used to make broom (KL Bhutan: [100]).
523	*Polygala arillata* Buch.-Ham. ex D. Don	Shrub	Polygalaceae	Yellow milkwort, Red eye (Eg); Cleem-soon-creem, Karima, Marcha, Michepnor-kung (Np)	600-1800	**Roots:** Juice taken for its laxatic and emetic actions (Sikkim: [85]). **Plant:** Used to make *marcha* (Darjeeling: [80]).
524	*Bistorta vivipara* (L.) Gray	Herb	Polygonaceae	Pantsa Ramba, Rambu (Sh); Pangram, Rmbu Godpa (Tb)	3300-5000	**Plant:** Used as fodder (Taplejung: [87]).
525	*Fagopyrum esculentum* Moench	Herb	Polygonaceae	Phapar (Li)	1800-4100	**Leaves:** Juice taken orally during stomachache and gastritis (Sikkim: [79]).
526	*Oxyria digyna* (L.) Hill	Herb	Polygonaceae	Mountain Sorrel (Eg); Boke (Np)	2400-5000	**Plant:** Used as fodder (Taplejung: [87]).
527	*Persicaria barbata* (L.) H. Hara	Herb	Polygonaceae	Bish (Np); Bareputuli (Me)	200-2500	**Leaves:** Paste with cloves of *Allium sativum* applied on ringworm after slightly scouring the wound by warm salt-water (Jhapa: [68]).
528	*Polygonum coriaceum*Sam.	Herb	Polygonaceae			**Roots:** Used as medicine (KL Bhutan: [71]).
529	*Polygonum molle* D. Don.	Herb	Polygonaceae	Kandyeo-pam,, Patusua, Thotne, Tuknu (Np)	1300-4000	**Plant:** Juice taken to treat diarrhea (Ilam: [76, 77]; Sikkim: [85]). Used as astringent (Sikkim: Database). **Young shoots**: Used for curry (Ilam: [94]) and pickle (Ilam: [73, 94]; Sikkim: Database). Useful in diarrhoea (Ilam: [73]). **Leaves:** Used as fodder (Taplejung: [81, 87]).
530	*Polygonum runcinatum* Buch.-Ham. ex D. Don	Herb	Polygonaceae	Ratnaulo (Np)	1600-3800	**Whole plant:** Used to make vegetable (Ilam: [94]).
531	*Polygonum* sp.	Herb	Polygonaceae			**Roots:** Used as medicine (KL Bhutan: [71]).
532	*Polygonum verticillatum* Biroli ex Colla	Herb	Polygonaceae			**Roots:** Used as medicine (KL Bhutan: [71]).
533	*Polygonum viviparum* L.	Herb	Polygonaceae	Ratnaula (Np)	1200-4500	**Roots:** Juice boiled with water and given in fever (Sikkim: [11], Database), recommended for jaundice (Sikkim: Database) and stomach trouble (Sikkim: [11], Database). **Leaves:** Paste applied on insect bite (Darjeeling: [74]).
534	*Rheum acuminatum* Hook. f. & Thoms. ex Hook.	Herb	Polygonaceae	Padamchal (Np); Bokya (Np-Dl); Khokkim (Np-Tp); Chhurta, Khokkim, Kyojung (Sh); Chum Tsa (Tb)	3200-4300	**Roots:** Used as medicine (KL Bhutan: [71]). Herbal tea prepared from rootstock used to treat body pain; paste applied on forehead during severe headache (Sikkim: [79]). **Petioles:** Eaten raw (Taplejung: [87]) and also as appetizer (Sikkim: [79]). **Leaves:** Juice from the shoot portion taken in dysentery and intestinal problems (Sikkim: [79]).
535	*Rheum australe D. Don	Herb	Polygonaceae	Himalayan Rhubarb (Eg); Chuk, Chulthi Amilo, Mire Chuk, Padamchal (Np); Khokkim (Np-Tp); Chhulama, Chhoyoma, Churcha (Sh); Amlaparni, Pitamulika (Sn); Chhucha (Wl)	3200-4200	**Roots:** Dried and used as tea (Darjeeling: [80]). Taken in constipation, skin eruption, and liver complaints (Sikkim: [75]). Decoction used as tea during internal injuries, body pain, and for blood purification. Rootstocks also yields dye for coloring wool (Taplejung: [81, 87]; KL Nepal: [83]; Sikkim: Database). Rhizome powder taken in diarrhoea, dyspepsia, constipation and ulcer (Ilam: [76]). **Petiole:** Young petioles and leaves used to cure cuts and wounds, sprains and bodyache (Sikkim: [90]). Fresh petioles eaten raw (Taplejung: [81, 87]; KL Nepal: [83]; Sikkim: Database).
536	*Rheum nobile* Hook. f. & Thoms.	Herb	Polygonaceae	Padamchal (Np); Kenjo, Chhulama (Sh, Wl); Chuka (Dz)	3600-5000	**Roots:** Used as medicine (KL Bhutan: [71]).Effective in ulcer, bronchitis, and fever (Sikkim: [75]). Boiled and paste with flour and Tibetan salt applied on the fractured area. Boiled with rhizome of *Begonia* and paste applied externally on the fractured bone (Taplejung: [81, 87]). Decoction or infusion aken to treat rheumatism, arthritis, heart complaints and as tonic after delivery (Darjeeling: [49]). Used as anti emetic, diuretic, laxative, heating potency and for swelling (Darjeeling: [98]). **Plant:** Consumed as vegetable or pickle (Darjeeling: [80, 98]).
537	*Rumex nepalensis* Spreng.	Herb	Polygonaceae	Halhale (Np)	1200-4200	**Roots:** Extract used orally in hepatitis and loss of hair (Darjeeling: [80]; Sikkim: [11, 89], Database) and as dyes (Sikkim: Database). Infusion taken to treat ulcer, cuts, and sprains (Darjeeling: [91]; Ilam: [73, 76, 77]). **Shoots:** Used in skin diseases (Sikkim: [75]). **Leaves and shoots:** Juice applied on woundto heal (Darjeeling: [86]; Sikkim: [11]). **Leaves:** Cooked and taken as vegetable (Ilam: [94]). Leaf and root paste taken in stomachache and applied on wound (KL Nepal: [53]).
538	*Portulaca oleracea* L.	Herb	Portulacaceae	Kulfa Saag (Np)	300-1500	**Young plants:** Used as vegetable (Jhapa: [92]).
539	*Androsace hookeriana* Klatt	Herb	Primulaceae			**Roots:** Used as medicine (KL Bhutan: [71]).
540	*Primula sikkimensis* Hook.	Herb	Primulaceae			**Whole plant:** Used as medicine (KL Bhutan: [71]).
541	*Helicia nilagirica* Bedd.	Tree	Proteaceae			**Leaves:** Use to extract dye (KL Bhutan: [70]).
542	*Aconitum ferox* Wall. exSer.	Herb	Ranunculaceae	Indian Aconite (Eg); Bikh, Bish, Atisingua bish, Seto bikhma (Np)	2100-3800	Plant used as expectorant, febrifuge and in diabetes (Sikkim: [89]). **Rhizomes:** Used in malaria and other fevers, abdominal pains, diarrhoea and indigestion particularly for children (Darjeeling: [69]). Given in cough, skin disease (Sikkim: [11, 85], Database), asthma, leprosy and snakebite (Sikkim: Database). Decoction taken orally to treat abdominal disorders (Darjeeling and Sikkim: [69]; Sikkim: [85]; KL Nepal: [53]). Dried rhizome juice taken as antipyretic agent (Darjeeling: [78]; Sikkim: [11, 90]). Dried rhizome chewed in food poisoning, cold and cough (Sikkim: [79]). Chewed in headache, powder taken orally to cure nervousness and heart problems (Ilam: [76]; KL Nepal: [53]).
543	*Aconitum heterophyllum* Wall. ex Royle	Herb	Ranunculaceae	Bikh, Atish (Np)	3200-3700	**Rhizomes:** Used as bitter tonic and antidote for snakebite (Darjeeling: [69]); taken orally against food poisoning (Darjeeling: [52]). Used as anti-fertility agent, tonic, stomachic, anti-periodic and given in hysteria, piles and throat diseases (Sikkim: Database). Dried rhizomes taken to cure body ache (Sikkim: [11]; Darjeeling: [52]), fever, cold, cough and nose discharge (Sikkim: [11, 91]).
544	*Aconitum lethale* Griff.	Herb	Ranunculaceae	Manchhen (Sh), Tsenduk (Dz)		**Roots:** Used as medicine (KL Bhutan: [71]).
545	*Aconitum naviculare* (Brühl) Stapf	Herb	Ranunculaceae			**Rhizomes:** Used as medicine (KL Bhutan: [71]).
546	*Aconitum orochryseum* Stapf	Herb	Ranunculaceae	Bong-nga (Sh)		**Rhizomes:** Used as medicine (KL Bhutan: [71]).
547	*Aconitum palmatum* D.Don	Herb	Ranunculaceae	Kuphora Bikhma (Li); Lungezee nyin (Lp); Bikhma, Nirmasi (Np); Bongmar, Bongser, Pomar (Sh); Bhungna (Wl)	3500-4500	**Roots:** Powder used to treat fever (Taplejung: [81, 88]; Sikkim: [75]), headache, stomachache (Taplejung: [81, 82, 87, 88]) and rheumatism (Sikkim: [75]).
548	*Aconitum spicatum (Bruhl) Stapf	Herb	Ranunculaceae	Seto Bikhuma (Np)	1800-4200	**Rhizomes:** Used for cholera, rheumatism (KL Nepal: [83]), and also to treat rabies and stomachache (KL Nepal: [53]). Paste given in diarrhea (Ilam: [73]). Buti (*Amulet*) prepared with tuber and tied on the body of a baby to keep far from bad spirit (Taplejung: [81]).
549	*Anemone rivularis* Buch.-Ham. ex DC.	Herb	Ranunculaceae			**Fruits:** Used as medicine (KL Bhutan: [71]).
550	*Clematis acuminata* DC.	Climber	Ranunculaceae	Pinasay Lahara (Np)		**Roots:** Given to treat sinusitis (Sikkim: [75]).
551	*Clematis buchananiana* DC.	Climber	Ranunculaceae	Tinpate Lahar, Pinasay Lahara (Np); Maha Gagri (Np-Tp); Bhwaresang, Thaknangjwa (Ri); Pipipma (Sh); Khondro Langdu (Tm); Imong-Karpi (Tb)	1800-3300	**Roots:** Juice or powder used in headache, sinusitis (Taplejung: [87]; Sikkim: [11, 75, 89], Database), half headache and to make fermenting agent (Taplejung: [87]). Fresh roots crushed and used to release effluvium through nose to cure sinusitis and nose blocks (Darjeeling: [52, 74, 80]).
552	*Clematis montana* Buch.-Ham. ex DC.	Climber	Ranunculaceae	Kaneshi Lahara (Np); Lanitokaru (Sh)	300	**Roots:** Taken to cure sinusitis (Sikkim: [75]). **Seeds/flowers:** Used as medicine (KL Bhutan: [71]).
553	*Delphinium brunonianum* Royle	Herb	Ranunculaceae			**Whole plant:** Used as medicine (KL Bhutan: [71]) and incense (KL Bhutan: [70]).
554	*Delphinium cashmerianum* Royle	Herb	Ranunculaceae			**Whole plant:** Used as medicine (KL Bhutan: [71]).
555	*Thalictrum chelidonii* DC.	Herb	Ranunculaceae	Dampatey (Np)	2300-3500	**Roots:** Used as tonic and purgative (Sikkim: [75]).
556	*Thalictrum foliolosum* DC.	Herb	Ranunculaceae	Dampatey (Np)	1300-3400	**Roots:** Used as medicine (KL Bhutan: [71]). Used as tonic and purgative (Sikkim: [75]). Decoction taken in fever (Darjeeling: [78]).
557	*Thalictrum reniforme* Wall.	Herb	Ranunculaceae		2800-3300	**Roots:** Used to treat jaundice (Panchthar: [67]). Paste applied to cheek boils (Sikkim: [11], Database). Decoction used as gargle in case of bad breath and kills worms in stomaching on drinking (Sikkim: [11]).
558	*Gouania leptostachya* DC.	Climber	Rhamnaceae			**Leaves:** Paste applied to cure sores and inflammation (Sikkim: [11]).
559	*Hovenia dulcis* Thunberg	Tree	Rhamnaceae	Coral tree (Eg); Bangikath (Np)	200-1400	**Seed:** Powder taken to get relief from excessive drinking of alcohol (Sikkim: [85]).
560	*Rhamnus napalensis* (Wall.) M. A. Lawson	Shrub	Rhamnaceae	Archal (Np)		**Roots**: Used as purgative (Sikkim: [75]). **Roots and leaves:** Applied on fractured bone (Sikkim: [75]).
561	*Zizyphus mauritiana* Lam.	Tree	Rhamnaceae	Janum (S); Bayer (Np)	200-1200	**Roots, seeds and bark:** Useful in measles and pneumonia (Panchthar: [67]). **Bark:** Extract used in diarrhea; powder used for septic wounds (Jhapa: [66]). **Fruit:** Eaten raw (Jhapa: [92]).
562	*Zizyphus rugosa* Lam.	Shrub	Rhamnaceae	Sekra (S)	150-800	**Fruit:** Eaten raw (Jhapa: [92]).
563	*Zizyphus* spp.	Shrub	Rhamnaceae	Khankarisey, Khanglchalingsay (Sh)		**Fruit:** Edible (KL Bhutan: [70]).
564	*Chaenomeles speciosa* (Sweet) Nakai	Shrub	Rosaceae			**Flowers and fruit:** Use to extract dye (KL Bhutan: [70]).
565	*Cotoneaster microphyllus* Wall. ex Lindl.	Shrub	Rosaceae		2000-5000	**Roots:** Used as astringent (Sikkim: [75]). **Fruit:** Used as medicine (KL Bhutan: [71]).
566	*Docynia indica* (Wall.) Decne.	Tree	Rosaceae	Mayel (Np); Thungchurpu (Sh); Tong (Dz)	2000-3000	**Fruit:** Used to make jam and pickles (Ilam: [94]). Edible (KL Bhutan: [70]).
567	*Duchesnea indica* (Andrews) Focke	Herb	Rosaceae	Bhui Aiselu (Li)	1000-25000	**Leaves:** Paste applied on cut and wounds (Darjeeling: [74]).
568	*Fragaria nubicola* Lindl. ex Lacaita	Herb	Rosaceae	Bhui Aiselu (Li)	1600-4000	**Roots:** Paste applied externally to control bleeding; chewed in cough and cold (Sikkim: [79]). Juice taken in cough and cold, toothache, and high altitude sickness (Darjeeling: [74]). **Fruit:** Ripen fruits cosumed (KL Bhutan: [70]; Ilam: [94]). **Leaves and fruit:** Pounded and administered with lukewarm water to cattle to treat diarrhea and dysentery, also used as diuretic for cattle (Sikkim: [84]).
569	*Potentilla fructicosa* L.	Herb	Rosaceae	Teba, Pangsermendo (Gr-Mn); Pema, Pama, Pang Ser Metog (Km); Bhairung Pate, Jhwani (Np)	2700-4600	**Roots:** Used for breast disease, stomach and lung disorders, and indigestion; also used as incense (KL Nepal: [83]). **Stem:** Used as brooms (Taplejung: [87]). **Leaves and flowers:** Used in making tea (Taplejung: [87]).
570	*Potentilla fulgens* Wall. ex Hook.	Herb	Rosaceae	Bajradanti (Np)	1600-4800	**Plant:** Juice taken to treat stomach trouble, cough, and cold. Decoction given to cattle to regulate fertility (Sikkim: [97]). **Roots:** Powder used to cure toothache and diarrhea (Sikkim: [75]) and pyorrhea. Juice used in throat and tooth infection, peptic ulcer, cough and cold (Ilam: [73, 76]). Root stock used to treat gastric troubles (Panchthar: [67]).
571	*Prinsepia utilis* Royle	Shrub	Rosaceae	Phekray (Np)	1500-2900	**Seed:** Oil consumed (Ilam: [94]).
572	*Prunus cerasoides* D. Don	Tree	Rosaceae	Payun, Aaru Patay (Np)	1300-2400	**Bark:** Crushed and applied on the injuries of cattle (Sikkim: [11, 97]). Decoction taken to treat fever (Darjeeling: [78]). **Bark and stems:** Applied on fractured bone and toothache (Sikkim: [75]). **Fruit:** Edible (Ilam: [94]; Sikkim: [11, 95]).
573	*Pyrus pashia* Buch.-Ham. ex D. Don	Tree	Rosaceae	Mayal (Np)	750-2600	**Fruit and bark:** Taken to cure menstrual disorders (Ilam: [73]). **Fruit:** Extract taken to cure dysentery (Darjeeling: [80]).
574	*Rosa macrophylla* Lindl.	Shrub	Rosaceae	Himalayan Rose (Eg); Jangali gulaf (Np)	1400-3300	**Fruit:** Used as medicine (KL Bhutan: [71]). Used for fever, diarrhea, and bile disorders; edible and also used for making local wine (KL Nepal: [83]).
575	*Rosa sericea* Lindl.	Herb	Rosaceae	Sewa (Gr-Mn); Sewa Metog (Km); Sebimendo (Sh); Segue-Karpo, Seroga, Sewa (Tb); Setokpa (Wl)	2200-4600	**Bark:** Used in cases of poisoning and lymph fluid disorders (KL Nepal: [83]). **Flower and ripe fruit:** Used in liver, bile, wind and lung diseases and menstrual disorders. **Ripe fruit**: Eaten raw (Taplejung: [81]). Believed to have curative effects in headache, liver complaints, and constipation (Sikkim: [75, 79]). **Leaves:** Decoction used to wash wound (Sikkim: [79]). **Flowers:** Used as medicine (KL Bhutan: [71]).
576	*Rubus ellipticus* Sm.	Shrub	Rosaceae	Nepali Raspberry (Eg); Tinsek (Li); Kysyim (Lp); Aiselu, Aeiselu (Np); Ghees, Tudum (Ri); Chhilum, Nyaningma (Sh); Polang (Tm); Kandakari, Gah-Trah (Tb); Chhilum (Wl)	1700-2600	**Roots and bark:** Useful in gastric problem and diarrhea (Darjeeling: [49, 74]; Panchthar: [67]). **Roots:** Juice given in fever (Sikkim: [75]). Paste applied on forehead during severe headache (Sikkim: [11]) and applied to treat wounds (Sikkim: [11]). Root used for abortion (Darjeeling: [52]). **Roots and young shoots:** Used for colic pain (Sikkim: [11]). **Young shoots**: Chewed to stop sudden stomach pain (Darjeeling: [51, 91]; Sikkim: [11]), mouth allergy and paste of tender shoots applied as antidote to snake bite (Taplejung: [87]). Tender leaves and/or young stem shoots pounded and administered to livestock to treat cold and fever (Sikkim: [84]). **Bark:** Crushed and applied on injuries of cattle (Sikkim: [97]). **Fruit:** Used as spices (KL Bhutan: [70]). Edible (Darjeeling: [80]; Ilam: [94]; Sikkim: [95]). Juice used to cure fever and cough; ripe fruits used to make local wine (Taplejung: [88]) and in dysentery and wounds (Ilam: [73]).
577	*Rubus lineatus* Reinw.	Shrub	Rosaceae	Suvuk (L)		**Roots:** Taken in food poisoining (Sikkim: [75]).
578	*Rubus rugosus* Sm.	Shrub	Rosaceae	Jogi Ainselu (Np)	1500	**Root bark:** Used as anthelmintic drug (Panchthar: [67]).
579	*Anthocephalus chinensis* (Lam.) A. Rich. ex Walp.	Tree	Rubiaceae	Kadam (S and Np)	290-800	**Fruit:** Ripe pseudocarps edible (Jhapa: [68]).Consumed during stomachache (Sikkim: [79]). **Seeds:** Roasted and oil consumed (Ilam: [94]).
580	*Anthogonium gracile* Wall. ex Lindl.	Herb	Rubiaceae	Bhui Sunakhari (Np)	1200-2300	**Rhizomes and pseudobulbs:** Paste applied externally to treat bone fracture and dislocation; pseudobulbs crushed to make paste and applied externally to cure boils (Darjeeling: [52]).
581	*Cinchona officinalis* L.	Shrub	Rubiaceae	Sinchona (Np)		**Bark:** Used as remedy for malaria (Sikkim: Database).
582	*Galium aparine* L.	Herb	Rubiaceae	Zangtsi-rokpu (Sh)		**Whole plant:** Used as medicine (KL Bhutan: [71]).
583	*Haldina cordifolia* (Roxburgh) Ridsdale	Tree	Rubiaceae	Pahenley (Np); Sinjo (S)		**Bark:** Decoction used for antipyretic actions (Darjeeling: [78]). **Young shoots:** Decoction applied in eye infections (Jhapa: [66]).
584	*Hedyotis corymbosa* (L.) Lam.	Herb	Rubiaceae	Piriengo (Np)	200-2400	**Plant:** Decoction taken orally to prevent gastric irritability and also act as an anthelmintic (Darjeeling: [74]; Sikkim: [85]).
585	*Hedyotis scandens* Roxb.	Herb	Rubiaceae	Bokre Lahara (Np); Kalhya (L)	400-1800	**Whole plant**: and roots are useful in eye diseases, sprains and boils (Sikkim: [75]). **Roots:** Useful in eye diseases, sprains, and boils (Sikkim: [75]).
586	*Houstonia* spp.	Herb	Rubiaceae	Nombareng (Sh)		**Leaves:** Used as spices (KL Bhutan: [70]).
587	*Meyna pubescens* (Kurz) Robyns	Shrub	Rubiaceae		200	**Young leaves:** Used as vegetable (Jhapa: [92]). **Fruit**: Edible (Jhapa: [92]).
588	*Mussaenda frondosa* L.	Shrub	Rubiaceae	Dhotisara (Np)		**Plant:** Decoction given to treat fever, cough, and asthma (Sikkim: [11]). **Leaves:** Extract given in fever (Darjeeling: [78]). **Roots:** Juice given in jaundice (Darjeeling: [74]).
589	*Mussaenda macrophylla* Wall.	Herb	Rubiaceae	Tungbub (L)		**Roots:** Taken in jaundice (Sikkim: [75]). Juice taken in acidity, diabetes and fever (Ilam: [76]).
590	*Mussaenda treutleri* Stapf	Shrub	Rubiaceae	Tungbub (L)		**Roots:** Given in jaundice (Sikkim: [75]).
591	*Paederia foetida* L.	Climber	Rubiaceae	Barilahara (Np)	300-1800	**Leaves:** Decoction used as medicine (Darjeeling: [78]).
592	*Paederia scandens* (Lour.) Merr.	Shrub	Rubiaceae	Pate biree (Np)	1400	**Stem:** Swollen part of the stem used to treat rheumatism (Panchthar: [67]). **Fruit:** Dried and paste applied around tooth to cure toothache and prevent tooth decay (Sikkim: [11]).
593	*Pavetta indica* L.	Tree	Rubiaceae	White Pavetta (Eg); Kangyphul, Takali, Sundok (Np)		**Roots:** Juice used for purgative action (Sikkim: [85]).
594	*Randia* sp.	Tree	Rubiaceae	Nertingaey (Sh)		**Fruit:** Edible (KL Bhutan: [70]).
595	*Rubia cordifolia* L.	Herb	Rubiaceae	Majito		**Roots and fruit:** Taken in dysentery, uterian pains, and voice complexion (Sikkim: Database). **Stem:** Crushed, mixed with rice wash and given to cattle in post natal problem of cattle (Sikkim: [97]). **Roots:** Used as medicine (KL Bhutan: [71]). Decoction of dried roots taken to treat fever (Darjeeling: [78]). Decoction used in urinary infection; paste applied on skin diseases (Darjeeling: [91]; Sikkim: [11]). Used as dye (Darjeeling: [72]; Sikkim: [11]). Paste applied on forehead to cure headache (Darjeeling: [52]). Paste also used as ointment to treat skin infection; decoction administered to cattle after delivery for quick recovery (Sikkim: [84]).
596	*Rubia manjith Roxb. ex Fleming	Climber	Rubiaceae	Majitho (Np)	1200-2100	**Plant:** Used as dye (Darjeeling: [80]; Sikkim: Database). Decoction used to treat snake bite, desentery, leprosy, skin diseases (Darjeeling: [74]; Ilam: [73, 76]), diabetes, and arthritis (Ilam: [73, 76]). **Roots:** Used as alterative, astringent, and tonic. Used to obtain dye (KL Nepal: [83]). Used as tonic, alternative, astringent (Sikkim: Database). **Stem**: Used in scorpion bite (Sikkim: Database). **Roots and fruit:** Given to treat menstrual disorders (Darjeeling: [49]; Sikkim: [75]).
597	*Rubia wallichiana* Decne.	Climber	Rubiaceae	Vyem (L)	300-2600	**Young shoots:** Taken in jaundice and paralysis (Sikkim: [75]).
598	*Spermadictyon suaveolens* Roxb.	Shrub	Rubiaceae	Ban Champ (Np)		**Roots:** Paste applied externally to relieve from joint pain (Sikkim: [11]).
599	*Tamilnadia uliginosa* (Retz.) Tirveng. & Sastre	Tree	Rubiaceae	Pendra (S); Pidar (Np)	500	**Bark:** Decoction taken orally to treat diarrhea and dysentery (Sikkim: [85]). **Fruit:** Unripe fruits eaten as vegetable (Jhapa: [92]).
600	*Uncaria sessilifructus* Roxb.	Climber	Rubiaceae		200-900	**Root:** Used to treat bone fracture and crack (Panchthar: [67]).
601	*Uncari arhynchophylla* (Miq.) Miq. ex Havil.	Climber	Rubiaceae			**Whole plant:** Used as medicine (KL Bhutan: [71]).
602	*Aegle marmelos* (L.) Correa	Tree	Rutaceae	Sinjo (S); Bel (Np)	600-1100	**Fruit:** Used as medicine (KL Bhutan: [71]). Used in constipation, diarrhea and dysentery (Jhapa: [66]; Panchthar: [67]; Darjeeling: [86]; Sikkim: [79, 91]). Pulp edible (KL Bhutan: [70]; Jhapa: [92]). **Seeds**: Extract taken orally in ulcer (KL Nepal: [53]).
603	*Boenninghausenia albiflora* (Hook.) Rchb. ex Meisn.	Herb	Rutaceae	Likhijari (Np)	600-3300	**Leaves:** Extract applied on the body of cattle to kill lice and flea (Sikkim: [75, 97]).
604	*Citrus medica* L.	Tree	Rutaceae	Bimbira (Li)	700-1200	**Fruit:** Eaten raw in indigestion, vomiting, jaundice and typhoid. Dried skin powder administered in dysentery (Sikkim: [79]).
605	*Citrus reticulata* Blanco	Tree	Rutaceae	Suntala (Li)	600-1800	**Fruit:** Skin dried and paste applied on the face to soften (Sikkim: [79]).
606	*Clausena excavata* Burm. f.	Shrub	Rutaceae		200-500	**Leaves:** Used to remove flies from wounds, sores, and cuts (Jhapa: [66]).
607	*Euodia fraxinifolia* (D.Don) Hook. f.	Tree	Rutaceae	Khanakpa (Np)	1000-2400	**Bark:** Juice given in dysentery and menstrual disorder (Ilam: [73]).
608	*Evodia fraxinifolia* Hook. f.	Tree	Rutaceae	Khanakpa, Kanu (Np)	1200-2100	**Roots:** Powder taken to treat dysentery (Darjeeling: [80]; Sikkim: [85]). **Bark:** Decoction given in fever (Darjeeling: [78]). **Fruit:** Used as antipyretic and in treatment of typhoid; eaten as vegetable (Darjeeling: [80]; Sikkim: [89]). **Seeds:** Used to make chutney and taken with food to improve appetite (Darjeeling: [91]; Sikkim: [79]).
609	*Murraya koenigii* (L.) Spreng.	Tree	Rutaceae			**Leaves:** Used as spices (KL Bhutan: [70]).
610	*Tetradium fraxinifolium* (Hooker) T. G. Hartley	Tree	Rutaceae	Khanakpa (Np); Kanu (L)	700-3000	**Whole plant:** Taken as antipyretic and diuretic (Sikkim: [75]). **Fruit**: Taken as antipyretic and diuretic (Sikkim: [75]).
611	*Zanthoxylum acanthopodium* DC.	Shrub	Rutaceae	Boke timur (Np); Gee (Sh), Thingne (Dz)	1600-2800	**Leaves and seeds:** Used to cure toothache and worms (Darjeeling: [91]; Ilam: [73]). **Branchlets:** Used as toothbrush to relieve toothache (Sikkim: [11]). **Fruit:** Taken in indigestion (Darjeeling: [80]; Sikkim: [74, 75, 79]), used to treat ear diseases, headache, and asthma (Darjeeling: [80]; Sikkim: [89]). Crushed and rubbed on the leg and foot as antileech repellent agent (Darjeeling: [91]; Sikkim: [11]).Used as medicine (KL Bhutan: [71]) and as spices (KL Bhutan: [70]).
612	*Zanthoxylum armatum DC.	Shrub	Rutaceae	Timur (Np)	1100-2500	**Fruit, roots and leaves:** Decoction used to treat toothache, fever, cough, rheumatism, and asthma (Darjeeling: [78]; KL Nepal: [83]; Panchthar: [67]; Ilam: [73, 76, 77]). **Fruit**: Used as spice (KL Nepal: [83]; Panchthar: [67]; Ilam: [73]).
613	*Zanthoxylum budrunga* Wall.	Shrub	Rutaceae			**Fruit:** Used as spices (KL Bhutan: [70]).
614	*Zanthoxylum oxyphyllum* Edgew.	Shrub	Rutaceae	Nepalese Pepper (Eg): Ban Timur, Lekh Timur (Np)	2100-2800	**Fruits:** Pickled and eaten; paste of immature fruit kept between teeth to relieve from toothache (KL Nepal: [83]). **Flowers and fruit**: Extract given to treat pain, tumor, fever, cholera and snake bite (Ilam: [76]).
615	*Salix babylonica* L.	Tree	Salicaceae	Bains (Np)	1400-3600	**Leaves and stem bark:** Infusion taken in fever (Darjeeling: [78]).
616	*Salix calyculata* Hook. f. ex Andersson	Shrub	Salicaceae	Leng Junye, Langma Parma (Km); Lansip (Sh); Langma Chhung (Tb); Langma (Wl)	3600-4500	**Plant:** Used in death ritual. **Leaves and flowers:** Used as incense (Taplejung: [81, 88]).
617	*Osyris wightiana* Wall. Ex Wight	Shrub	Santalaceae	Nundhiki (Np)	1100-2600	**Root bark:** Used to treat body pain and fractured bone (Panchthar: [67]).
618	*Pyrularia edulis* (Wall.) A. DC.	Tree	Santalaceae	Amphi (Np)	1600-1800	**Fruit:** Edible; kernel possess wax which is used for lighting (Sikkim: [89]).
619	*Cardiospermum halicacabum* L.	Climber	Sapindaceae	Fuka fucha (S)	900-1500	**Plant:** Powder mixed with mustard or coconut oil applied to cure sores and wounds (Jhapa: [66]).
620	*Sapindus mukorossi Gaertn.	Tree	Sapindaceae	Ritha (Np)	1000-1200	**Fruit:** Juice used to cure burnt part of the body; also used for epilepsy (Sikkim: Database). Extract applied on head to remove dandruff and lice (Darjeeling and Sikkim: [69]; Sikkim: [11]). Used to extract oil (Ilam: [94]). **Fruit and root bark:** Used as tonic, anthelmintic, purgative, in asthma and piles (Darjeeling and Sikkim: [69]).
621	*Schleichera oleosa* (Lour.) Oken	Tree	Sapindaceae	Baru (S); Kusum (Np)	200-300	**Fruits:** Edible (Jhapa: [92]).
622	*Aesandra butyracea* (Roxb.) Baehni	Tree	Sapotaceae	Chiuri (Np)	200-1500	**Bark and seeds:** Paste applied on fractured bone and in piles (KL Nepal: [53]). **Fruits:** Used to extract oil (KL Bhutan: [93]). Edible ([94]; Sikkim: [11, 47, 89, 95]; KL Bhutan: [70]). Used in rheumatism (Sikkim: Database; [47]). Juice taken to soften the skin (Sikkim: [11]). Oil extract applied on body during winter (Sikkim: [79]). **Leaves**: Used as fodder (Sikkim: [89]).
623	*Diploknem abutyracea* (Roxb.) H.J.Lam	Tree	Sapotaceae	Yika (Dz), Pinsa (Sh)		**Fruits:** Edible (KL Bhutan: [70]).
624	*Madhuca longifolia* (Roxb.) Macbride	Tree	Sapotaceae	Mahuwa (Np)	150-300	**Bark:** Used to treat bone crack (Panchthar: [67]).
625	*Houttuynia cordata* Thunb.	Herb	Saururaceae	Gaytso (Bhu)	1300-2500	**Shoots:** Used as vegetable (Bhutan: [102]). **Leaves:** Decoction given to treat tuberculosis (Darjeeling: [74]).
626	*Astilbe rivularis* Buch.-Ham. ex D. Don	Herb	Saxifragaceae	Tangphung, Tangphung Supari, Toksong Supari (Li); Bansupari, Buriokahti, Budo Okhati, Thulo Ausadi (Np); Thappasid (Ri); Tongsergugay (Sh),	2000-3600	**Roots:** Used as medicine (KL Bhutan: [71]). Taken as tonic for post natal women (Sikkim: [75]). Chewed to relief from pain (Darjeeling: [80]; Sikkim: [89]). Paste applied to treat wound and juice used in fever, dysentery, back pain, waist pain, and throat pain (Taplejung: [82, 87, 88]) body ache, bleeding at pre and post pregnancy (Darjeeling: [52, 74]; Ilam: [73, 76, 77]; Sikkim: [69]). Powder taken in jaundice (Sikkim: [69]). **Roots and leaves:** Taken in diarrhea, dysentery, and as blood purifier (Sikkim: Database). **Leaves:** Chewed raw in toothache (Sikkim: [49, 79]).
627	*Bergenia ciliata (Haw.) Sternb.	Herb	Saxifragaceae	Rockfoil (Eg); Pakhanved (Np); Sallipat, Salpari, Simtadi (Np-Dl); Sediwakthosida (Ri); Pashanaveda (Sn); Hyoma, Kopsokpa, Silviro (Sh); Bhramhendo (Tm)	900-3600	**Roots:** Used as medicine (KL Bhutan: [71]). Used as analgesic and in piles, heart diseases (Darjeeling: [80, 89]), and spleen enlargment (Darjeeling: [80]; Sikkim: [69]).Juice taken as tonic (Sikkim: [75]) and used to treat wound, boils, diarrhea (Taplejung: [87]; Sikkim: [11, 75], Database; Panchthar: [67]) sinusitis, body pain, cough and cold, eye infection, dysentery, abdominal pain, fever, and to check bleeding during menstruation (Taplejung: [87, 88]; Panchthar: [67]). Crushed and tied around the factured bone (Darjeeling: [80]), also used with root of *Bergenia ciliata*, *Kaempferia rotunta,* and *Viscum articulatum* for the same (Sikkim: [79]). Paste given to treat dysentery (Sikkim: [74, 91]); also applied on burns and cuts; dried rhizome used as astringent, given in ulcers and tuberculosis (Sikkim: [69]). Crushed and extract given to cattle to treat diarrhea and dysentery (Darjeeling: [86]; Sikkim: [84]).
628	*Bergenia purpurascens* (Hook. f. & Thomson) Engl.	Herb	Saxifragaceae	Pakhanved (Np); Chhurcha, Kopsyokpa (Sh); Gadur, Ligadur (Tb)	3800-4700	**Roots:** Paste used in wound, bodyache (Taplejung: [82, 87]; Darjeeling: [80, 89]) and bone fracture (Taplejung: [82]). Decoction used in fever, diarrhea (Taplejung: [87]; Sikkim: [75]), knee ache, and loss of eye sight (Taplejung: [87]). Dried roots used as substitute of tea by high altitude dwellers to get relief from bodyache (Sikkim: [79]).
629	*Chrysosplenium carnosum* Hook. f. & Thomson	Herb	Saxifragaceae	Churtsa, Simjhar (Sh); Ya Ki Ma, Ser Ya Ki Ma (Tb)	3800-5500	**Whole plant:** Used as medicine (KL Bhutan: [71]). **Leaves and stem:** Used in common fever and typhoid fever (Taplejung: [82]).
630	*Neopicrorhiza scrophulariiflora (Pennell) Hong	Herb	Saxifragaceae	Gorki, Gurki (Gr-Mn); Katuki, Kutki (Np); Katuko (Np-Dl); Katukaa (Sn); Hogling, Hunglen (Sh, Wl)	3500-4800	**Rhizomes:** Used for bile disease, eye diseases, and gastritis (KL Nepal: [83]). Decoction used to treat cold and cough, throat pain, fever, bodyache, and high blood pressure (Darjeeling: [49]; Taplejung: [81, 87, 88]). Taken in cold, fever and snake bite (Darjeeling: [52]). Used as bitter tonic, laxative, stomachic, effective in dropsy and scropion stings (Sikkim: [69]). Effective in diseases of liver and spleen including jaundice and anaemia (Sikkim: [69]).
631	*Saxifraga mucronulata* Royle	Herb	Saxifragaceae			**Whole plant:** Used as medicine (KL Bhutan: [71]).
632	*Schisandra neglecta* A. C. Sm.	Climber	Schisandraceae	Sighatta Lahara (Np)		**Fruit:** Used as tonic and given in insomnia (Sikkim: [75]).
633	*Digitalis purpurea* L.	Herb	Scrophulariaceae	Fox glove (Np)		**Leaves:** Used as heart tonic and cardiac stimulant (Sikkim: Database).
634	*Lancea tibetica* Hook. f. & Thomson	Herb	Scrophulariaceae			**Whole plant:** Used as medicine (KL Bhutan: [71]).
635	*Scoparia dulcis* L.	Herb	Scrophulariaceae	Chineebimfang (Me)	100-1200	**Plant:** Decoction used in eye troubles (Jhapa: [66]). **Leaves:** Extract drunk and also applied externally on the body as a remedy for the babies weeping all days (Jhapa: [68]). Young leaves taken in diabetes (Sikkim: [75]). **Roots:** Decoction taken orally in diarrhea and dysentery (Jhapa: [66]).
636	*Smilax zeylanica* L.	Climber	Smilacaceae	Kukur Dainey (Np)	150-1500	**Plant:** Used in Urinary complaints and dysentery. **Roots:** Taken as tonic (Sikkim: Database).
637	*Anisodus luridus* Link ex Spreng.	Herb	Solanaceae	Langthang, Longtankobu (Wl)	2300-4000	**Seeds:** Used to treat toothache (Taplejung: [81]). **Fruit**: Used as medicine (KL Bhutan: [71]).
638	*Datura metel* L.	Herb	Solanaceae	Dhaturo, Dhontrey (Np)	300-1200	**Seeds:** Powder used in skin diseases in cattle (Sikkim: [97]; Ilam: [73, 76]), dandruff, leprosy, ulcer, and fever (Ilam: [73, 76]). Burned and smoke inhaled in chronic asthmatic fits (Sikkim: [79]). **Leaves:** Crushed leaf applied on insect bite of cattle (Sikkim: [97]). Burnt and smoke inhaled to treat asthma (Sikkim: [11]). Warmed leaves placed on joint to reduce the pain and swelling (Sikkim: [79]). **Fruit:** Used as medicine (KL Bhutan: [71]). Smoked for toothache; powdered and mixed with warm mustard oil to treat earache (Jhapa: [66]). Taken in mad dog bites (Sikkim: [79]).
639	*Datura stramonium* L.	Shrub	Solanaceae	Gofatdothrabithai (Me)	200-2200	**Seeds:** Used as medicine (KL Bhutan: [71]).Used to treat scabies (Jhapa: [68]). Used as narcotic and also to treat dandruff and hairfall (Panchthar: [67]).Given orally against rabies, nervousness, nausea and hysteria (Darjeeling: [52]).
640	*Hyoscyamus* sp.	Shrub	Solanaceae			**Fruit/Whole plant:** Used as medicine (KL Bhutan: [71]).
641	*Mandragora officinarum* L.	Herb	Solanaceae			**Roots:** Used as medicine (KL Bhutan: [71]).
642	*Physalis minima* L., Herb		Solanaceae	Jangali phokphokey (Np)		**Fruit:** Used as tonic, diuretic, laxative, and useful in inflammations (Sikkim: Database).
643	*Solanum aculeatissimum* Jacq.	Herb	Solanaceae	Goglang (Me); Hinje, Bhingakheta (S)	1600	**Roots and fruit:** Extraction taken in cold and bronchitis. **Fruit:** Smoked for relieving toothache (Jhapa: [66]). **Seeds:** Boiled and vapour taken through mouth to kill germs of teeth (Jhapa: [68]).
644	*Solanum myriacanthum* Dunal	Herb	Solanaceae			**Seeds:** Smoke directed to the infected tooth to cure toothache and tooth decay (Sikkim: [11]).
645	*Solanum nigrum* L.	Herb	Solanaceae	Sano bini (Np)	900-2900	**Aerial parts:** Used as sedative (Darjeeling: [74]). **Roots, leaves and fruit:** Used in the treatment of leucoderma, dysentery, vomiting, asthma, bronchitis, fever, and urinary discharge (Sikkim: Database). **Fruit:** Edible (Ilam: [94]; Jhapa: [92]).
646	*Solanum* sp.	Shrub	Solanaceae	Khalanji (Sh.)		**Fruit:** Edible (KL Bhutan: [70]).
647	*Solanum surattense* Burm. f.	Shrub	Solanaceae	Kalchudo (Np); rangani kate (S)		**Roots:** Grounded with *Alternanthera sessilis and* given in urinary troubles (Jhapa: [66]).
648	*Solanum torvum* Sw.	Shrub	Solanaceae	Henje (S); Thulo bini (Np)	250-750	**Fruit:** Used as vegetables and pickle (Jhapa: [92]). Fried and eaten to cure cold and cough (Jhapa: [66]). **Leaves:** Smooked to treat toothache (Darjeeling: [74]).
649	*Melochia corchorifolia* L.	Herb	Sterculiaceae	Thuik (S)	200-1300	**Young leaves:** Eaten as vegetable (Jhapa: [66]).
650	*Pterospermum acerifolium* (L.) Willd.	Tree	Sterculiaceae	Hattipaila, Numbong (Np)	450-600	**Flowers:** Juice taken to treat peptic ulcer (Sikkim: [85]).
651	*Punica granatum* L.	Tree	Sterculiaceae	Darim (Np)		**Unripe fruit:** Useful in vomiting, fever, heart diseases, sore throat, diarrhea and dysentery (Sikkim: Database). **Fruit/seeds:** Used as medicine (KL Bhutan: [71]).
652	*Symplocos lucida* (Thunberg) Siebold & Zuccarini	Tree	Symplocaceae	Kharane (Np)	500-2600	**Seeds:** Powder applied against spider sting (Sikkim: [75]). In the past people used to extract oil for cooking from the seed (Sikkim: [89]).
653	*Symplocos paniculata* (Thunb.) Miq.	Shrub	Symplocaceae	Pangtsi (Dz)		**Fruit:** Used as medicine (KL Bhutan: [71]), Edible (KL Bhutan: [70]). **Seeds:** Used to extract oil (KL Bhutan: [93]).
654	*Symplocos* sp.	Shrub	Symplocaceae			**Leaves:** Use to extract dye (KL Bhutan: [70]).
655	*Myricaria rosea* W. W. Smith	Shrub	Tamaricaceae	Angmeo (Gr); Thrishing (Km); Chhusin-Ghumbu, Hunbu (Sh, Wi); Hunbu Chungwa (Tb).	3300-4500	**Whole plant:** Used as medicine (KL Bhutan: [71]).**Leaves and flowers**: Used for incense (Taplejung: [87, 88]). **Stem**: Used to treat cough and cold (Taplejung: [87, 88]).
656	*Schima wallichii* (DC.) Korth.	Tree	Theaceae	Chilone (Np); Sakriphal (S)	900-2100	**Bark:** Crushed with common salt and used as vermicide in cattle (Sikkim: [97]). Rubbed on caterpillar infected portion to remove hair (Sikkim: [11]). Pounded and given to cure fever and stomach pain, and also applied in bone fracture and sprain (Jhapa: [66]). Bark paste with the bark of *Bridelia retusa* applied externally on deep cuts and wounds (Sikkim: [79]).
657	*Aquilaria malaccensis* Lam.	Tree	Thymelaeaceae	Aagurushing (Dz/Sh/T)		**Stem:** Heartwood used as medicine (KL Bhutan: [71]) and incense (KL Bhutan: [70]).
658	*Daphne bholua* Buch.-Ham.ex D.Don	Shrub	Thymelaeaceae	Tunguma (Li); Baruwa, Kagaj Pate, Lokta, Seto Baruwa, Seto Lokta, Sikre (Np); Sugumendo, Da Mendok (Sh); Da Mendok (Wi).	2000-4000	**Roots and bark:** Used to treat intestinal worms; decoction used in fever (Taplejung: [87]). **Bark**: Used in the manufacture of handmade paper (KL Nepal: [83]; KL Bhutan: [93]).
659	*Daphne papyracea* Wall. ex Steud	Shrub	Thymelaeaceae	Tunguma (Li); Aule Lokti, Dangma, Jhapre Lokta, Kagatey, Kalo Baruwa (Np); Khultum (Ri)	1500-2400	**Roots:** Used for intestinal troubles (Sikkim: Database). Decoction given in food poisoning (Sikkim: [79]; Darjeeling: [91]; KL Nepal: [83]). Used as a substitute of *Aconitum* sp. (Sikkim: [79]). **Bark**: Decoction given to treat fever (Sikkim: Database). Juice used to treat intestinal worms and used as anti-leech agent; powder cause nasal irritation (Taplejung: [87]). Used to make handmade paper (KL Nepal: [83]; Sikkim: [11]). **Leaves:** Given to baby goat during diarrhea and fever; stalks used to make locally used mats (Sikkim: [11]).
660	*Daphne* sp.	Shrub	Thymelaeaceae			**Inner wood:** Used as incense (KL Bhutan: [70]).
661	*Edgeworthia gardneri* (Wall.) Meisn.	Shrub	Thymelaeaceae	Nepalese paper bush (Eg); Argeli, Argaily (Np)	1500-3000	**Bark:** Used to make paper (KL Bhutan: [93], KL Nepal: [83]; Sikkim: [72]; Database). **Bark:** Used as fish poison (Sikkim: Database).
662	*Trapa bispinosa* Roxb.	Herb	Trapaceae	Paniphalz (S); Paniphal singara (Np).	200-600	**Fruit:** Edible (Jhapa: [92]).
663	*Trema orientalis* (L). Blume	Tree	Ulmaceae	Sitaundu (S); Khari (Np)	1000-1200	**Fruit:** Edible (Jhapa: [92]).
664	*Dendrocnide sinuata* (Blume) Chew	Shrub	Urticaceae	Morange (Np)	200-1000	**Roots:** Powder taken to stop vomitting and diarrhea (Jhapa: [66]).
665	*Elatostema lineolatum* Wight	Shrub	Urticaceae	Damroo (Bhu)	200-1800	**Young shoots:** Used as vegetable (KL Bhutan: [70, 102]).
666	*Elatostema platyphyllum* Wedd.	Shrub	Urticaceae	Gagleto (Li)	700-1900	**Shoots:** Young shoots consumed as vegetable in gastritis (Sikkim: [79]). **Stems and Leaves:** Edible (KL Bhutan: [70]).
667	*Girardinia diversifolia* (Link) Friis	Herb	Urticaceae	Himalayan Nettle (Eg); Mayu Sagi (Li); Kuju (Lp); Allo (Np); Ptale (Ri)	1700-3000	**Roots:** Juice taken in constipation (Ilam: [73, 76]). **Young shoots:** Eaten as green vegetable (Ilam: [94]; Taplejung: [87]; KL Nepal: [83]). Used as substitute for *dal* which is good for diabetes (Darjeeling: [80]). **Leaves:** Paste given in headache and joint pain (Ilam: [76]). **Flowers:** Taken to treat blood pressure (Sikkim: [75]).
668	*Girardinia palmata* (Forssk.) Gaudich.	Shrub	Urticaceae	Zochha (Dz); Gomjazu (Sh);		**Inflorescence:** Edible (KL Bhutan: [70]).
669	*Girardinia* sp.	Herb	Urticaceae	Sissnu (Np)		**Bark:** Used to make ropes (KL Bhutan: [100]).
670	*Laportea terminalis* Wight	Herb	Urticaceae	Sishnu (Np); Sorong (L)	1900-3300	**Leaves and flowers:** Used in blood pressure complaints (Sikkim: [75]).
671	*Urtica dioica* L.	Herb	Urticaceae	Stinging Nettle (Eg); Polo (Gr, Tm); Sagi (Li); Sisnu (Np); Sajilim, Sokhima (Ri); Sadukpa (Wl)	1000-4000	**Plant:** Juice used to treat jaundice, toothache, and hematuria (Ilam: [73, 76]). **Roots:** Paste applied as antidote to dog bite and on minor fractures. **Young shoots:** Taken as vegetables (Darjeeling: [80]; Taplejung: [88]; Sikkim: [11], Database). Young shoots and inflorescences cooked and given in hypertension (Darjeeling: [52]). **Leaves:** Cooked and taken to maintain blood pressure (Darjeeling: [80]; Sikkim: [89]).
672	*Nardostachys grandiflora DC.	Herb	Valerianaceae	Spikenard (Eng); Pagbon, Pangbo, Pangbwang (Li); Pangpay (Dz/Sh); Bhulte, Bhutle, Jatamasi (Np); Bhultya (Np-Dl); Ngorochi (Ri); Jatamansi (Sn); Pangboe (Wl)	3200-5300	**Whole plant and roots:** Useful in skin diseases, leprosy, ulcers, and cough. **Roots:** Used for incense and also in wounds, cough, cold, chronic fever, high blood pressure, and stomach diseases (Sikkim: [49]; Taplejung: [87]; KL Nepal: [83]). Infusion taken to treat stomachache and as laxative (Sikkim: [75, 85]). Decoction used in fever (Darjeeling: [49, 78]). Juice or decoction taken in dysentery and constipation (Ilam: [76]). Used as tonic, stimulant, antispasmodic, diuretic and antiflatulent (Sikkim: [69]). Used as medicine (KL Bhutan: [71]) and incense (KL Bhutan: [70]).
673	*Valeriana hardwickii* Wall.	Herb	Valerianaceae	Nakkali Jatamansi (Np)	1200-4000	**Roots:** Given in hysteria, epilepsy, and neurosis (Sikkim: [75]). Used as carminative and stimulant (Sikkim: Database). Extract taken to cure urine trouble (Sikkim: [11]).
674	*Valeriana jatamansii Jones	Herb	Valerianaceae	Indian Valerian (Eg); Nappu (Gr-Mn); Samayo, Sugandhawal (Np); Bhutakesi (Sn); Jaboe (Wl)	1200-3600	**Rhizome:** Used in fever, cold (Taplejung: [82]), headache, eye compliants, sore throat, wounds, and indigestion (KL Nepal: [83]). Dried rhizome also used as incense (KL Nepal: [83]). Juice given in hysteria, epilepsy, cholera, cough, asthma, weakness and hairfall (Ilam: [76]).
675	*Callicarpa arborea* Roxb.	Tree	Verbenaceae	Guahelo (Np)	1000-2500	**Bark**: Juice given to treat fever (Sikkim: Database). **Roots**: Chewed in cases of boils on the gums (Sikkim: Database). **Fruit:** Juice administered to cure fever (Sikkim: [79]).
676	*Callicarpa macrophylla* Vahl	Shrub	Verbenaceae	Guenlo, Sumali (Np); Tichangsa (Che); Dahidhula (Dar); Dahigun (Tha)	300-1500	**Roots:** Used to treat pneumonia (Panchthar: [67]). Decoction drunk to cure bronchitis (Jhapa: [68]). **Bark:** Used in rheumatism and gonorrhea (Sikkim: Database).
677	*Gmelina arborea* Roxb.	Tree	Verbenaceae	Khamari (Np); Kasnar (S)	200-1100	**Bark:** Pounded and taken as antidote for all kinds of poison; also used in dysentery (Jhapa: [66]).
678	*Lantana camara* L.	Shrub	Verbenaceae			**Leaves:** Juice applied in cut to heal; crushed and tied over the sprain to relieve pain (Sikkim: [11, 79]).
679	*Premna serratifolia* L.	Shrub	Verbenaceae	Gineri (Np)		**Roots:** Decoction taken as laxative and carminative (Sikkim: [85]).
680	*Vitex negundo* L.	Shrub	Verbenaceae	Simali (Np); Sindware (S)	200-1400	**Plant:** Juice used to treat fever, ulcer, asthma, and cough (Ilam: [73]). **Leaves:** Used to treat sinusitis and rheumatism (Panchthar: [67]); also used as repellent for insects and bedbugs (Jhapa: [66]). **Stem:** Juice bath taken to treat body swelling, common cold and influenza (Darjeeling: [74]).
681	*Viola biflora* L.	Herb	Violaceae	Ghattey Ghans (Np)	2100-4500	**Roots and flowers:** Used as emetic and antiseptic (Sikkim: [75]).
682	*Viola diffusa* Ging. ex DC.	Herb	Violaceae	Ghattey Ghans (Np)	1400-2000	**Flowers:** Taken to get relief from chest pain (Sikkim: [75])
683	*Viola serpens* Wall.	Herb	Violaceae	Ghattejhar (Np)		**Roots:** Juice taken in fever (Darjeeling: [78]).
684	*Ampelocissus barbata* (Wall.) Planch.	Climber	Vitaceae	Jarila lahara (Li)		**Plant:** Juice given to treat sores in mouth and tongue of milk sucking baby (Sikkim: [79]).
685	*Ampelocissus latifolia* (Roxb.) Planch.	Climber	Vitaceae	Icewar (S)	300-1600	**Fruits:** Edible (Jhapa: [92]).
686	*Ampelocissus sikkimensis* (M. A. Lawson) Planch.	Climber	Vitaceae		1000-2000	**Plant:** Juice used to cure sores in the mouth of an infant and treats foot and mouth disease in cattle (Sikkim: [11]).
687	*Alpinia allughas* (Retz.) Roscoe	Herb	Zingiberaceae	Churampha (Np)	400-600	**Rhizomes:** Extract taken in diabetes and as laxative (KL Nepal: [53])
688	*Amomum subulatum* Roxb.	Herb	Zingiberaceae	Bada alaichi (Li)	500-2000	**Seeds:** Used as medicine (KL Bhutan: [71]). Used in indigestion and vomiting (Ilam: [76]).Decoction used to gargle to treat teeth and gum infection (Sikkim: [79, 91]). Oil applied on eye-lids to allay inflammation of the eye in cattle; paste applied externally as antidote for scorpion-sting and insect bites (Sikkim: [84]).
689	*Caulokaempferia sikkimensis* (King ex Baker) K. Larsen	Herb	Zingiberaceae	Bhuin Champa (Li)		**Bulb:** Poultice from crushed bulbs applied to heal fractured bone and wound (Sikkim: [11]).
690	*Costus speciosus* (Koenig) Sm.	Herb	Zingiberaceae	Bet Lauri (Np); Urat (S)	400-700	**Roots:** Useful in fever, bronchitis, anemia, rheumatism and diabetic (Darjeeling: [78]; Sikkim: [69]) and diabetic (Darjeeling: [51, 78]; Sikkim: [96]).Used as antiinflamatory, stimulant and anthelmintic and also given in urinary stones (Sikkim: [69]). Juice taken before breakfast to cure urinary tract infection; juice mixed with sugar and used to treat veneral disease (Sikkim: [11]). Juice mixed with milk and sugar cubes and drunk to cure sensation of internal heat and stomach inflammation (Jhapa: [66]). Roots of this plant grounded with leaves of *Swertia chirayita* and taken in fever and urinary complaints (Jhapa: [66]). Root mixed with leaves of *Drymeria cordata* androot paste of *Bombax ceiba* and combinely crushed and administered orally to treat urinary disorders (Sikkim: [79]). Rhizome powder given to cattle to treat fever and inflammation (Sikkim: [84]). **Stem**: Extract given in diabetes and cholera (KL Nepal: [53]).
691	*Curcuma angustifolia* Roxb.	Herb	Zingiberaceae	Bakhre Saro (Np); Nauhaine- haldai (Me)	100-1500	**Rhizomes:** Powder used as antiseptic in cuts, wounds and to check bleeding (Jhapa: [68]).
692	*Curcuma aromatica* Salisb.	Herb	Zingiberaceae	Wild turmeric (Eg); Bandhale, Banhaledo, Kalohaledo (Np); Vanharida (Sn)	700-1100	**Rhizomes:** Used in cough and bronchitis (Taplejung: [82]). Also used as appetizer, tonic, carminative and applied to bruises and sprains; powder used as anthelmintic; oil useful in the treatment of early stage of cervix cancer (Sikkim: [69]).
693	*Curcuma caesia* Roxb.	Herb	Zingiberaceae	Kalo haledo (Np)	200-1500	**Rhizomes:** Powder taken to treat leucoderma, piles, bronchitis, and asthma (Ilam: [73, 76]; Sikkim: [69]). Fresh rhizomes eaten raw to expel gas (Sikkim: [11]). Used as stomachic, diuretic, aromatic, stimulant, carminative and to cure sprains and bruishes (Sikkim: [69]).
694	*Curcuma longa* L.	Herb	Zingiberaceae	Hardi (Np); Juung (Sh); Yongka (Dz)		**Rhizomes:** Used as medicine (KL Bhutan: [71]). Paste prepared from powder and applied on the fractured bones (Darjeeling: [52]).
695	*Curcuma zeodaria* Rosc.	Herb	*Zingiberaceae*	Kalo Hardi (Np); Zalmathangru- tsalu (Sh)		**Rhizomes:** Used as medicine (KL Bhutan: [71]).Eatern raw to cure diarrhea, colic and indigestion; paste applied to treat skin diseases (Sikkim: [11]). Decoction taken to get rid from fever (Darjeeling: [78]). Cut into small pieces and administered orally to treat stomach pain, loss of appetite and also used as antidote to food poisonong (Darjeeling: [52]).
696	*Elettaria cardamomum* (L.) Maton	Herb	*Zingiberaceae*			Used as medicine (KL Bhutan: [71]). **Fruit:** Used as spices (KL Bhutan: [70]).
697	*Hedychium spicatum* Sm.	Herb	*Zingiberaceae*	Pankha Phool, Sara (Np)		**Rhizomes:** Used as medicine (KL Bhutan: [71]). Taken to treat diarrhea, vomiting, and asthma (Sikkim: [69, 75]).Used in liver disorders and stomach ailments (Sikkim: [69]).
698	*Kaempferi agalanga* L.	Herb	*Zingiberaceae*	Borangsaga (Sh)		**Rhizomes:** Used as medicine **(**KL Bhutan: [71]).
699	*Kaempferia rotunda* L.	Herb	*Zingiberaceae*	Vuinchampa (Np)	1300-2000	**Plant:** Juice used in gastric problems, tumors, ulcers, wounds (Ilam: [73, 76]) and swelling, and wounds (Ilam: [76]). **Tubers:** Used as bone settlers (Sikkim: [69], Database) and as poultice in fracture, healing fresh wounds, and removes coagulated bloods from the body (Darjeeling: [80]). Paste prepared along with the roots of *Laportea terminalis* and aerial portion of *Viscum album*and applied on bone fracture and dislocated joints (Darjeeling: [52]). Root decoction used analgesic and sedative (Darjeeling: [74]).
700	*Zingiber cassumunar* Roxb.	Herb	*Zingiberaceae*	Banada (Np)		**Leaves and inflorescence:** Given in cough and fever, dyspepsia. Inflorescence heated in fire and sniff against sinusitis (Darjeeling: [69]). **Rhizomes**: Soup taken during stomachache (KL Nepal: [53]).
701	*Zingiber officinale* Rosc.	Herb	*Zingiberaceae*	Aduwa (Np)	100-1800	**Rhizomes:** Used as medicine (KL Bhutan: [71]). Used as laxative, aphrodisiac, carminative, also useful in heart diseases, throat, and asthma (Sikkim: Database). Used as spice; juice and powder used to cure joint pain, headache, cough, and cold (Ilam: [73, 76]).Roasted rhizome chewed with salt during severe dry cough (Sikkim: [79]).
	**Fungi**					
1	*Lycoperdon pyriforme* Schaeff.	Herb (Fungi)	Agaricaceae			**Whole plant:** Used as medicine (KL Bhutan: [71]).
2	*Ophiocordyceps sinensis (Berk.) Sung et al.	Herb	Ophiocordycipitaceae	Yarcha gombuk (Np); Yaartsa-gunbu (Dz)		**Plant:** Used as medicine (KL Bhutan: [71]). Rejuvenates liver, heart and cheeks ageing process and built up immune system (Sikkim: Database). Species has high commercial value (Bhutan: [99]; Sikkim: [98]). Known to treat health complaints like aging, cancer, diabetes, fatigue, heart diseases, lungs and liver ailments (Sikkim: [96]). Taken as aphrodiasic (KL Nepal: [53]).
	**Lichen**					
1	*Usnea logissima* Ach*.*	Herb	Usneaceae	Lichen, Old-man's beard (Eg); Jhyau (Np)	2500-3900	**Thallus:** Useful in wounds, lung, liver, and fever from poisoning; also useful as incense (KL Nepal: [83]).
2	*Usnea sikkimensis* Biswas	Herb	Usneaceae			**Thallus:** Used to bandage surface of wound and skin eruptions; inserted into the nose to stop bleeding (Sikkim: [11]).
	**Algae**					
1	Ulva spp.	Herb (Algae)	Ulvaceae			**Whole plant:** Edible (KL Bhutan: [70]).
	**Pteridophytes**					
1	*Adiantum capillus-veneris* L.	Herb	Adintaceae	Kane Uniu (Np)		**Leaflets:** Decoction taken as antipyretic (Darjeeling: [78]).
2	*Diplazium asperum* Blume	Herb	Athyriaceae		300-1200	**Roots:** Used to cure dysentery (Sikkim: [11]).
3	*Diplazium esculentum* (Retzius) Swart	Herb	Athyriaceae	Ghinki arak (S); Niguro (Np)	100-1200	**Fronds:** Young fronds cooked and eaten as vegetable (Jhapa: [92]; Darjeeling: [78]; Bhutan: [102]).
4	*Diplazium laxifrons* Rosenstock	Herb	Athyriaceae	Nakey (Bhu)	900-1800	**Fronds:** Young fronds cooked and eaten as vegetable (Bhutan: [102]).
5	*Diplazium maximum* (D. Don) C. Christensen	Herb	Athyriaceae	Nakey (Bhu)	900-1800	**Fronds:** Young fronds cooked and eaten as vegetable (Bhutan: [102]).
6	*Dryopteris fragrans* (L.) Schott	Herb	Dryopteridaceae			**Stem:** Used as medicine (KL Bhutan: [71]).
7	*Equisetum diffusum* D. Don	Herb	Equisetaceae	Ankhle (Np)	100-3400	**Plant:** Paste applied on bone fracture, sprains, and in urinary troubles (Ilam: [73]).
8	*Equisetum ramosissimum* subsp. *debile* (Roxb. ex Vaucher) Hauke	Herb	Equisetaceae	Kurkure Jhar (Np); Haalgoda (Me)	1000-2600	**Plant:** Paste mixed with *Drymaria cordata* and applied in bone factures (Jhapa: [68]). **Aerial parts:** Used as clotting agent for wound, nose bleeding, and bleeding of urinary tract (Sikkim: [11]; Database). Paste applied to cure mouth sores (Darjeeling: [74]).
9	*Lycopodium clavatum* L.	Herb	Lycopodiaceae	Naagbeli (Np); Dermusungfon (L)	700-1800	**Plant**: Administered orally for treatment of muscle contraction in cattle (Sikkim: [84]). **Plant and spores:** Taken in rheumatism, pulmonary disorders, and chronic kidney. **Roots and leaves:** Used in treating rheumatism (Sikkim: [75], Database). **Spores:** Given withpaste of strobilus to cure lung and kidney problems and urinary disorders (Ilam: [73, 76, 77]). Also used to treat scabies and ringworms (Panchthar: [67]). Applied directly on the wound (Sikkim: [79]). Cosumed to treat bleeding after childbirth (Darjeeling: [74]).
10	*Nephrolepis cordifolia* (L.) C. Presl	Herb	Nephrolepidaceae	Paniamala (Np)	500-2000	**Tubers:** Juice taken to treat indigestion, fever, cold, and cough (Ilam: [73, 76]). **Fruit**: Edible (Ilam: [73, 76]).
11	*Lepisorus thunbergianus* (Kaulf.) Ching	Herb	Polypodiaceae			**Whole plant:** Used as medicine (KL Bhutan: [71]).
12	*Drynaria quercifolia* (L.) J. Smith	Herb	Polypodiaceae		200-1000	**Rhizomes:** Juice with sugar drunk to cure stomach inflammation and sensation of internal heat of cattle; grounded with *Oxalis corniculata* and applied on bone fracture (Jhapa: [66]).
13	*Aleuritopteris leptolepis* (Fraser-Jenk.) Fraser-Jenk.	Herb	Pteridaceae	Rani sinka (Np)	1000-3000	**Plant:** Juice used in ulcer and stomachache (Ilam: [73]).
14	*Pteris biaurita* L.	Herb	Pteridaceae	Thado unew (Np)	200-1500	**Stem:** Mashed and applied on cuts and wounds to stop bleeding and infection (Sikkim: [11], Database). **Frond:** Juice consumed to treat dysentery (Darjeeling: [74]).
15	*Pteris terminalis* Wallich ex J. Agardh	Herb	Pteridaceae	Nimin Daway (Bhu)	600-2700	**Young fronds:** Cooked as vegetable (KL Bhutan: [102]).
16	*Lygodium flexuosum* (L.) Sw.	Herb	Schizaceae	Bahun Lahara (Np)	1000	**Fronds:** Young fronds soaked in mustard oil and applied externally on affected areas to treat rheumatism and sprains (Darjeeling: [74]).
17	*Sphagnum squarrosum Crome*	Herb	Sphagnaceae			**Plant:** Used as fuel; hunters and graziers use whole moss for dressing wound (Sikkim: [11]).
18	*Tectaria coadunata* (J.Sm.) C.Chr.	Herb	Tectariaceae	Kalo Oonew (Np)	500-2500	**Plant:** Boiled and taken as soup for a week to treat dysentery (Darjeeling: [74]).
	**Gymnosperms**					
1	*Ephedra gerardiana* Wall. ex Stapf	Shrub	Ephedraceae	Hathijor, Somlata (Np); Kagchalo, Kagcharo, Sallejar (Np-DI); Chhe (Sh), Sankhapa (Sh); Chhewong (Sh, WI), Tshe (Dz)	2000-5200	**Plant:** Used as medicine (KL Bhutan: [71]). Raises blood pressure and used to relieve high fever (KL Nepal: [83]; Sikkim: Database) and asthma (Sikkim: Database). Juice used for liver fever, bleeding, and cuts (KL Nepal: [83]. **Fruit**: Used as digestive (KL Nepal: [83]). Ripe fruits taken to get relief from altitude sickness and indigestion (Sikkim: [79]). **Stem**: Powder inhaled to treat headache (Sikkim: [79]).
2	*Cupressus* spp.	Tree	Cupressaceae	Tsendenshing (Sh); Tsendey (Dz)		**Whole plant:** Used as incense (KL Bhutan: [70]).
3	*Juniperus communis* L.	Tree	Cupressaceae	Sukpa (Np)		**Fruits:** Dried and chewed as a nut or decoction taken in fever (Darjeeling: [78]).
4	*Juniperus indica* Bertol.	Tree	Cupressaceae	Black Juniper (Eg); Dhup (Np)	3700-4500	**Leaves and fruits:** Used in kidney disorders, skin and lymph disease, fever, cough, and cold, sores, wounds and paralysis of limbs (KL Nepal: [83]). **Fruit**: Powder put on the glowing red fire coal and the scent smelt to relief from headache and reduce blood pressure. Dried fruit powder mixed with tea or milk to treat cough, cold in high altitude (Sikkim: [79]). **Leaves**: Used as incense (KL Nepal: [83]).
5	*Juniperus recurva* Buch.-Ham. ex D.Don	Shrub	Cupressaceae	Drooping juniper (Eg); Dhupi (Np); Shup (Dz); Shukpashing (Sh)	3300-4600	**Aerial parts:** Used as incense (KL Nepal: [83]). **Leaves:** Used as medicine (KL Bhutan: [71]).
6	*Juniperus* spp.	Shrub	Cupressaceae			Used as incense (KL Bhutan: [70]).
7	*Abies densa* Griff. ex Parker	Tree	Pinaceae	Gobre salla (Np); Dungshi (Dz)	2550-3700	**Leaves:** Used as medicine (KL Bhutan: [71]) and incense (KL Bhutan: [70]). Juice taken orally to relieve from stomach pain and fever (Sikkim: [11]). Extract used in asthma, bronchitis, and stomach trouble (Sikkim: [79, 80, 89]). Extract given to cattle to treat fever and loss of appetite (Sikkim: [84]).
8	*Abies spectabilis* (D.Don) Mirb.	Tree	Pinaceae	Himalayan Silver Fir (Eg); Gobre Salla, Thingre Salla (Np); Thingro (Np-Dl); Talispatra (Sn); Som Chirugpa (Wl)	2400-4400	**Leaves:** Used as incense (Taplejung: [81, 88]). **Leaves and gum:** Used as tonic and also useful in tuberculosis and internal hemorrhage (Sikkim: Database). **Fruit:** Boiled to obtain black ink (Taplejung: [87]).
9	*Cedrus deodara* (Roxb. ex D. Don) G. Don	Tree	Pinaceae	Deodara (Np)		Oil applied externally to treat ringworm (Sikkim: [97]).
10	*Pinus roxburghii* Sarg.	Tree	Pinaceae	Rani Salla (Np); Aang (Li)	1100-2100	**Leaves:** Extract used in hydrocele and also during bone fracture (KL Nepal: [53]). **Resin**: Used as medicine in rheumatism (KL Bhutan: [71, 93]).
11	*Taxus wallichiana Zucc.	Tree	Taxaceae	Eastern Himalayan Yew (Eg); Silingi (Gr-Mn); Dhengre (Li); Lauthsalla (Np); Kandeloti, Lota (Np-Dl)	2100-3500	**Bark:** Used as medicine (KL Bhutan: [71]). Taken as herbal tea, especially to treat piles (Taplejung: [87]; KL Nepal: [104]) and in muscular pain and fever. **Leaves:** Extract used to cure asthma, bronchitis, and other respiratory diseases (KL Nepal: [83]; Ilam: [73, 76]). **Leaves:** Used as medicine (KL Bhutan: [71]). Antispsmodic, used in nervousness, hysteria and epilepsy (Darjeeling: [69]; Sikkim: [106]). Young shoots used in headache, giddiness, diarrhoea and liver disorders (Darjeeling: [69]; KL Nepal: [104]). **Fruits:** Eaten raw (KL Nepal: [83]; Ilam: [73]).

ǂSikkim: Datadase = Medicinal Plant Database of Government of Sikkim:http://www.sikkimforest.gov.in/medicine_main.htm#tp

*National priority herbs of Government of Nepal

*Abbreviations*: *Bh* Bhotia, *Bhu* Bhutanese, *Dz* Dzongkha, *Eg* English, *Gr* Gurung, *Gr-Mn* Gurung of Manang, *Km* Kham, *Li* Limbu, *Lp* Lepcha, *M* Meche, *Np* Nepali, *Np-Dl*Nepali in Dolpo, *Np-Tp* Nepali in Taplejung, *Nw* Newari, *Ri* Rai, *S* Satar, *Sh* Sherpa, *Sh* Sharchop-kha, *Sn* Sanskrit, *Tb* Tibetan, *Tha* Tharu, *Wl* Walung

We listed ailments as mentioned in the publications but we followed the method proposed by Cook [28] to classify plants according to the different ailment categories they used to cure. However, in some cases Cook’s categories were not precise enough and plants were assigned to additional ailment categories. Chi-square (*χ*2) was used to test the null hypothesis that there is no difference in use of NTFPs under various use categories among the three countries in the Kangchenjunga Landscape.

## Results and discussion

### Pattern of publications

**Fig. 2:**
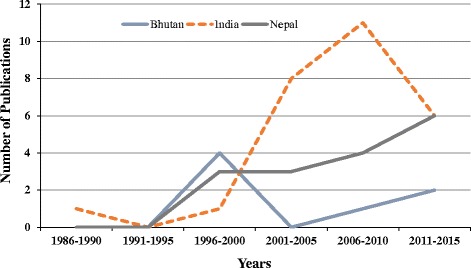
Pattern of publications on NTFP from the Kangchenjunga Landscape

Majority of publications on NTFP were from India (60 %), while 34 % were from Nepal and 6 % from Bhutan. This is quite obvious as Darjeeling and the state of Sikkim in India make up a large part of the KL (56.3 %). The presence of two state level universities and research institutes has made significant contribution to the research in KL India [29]. Except for Bhutan, the species reported in this study were mostly documented through ethnobotanical studies conducted in different parts of the landscape. A few studies were focused on particular ethnic communities whereas most of the studies were on general ethnobotany of the region with mixed ethnic composition. Publication on NTFPs date back to 1988 in India while in Nepal and Bhutan it was started after 1996 (Fig. 2). However, majority of the publications (86 %) were published after 2000. All publications are qualitative in nature.

### Frequency of NTFPs use

We reported on a total of 739 species of NTFPs used by the local people of Kangchenjunga Landscape. Of these, the highest number of NTFPs was documented from India (377 species), followed by Nepal (363) and Bhutan (245). These numbers, however, overlap in terms of distribution. The NTFPs used only in India were 185 species, while this number was 189 for Nepal and 166 for Bhutan.

### Taxonomic diversity and growth habit

**Fig. 3:**
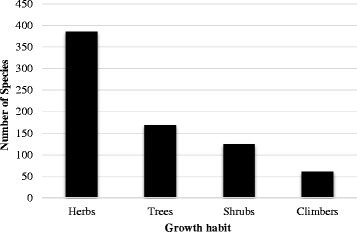
Frequency of NTFPs in different growth habits

Angiosperms were predominant with 705 taxa belonging to 137 families followed by Gymnosperms (10), Pteridophytes (17), Fungi (3), Lichens (2), Bryophyte (1) and Algae (1). Families with the highest number of species used belong to Asteraceae (56 species), Fabaceae (41), Lamiaceae (27), Rubiaceae (24), Poaceae (23), Moraceae (16), Ranunculaceae (16), Rosaceae (15), Zingiberaceae (15), Polygonaceae (14), Ericaceae (13), Rutaceae (13), and Liliaceae (11). NTFPs were distributed into different life forms, with herbs having the most species followed by trees and shrubs (Fig. 3). Pattern of NTFPs used according to different life forms was similar in Bhutan, India and Nepal. Such herbaceous species were mostly medicinal and their extensive use could be because they were frequently found in the forest, and it is believed that the more abundant a plant is the more medicinal virtues it may possess [30].

### Major use categories

People from Kangchenjunga Landscape used NTFPs for 24 different purposes (Table 2). A comparative analysis revealed that the highest number of use categories were reported from the Kangchenjunga Landscape region of India (20 categories) followed by Nepal (18) and Bhutan (14). Despite common occurrence of many species in India and Nepal, use pattern differed greatly in these two countries. Medicinal plants were among the main valuable NTFPs in the landscape. Of the total NTFPs, 334 species were used in traditional medicinal practice in India, whereas 297 species used in Nepal and 176 species used in Bhutan. A considerable number of species were also used as edibles as fruit, vegetables, and pickles in all three countries (Table 1 and Table 2). Fruit and shoots were the most frequently eaten parts.

**Table 2:** Major use categories of NTFPs and frequency of taxa reported from Kangchenjunga Landscape

Use category	Frequency of taxa reported			Total (Kangchenjunga Landscape)
	Bhutan	India	Nepal	
Basket	4	3	–	7
Broom	2	–	1	3
Cigarette wrapper	–	–	1	1
Detergent	–	1	–	1
Dye	12	6	6	23
Edible	62	46	130	191
Fencing	2	1	1	4
Fermentation	–	4	3	4
Fibre	10	4	4	15
Fish poisoning	–	1	1	2
Fodder	3	4	12	19
Fuel	–	1	1	2
Gum	4	–	–	4
Incense	19	3	13	33
Insecticide	–	2	1	3
Medicinal	176	334	297	598
Oil	6	2	7	7
Ornamental	5	1	–	2
Preservator	–	1	–	1
Roofing	2	–	–	2
Ritual	–	3	5	8
Spritual	–	1	1	2
Tea	1	4	4	8
Vetenery medicine	–	27	5	27

The relatively higher number of diversity in wild edible NTFPs in Nepal could be because of higher diversity of ethnic groups living in the lowland Tarai to highland regions. There was a significant difference (*χ*2 = 35.06, df =64, α = 0.05 and 1-α = 83.67) in medicinal plants use pattern in major disease/ailment categories in India and Nepal. These results indicate differences between the traditions of NTFP use in different cultures of India and Nepal. Similar results were also obtained from East Timor [31]. NTFPs use varies from site to site because of the heterogeneity of the community and different traditional practices by ethnic groups [14].

Among 739 species used by the local people, most species were used for a single (550 species) purpose, while fewer were used for two (147) or multiple (42) purposes. Local people were well aware of collecting seasons, mode of collection, and frequency of collection of specific parts of plant species. Medicinal plants such as *Heracleum nepalense* is plucked on the first Tuesday after the *Teej* festival. This practice is known as ‘Harlo’. The people believe that the medicinal plants plucked on that day are extremely effective and potent [32]. Similar practice of harvesting season can be found among the *Amchis* of the Himalaya where they believe that for better medicinal efficacy, specific parts of specific medicinal plants should be collected during specific seasons [33].

### Ailments treated and preparation methods

The use of medicinal plant in treatment of particular ailment and the preparation method were not specified from Bhutan. In India and Nepal, a total of 27 major ailments were reportedly treated with medicinal plants with most species being used to treat multiple ailments (Table 3). Gastro-intestinal disorders; fever; cold, cough and sore throat; musculoskeletal disorders; injuries; dermatological infections; respiratory system disorders; nutritional disorders; and poisoning effects were treated with the highest diversity of medicinal plant species (Table 1 and Table 3). The high diversity of species use in gastro-intestinal disorders could be because of poor sanitation and drinking water quality in the Kangchenjunga Landscape as in many developing contries [34, 35].

**Table 3:** Major disease categories and number of taxa reported from Kangchenjunga Landscape

Disease/ailment/condition category	Number of taxa^a^	
	India	Nepal
Blood system disorders (purification, anaemia, etc.)	9	9
Circulatory system disorders (heart problems, blood pressure, etc.)	23	9
Cough, cold and sore throat	84	54
Dermatological infections (boils, eczema, itch, leucoderma, leprosy, running sore, dropsy, irritant, small pox, chicken pox, skin problems, etc.)	86	63
Diabetes	19	11
Earache, ear irritation	3	5
Fainting and fits	–	2
Fever/malarian fever	83	64
Gastro-intestinal disorders (bile disorder, cholera, colic, constipation, indigestion, diarrhoea, dysentery, dyspepsia, emetic, laxative, liver disorders, piles, purgative, stomach pain, ulcer, intestinal worms, vomiting, etc.)	312	219
General health (alterative, antiperiodic, prophylactic, etc.)	7	5
Gynaecological problems (menstrual disorders, pain, vaginal and uterine problems, etc.)	8	12
Haemorrhages (internal bleeding, nasal haemorrhage, etc.)	2	2
Hair care (prevent hair loss, scalp problems, lice)	7	8
Headache	18	20
Injuries (cuts and wounds, burns)	65	54
Mental disorders (Hysteria, insomnia, seizures, nervousness, etc.)	23	10
Musculoskeletal disorders (analgesic, arthritis, gouts, bone facture, rheumatism, body pain, joint pain, sprains, swellings, cramps, muscle relaxant, etc.)	76	84
Nervous system disorders (paralysis, hypertenson, etc.)	5	4
Nutritional disorders (weight loss, tonic, appetizers, etc.)	42	17
Odontological problems (tooth ache, gum problems, decayed teeth)	17	15
Ophthalmological disorders (eye wash, sore eyes, infection, etc.)	13	12
Poisoning (insect bites, leech bites, rabies, snake bites, bee stings, food intoxication)	25	26
Pregnancy, child birth, puerperium (labour induction, labour pain, after child birth, miscarriages, abortion, lactation stimulant, pregnancy prevention)	8	17
Respiratory system disorders (asthma, bronchitis, plague, chest pain, expectorant, pneumonia, tuberculosis, altitude sickness, nasal irritation)	65	55
Sexual health/dysfunction	7	8
Urinary system disorders (hematuria, kidney, urination, diuretic etc.)	26	27
Venereal diseases (gonorrhea, spermatorrhea etc.)	7	11

^a^Most taxa were reported to be used in more than one disease/ailment/condition category (see Table 1)

**Fig. 4:**
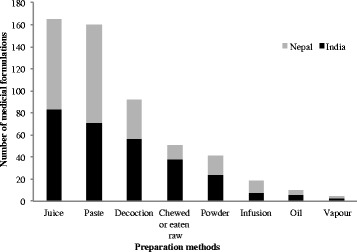
Use frequency (number of medicinal formulations) of different remedy preparation methods in India and Nepal

Mode of preparation included juice, paste, decoction, powder, infusion, and chewing raw plant parts (Fig. 4, Table 1). The majority of formulations were prepared as juice followed by paste and decoction. Proper selection of species, parts, as well as preparation and administration methods were very important in traditional health care systems.

**Fig. 5:**
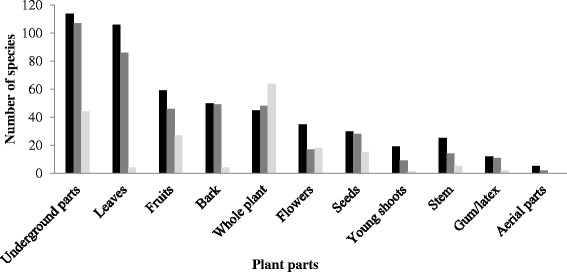
Use frequency (number of species) of different plant parts in traditional medicine preparation in India (black bars), Nepal (dark grey bars), and Bhutan (light grey bars)

Almost all plant parts were used to prepare different medicinal formulations: roots, rhizomes, tubers, bark, leaves, flowers, fruit, seeds, young shoots, whole plants, and gum and latex (Table 1). The most frequently used plant parts were underground parts, followed by leaves, fruit, bark, whole plants, seeds and flowers (Fig. 5). Use of multiple plant parts was often documented (Table 1). The preference for roots and rhizomes to prepare traditional remedies follows the scientific basis that roots generally contain high concentrations of bioactive compounds [36]. Such a trend is also reported from other studies from the Himalaya [35, 37, 38].

### NTFPs trade and livelihoods potential

The role of NTFPs is particularly important in the Himalayan region where a large proportion of the rural population depends on them as a source of medicine, food, fibre, dye and other useful materials [39–41]. In the Kangchenjunga Landscape, many of the NTFPs are used for subsistence, while others are the main or only source of income generation. However, the role of non-marketed NTFPs that were used for subsistence is largely ignorned when estimates are made of the economic importance of NTFPs to rural populations [42]. Understanding the economic value of non-marketed NTFPs helps to determine the true income of the gatherers and also helps ascertain the true value of the standing forest, leading to more rational decisions about its alternative uses [42].

Domestic as well as cross-border trade of NTFPs, both legally and illegally, is a historical practice in this region [43]. The traded NTFPs mostly include medicinal plants and to a lesser extent some wild edible plants and fibre yielding plants. The handmade paper from Argeli (*Edgeworthia gardneri*) is the only NTFPs that was sold after value addition in Nepal. Many of the species documented in this study possess high economic potential (Table 4) and could thus supplement family income [44] while generating incentives for biodiversity conservation [45].

**Table 4:** Major NTFPs traded (in kg) and revenue generated (USD) from 2008 to 2013 in the Nepal part of Kangchenjunga Landscape

Species/products and parts	Traded quantity (kg)	Revenue (USD)
Argeli (*Edgeworthia gardneri*)/Bark	97,000	4109
Ban lasun (*Fritillaria cirrhosa)/*Bulb	1500	150
Bish jara (*Aconitum ferox*)/Root	4300	301
Chiraito (*Swertia chirayita*)/Whole plant	88,765	7445.97
Chutro (*Berberis wallichiana*)/Bark	5000	NA
Daruhaldi (*Mahonia napaulensis*)/Bark	6500	130
Dhupi pat (*Juniperus indica*)/Twig	3800	76
Khayar (*Acacia catechu*)/Heartwood	97,784.6	38,456.88
Lauth salla (*Taxus wallichiana*)/Twig	290,500	9441.25
Lichen (*Usnea* sp., *Parmelia* sp.)/Whole plant	11,000	1650
Lokta (*Daphne bholua*)/Bark	71,076	1940
Majitho (*Rubia manjith*)/Whole plant	78,800	2199.24
Nagbeliko powder (*Lycopodium clavatum*)/Pollen grains	8000	160
Khoto (*Pinus roxburghii*)/Resin	1,256,334	9799.40
Ritha (*Sapindus mukorossi*)/Fruit	1600	48
Total	2,021,959.6	75,906.74

*Source*: Compiled from Hamro Ban - a yearly publication of Department of Forests, Ministry of Forests and Soil Conservation, Government of Nepal

**Fig. 6:**
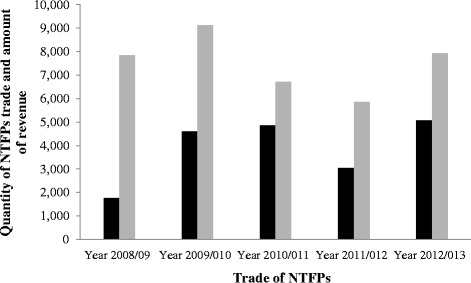
Amount of traded NTFPs (black bars) in ‘000 kg and revenue generated (grey bars) in USD in five years in the Nepalese part of the Kangchenjunga Landscape

Commonly traded NTFPs from the Nepal part of the Kangchenjunga Landscape include medicinal plants such as *Dactylorhiza hatagirea*, *Fritillaria cirrhosa, Neopicrorhiza scrophulariiflora*, lichens, and *Taxus wallichiana*. Other important species under trade are *Aconitum* species, *Valeriana jatamansi*, *Viscum album* and *Zanthoxylum* species. Species such as *Daphne bholua*, *Edgerworthia gardnerii, Rhododendron anthopogan*, *Rubia manjith*, *Swertia chirayita*, *Valeriana jatamansi*, and *Zanthoxylum* species are traded in large volume following legal procedures. The collected plant materials are normally sold to middlepersons (local traders), with only a few collectors selling or exporting NTFPs directly in local and cross-border markets. The total amount of NTFPs traded from Nepal in the last five years was 2,029,960 kg and the amout of revenue generated was around US$ 76,066 (Fig. 6, Table 4). The lack of openly accessible information on traded species of NTFPs from Bhutan and India limited our ability to conduct a comparative analysis.

Despite the high potential for trade and livelihoods through NTFPs, local people in the Kangchenjunga Landscape are not able to adequately benefit from engaging in the NTFP sector. In most cases, collectors were not aware of the market price for their products and were compelled to sell based on the offers of the middlepersons [46]. Thus, ensuring that market information is available to local people is one of the challenges in the NTFP sector in the landscape. Moreover, traders reported several other issues including multiple taxation system, hurdles during transportation, and duration of transport permit.

As reported by Sundriyal and Sundriyal [47] from Sikkim within the Kangchenjunga Landscape, the sale of fruit provides minimum returns due to fairly low shelf life and market costs. Therefore, some value addition in the form of pickle, chutney, jam, jelly, etc. may increase fruit shelf-life and economic profit to local communities. This reflects a clear need to diversify the product base and to ensure that wild edible plants fetch higher prices [47]. There is also need for value addition for other NTFPs. Therefore, value addition at the local level is an essential part of NTFP trade. Untapped but potential species of NTFPs such as wild edible fruit and vegetables could be promoted in local markets. These could also be promoted for visitors in hotels and restaurants.

Another major problem in commercialization of NTFPs is the low volume in which they are collected and produced, in contrast to the large quantities that are required for the markets. This problem could be addressed by establishing cooperatives, and using these cooperatives for collective marketing which will ensure optimum benefits to collectors [48].

### Threats and conservation challenges

Unustainable harvesting of NTFPs, mostly medicinal and edible plants, is the major threat to conservation and management of NTFPs in the Kangchenjunga Landscape [47, 49]. Sustainable harvesting is essential for conservation of NTFPs, and in turn for ensuring the livelihoods of many rural peoples. Indeed, promotion of commercial extraction of NTFPs as a conservation strategy is based on the argument that forest conservation must be able to offer economic incentives to local peoples in order to counter the threat from destructive land uses such as logging and grazing. This strategy has gained wide acceptance as a conservation paradigm [2]. As indicated by Ticktin [2], despite growing concern over the conservation of these species, as well as their potential to enhance forest conservation and livelihoods, information on the ecological implications of harvest is not available in the Kangchenjunga Landscape.

Illegal trade of NTFPs from the landscape often includes some of the CITES Appendix listed species such as Sunakhari (Orchids), Kutki (*Neopicrorhiza scrophulariiflora*), and Lauth salla (*Taxus wallichiana*). The trade also includes some plant species under legal protection of the Government of Nepal like Orchids, Champ (*Michelia champaca*), Jhyau (Lichens), Jatamansi (*Nardostachys grandiflora*), and Sughandhawal (*Valeriana jatamansi*) [43]. These species are mostly traded to India via local collectors, whereas limited quantity of these items are exported to Tibet [50, 51]. Conserving such species is challenging, yet illegal trade has slightly decreased in the last decade due to effective conservation efforts of local organizations and increased cultivation practices in the landscape. Community forestry, which has restricted open access to NTFPs, and resource monitoring have also been effective in conserving NTFPs in recent years. In addition, availability of economically important NTFP species has currently declined due to deforestation and replacement with monoculture, use of pesticides and over harvesting [50]. Traditional knowledge on the use of NTFPs such as medicinal plants is also gradually declining due to socio-economic transformation in the Kangchenjunga Landscape [49, 52, 53].

Monitoring is one of the key components to promote the NTFP sector. Follow-up of rules, regulations and strategies related to NTFPs is necessary for contributing to changes in policy that are able to mainstream sustainable management of NTFPs with livelihoods improvement. Limited progress has been achieved in the Kangchenjunga Landscape in controling over-harvesting, enforcing effective harvesting regimes, and maintaining conducive and adaptive adminstrative processess. Recently adopted economic tools such as certification of sustainable harvests should also be applied as a means of ensuring that NTFPs collected sustainably can be identified as such by the consumers [54, 55].

NTFPs reported from the Kangchenjunga Landscape also include many species under different threat categories as well as under priorities of the governments. For example, of the total 30 national priority herbs of Nepal, 26 are abundantly available in the Kangchenjunga Landscape, while all species prioritized for cultivation and research in Nepal are also reported from the Landscape [56]. Among these, *Nardostachys grandiflora, Neopicrorhiza scrophulariiflora, Rauvolfia serpentina* and *Taxus wallichiana* are the most threatened species. Therefore, the economic, socio-cultural and conservation values of these NTFPs are extremely high.

### NTFP policy frameworks

A comparision of NTFP policy frameworks in the Kangchenjunga Landscape shows that Bhutan, India and Nepal have supportive policies for the NTFP sector, thereby providing enabling environments and support for NTFP programs and marketing [25]. As a result, many development agencies including national and international non-governmental organizations have placed emphasis on NTFPs in their programs. The collection, conservation and sustainable utilization of NTFPs in Bhutan is mostly guided by the National Strategy for the Development of Non-Wood Forest Products 2008–2018. Other sectoral policies are the Forest Act 1969, Plant Quarantine Act of Bhutan 1993, Forest and Nature Conservation Act of Bhutan 1995, Environmental Assessment Act 2000 and Biodiversity Act and Framework of Bhutan 2003, 2006 [57]. The Indian National Forest Policy (1988) makes a special mention of NTFPs emphasizing on protection, improvement and their enhanced production for generation of employment and income [58]. Likewise, in Nepal there are several sectoral as well as specific policy provisions for sustainable use and management of NTFPs [15, 55]. The most comprehensive policy is the Herbs and Non-Timber Forest Product Development Policy 2004 [59]. The recent Nepal National Biodiversity Strategy and Action Plan 2014 and Forest Policy 2015 also emphasize sustainable use and management of NTFPs and critically provide special opportunity to support livelihoods of marginalized propoor and women through wise use of NTFP. Nevertheless, present policy formation, implementation and field reality reflects power structures and domination by certain stakeholders and interests [60].

Gender participation in policy formulation is also challenging. For example, 75 % of people collecting NTFPs in India were women and 100 % involved in NTFP processing were women, but their inclusion in Joint Forest Management committees was less than 10 % [61]. Similarly, in Nepal, although women contribute a large share of the labor for forest and biodiversity conservation in community forests, they represent only 22 % in the executive bodies of Community Forest User Groups [62]. Similar situation exists in Bhutan where the women’s involvement is generally low in the designing, planning, and implementation of forestry policies, and there is limited understanding of the roles, knowledge, aspirations and contributions made by women towards NTFP management [63].

Considerable efforts have been made to develop the NTFP sector, but the contribution of NTFPs in national economies remains insignificant. As pointed by Shackleton and Pandey [21], the reason behind this is that their economic value remains invisible to external observers as most NTFPs are used for household purposes; production and harvest of NTFPs is a seasonal event, with their use or trade involving only small quantities; much of the NTFP trade is via informal and closed markets which are hard to enumerate; production and markets is dispersed; and their use is highest in rural areas, which are often remote and marginalised in terms of human resources and development policies.

### Gaps on knowledge based conservation and management of NTFPs

Like in other parts of the Himalayas, there is still a severe paucity of in-depth field based information on the abundance, reproductive biology and ecological impacts of harvesting of NTFPs in the Kangchenjunga Landscape. There is no standard method available to estimate the economic contribution of NTFPs and their products. Research on the inventory, life history of NTFPs, and impact of harvesting on the ecosystem is a prerequiste for their sustainable management, yet very few such activities have been documented from the landscape [64]. Similarly, ecological impacts of NTFPs harvesting for domestic and commercial purposes must be estimated to ensure their sustainability and the implementation for effective conservation measures.

Market size, structure and value chain of NTFP species depend on the demand and supply characteristics of products and their beneficiaries in different areas. Understanding market information is important for value addition and in devising investment strategies [16] for NTFPs based products, their diversification, and related enterprises. At present, the majority of NTFPs from the Kangchenjunga Landscape are traded in the raw forms, and NTFP harvesters lack necessary support for market-based strategies from both private, as well as government sectors.

Indigenous knowledge and management systems have been recognized for contributing to sustainable use of NTFPs, and consequently they have secured legal rights to manage forest resources [55]. In the Kangchenjunga Landscape, indigenous knowledge on NTFP use is well documented, but indigenous management systems need to be assessed and used for sustainable management.

## Conclusion

We documented NTFPs collected and used for various purposes by the local people of the Kangchenjunga Landscape. The diversity of NTFPs was highest in India, followed by Nepal and Bhutan. Though the landscape possesses many potential species for trade, their nominal contribution to local livelihoods was due to lack of value addition and commercialization. Unsustainable harvesting and lack of value addition and commercialization could be considered as major challenges for conservation and development of the NTFP sector in the landscape.

Tracing the trend of NTFP research and exploitation, it shows much focus on medicinal plants resulting in over-harvesting of some highly potential medicinal plants, with very negligible amount of other plant species reported for other purposes. At present, NTFPs are synonymous with medicinal and aromatic plants and vice versa. Only small amounts of other NTFPs are marketed. Therefore, research must also focus on other potential categories of NTFPs. NTFP collectors need to be educated about forest ecology and the adverse impacts of unsustainable harvesting for conservation and local livelihoods. Sustainable harvesting techniques should be provided through training and capacity building programs to local people. Biological studies of high value NTFPs must be carried out in order to ensure sustainability of these resources.

Phytochemical screening of medicinal plants and nutrient value analysis of wild edible plants would foster their commercialization. Traditional knowledge of medicinal plant use could be integrated with ‘modern’ health care systems [65]. Highly potential NTFPs must be identified and grown for commercial cultivation and adopted in traditional agroforestry systems. This will reduce pressure on these species in their natural environments while providing economic benefits to poor farmers [47].

Conservation and development organizations, together with government agencies and private sectors, must provide technical and innovative inputs to add value to NTFP products. They must also facilitate community mobilization for assessment and identification of potential NTFPs. The latter role will be of significant importance considering the limited human and financial resources of government agencies in the Kangchenjunga Landscape. An integrated approach will promote sustainable use of NTFPs while contributing to income generation and livelihood improvement for local people. Transboundary landscape conservation programs will provide opportunities for transboundary cooperation through policy reforms, as well as providing opportunities to diversify livelihoods of forest dependent communities. However, marketing and commercialization of NTFPs can be successful only if the activity is transparent, equitable and sustainable, with important implications for poverty reduction and better resource management [20]. Increasing access to NTFP-selling outlets could be achieved through information dissemination, empowerment of collectors and establishment of linkages between collectors and traders [41]. The role of small and medium sized enterprises and cooperatives is extremely important to achieve sustainable management of NTFPs.
