# Erratum to: Traditional use and management of NTFPs in Kangchenjunga Landscape: implications for conservation and livelihoods

**DOI:** 10.1186/s13002-017-0152-0

**Published:** 2017-05-05

**Authors:** Yadav Uprety, Ram C. Poudel, Janita Gurung, Nakul Chettri, Ram P. Chaudhary

**Affiliations:** 1 Research Centre for Applied Science and Technology, Tribhuvan University, Kirtipur, Kathmandu, Nepal; 2 Nepal Academy of Science and Technology (NAST), Khumaltar, Lalitpur, Nepal; 3 International Centre for Integrated Mountain Development, Khumaltar, Lalitpur, Nepal; 4 Central Department of Botany, Tribhuvan University, Kirtipur, Kathmandu, Nepal

## Erratum

In the original publication [1] the species name Kutki (*Neopicrorhiza scrophulariiflora*) is wrongly mentioned as CITES listed species. The correct version of the sentence in the second paragraph of “Threats and conservation challenges” can be found here:

Illegal trade of NTFPs from the landscape often includes some of the CITES Appendix listed species such as Sunakhari (Orchids) and Lauth salla (*Taxus wallichiana*).
